# Global, regional, and national burden of hip fractures attributable to falls in older adults: changes from 1990–2021 and 2036 projections

**DOI:** 10.3389/fpubh.2025.1674881

**Published:** 2025-09-18

**Authors:** Binbin Zhang, Bin Dou, Kewen Li

**Affiliations:** Department of Orthopedics, Qinghai University Affiliated Hospital, The Clinical Medical College of Qinghai University, Xining, China

**Keywords:** falls, hip fractures, global burden of disease, prediction, older adults

## Abstract

**Background:**

Hip fractures attributable to falls (HFAF) represent one of the life-threatening conditions in older adults. This study aims to analyze trends and geographical distribution characteristics of HFAF in older adults from 1990 to 2021.

**Methods:**

Key indicators including incidence, prevalence, and years lived with disability (YLDs) were extracted from the Global Burden of Disease (GBD) 2021 database. Spatiotemporal trends in disease burden were systematically described using methods including Joinpoint regression analysis, age-period-cohort analysis, inequality analysis, and frontier analysis. Decomposition analysis was used to explore key factors underlying fluctuations in HFAF burden in older adults. Finally, the ARIMA model was employed to project disease burden over the next 15 years.

**Result:**

Globally, from 1990 to 2021, age-standardized prevalence (ASPR) and incidence (ASIR) of HFAF in older adults increased, whereas age-standardized YLDs (ASYR) decreased. Disease burden was greater in regions with higher socioeconomic status. Gender-based analysis showed that disease burden was greater among older women than among older men. Over time, cross-country inequalities widened. Frontier analysis identified that countries such as Belgium and Switzerland have substantial potential to improve disease control. Decomposition analysis identified population growth as the primary driver of the rising global burden. Projections indicated that case numbers will continue to rise by 2036, but age-standardized rates will decline.

**Conclusion:**

This study indicates that the global disease burden of HFAF in older adults increased from 1990 to 2021 and is projected to continue rising through 2036. Reductions in ASYR suggest that current treatment measures are effective.

## Introduction

Globally, falls have emerged as an unignorable critical issue in public health. Data released by the World Health Organization (WHO) in 2021 indicate that falls are the second leading cause of unintentional injury-related deaths worldwide. Annually, 37.3 million severe falls requiring medical attention occur globally, resulting in approximately 684,000 deaths (1). Among these, adults aged ≥60 years account for the largest proportion of fatal fall-related injuries: roughly one in three individuals aged ≥65 years experiences at least one fall yearly. For those aged ≥80 years, this proportion rises to one in two, earning falls the label of a “killer of older adults” (2). A 2018 telephone survey across 50 U.S. states found that 27.5% of community-dwelling adults aged ≥65 years nationwide (*n* = 13,685,662) reported falling (3). In the United States, fall-related deaths among adults aged ≥65 years increased from 29.4 per 100,000 population in 1990 to 69.4 per 100,000 in 2020 (4). In the Netherlands, fall-related deaths among adults aged ≥80 years rose from 391 cases in 2000 to 2501 cases in 2016 (5). Annual fall-related healthcare expenditures in the United States exceed 80 billion, including 53.3 billion in Medicare spending, 3.5 billion in Medicaid spending, and 23.2 billion in private/out-of-pocket/other expenditures (6). With accelerating global population aging, fall-related health issues among older adults have become a core challenge in public health.

Hip fractures are one of the most threatening of the serious consequences of falls in the older adults (7, 8). Owing to osteoporosis, reduced muscle strength, and impaired balance, older adults are highly susceptible to hip fractures when falling. Hip fractures not only cause severe pain but also lead to prolonged bed rest, triggering serious complications including hypostatic pneumonia, pressure ulcers, and deep vein thrombosis. Data indicate that the 1-year post-operative mortality rate among older adults with hip fractures is as high as 20%−30%, significantly exceeding that of many chronic diseases and imposing a heavy burden on families and healthcare systems (9, 10). Cross-national studies have found that economic costs associated with hip fractures have increased over the past decade, with treatment costs rising with patient age and hospitalization expenses accounting for a large proportion of total costs in older patients (11–13). In this context, in-depth exploration of the global epidemiological characteristics of hip fractures attributable to falls (HFAF) in older adults is urgent, as it has significant implications for formulating effective prevention strategies, rationally allocating healthcare resources, and improving older populations' health status.

In recent years, the academic community has conducted several regional studies on HFAF in older adults. For instance, between 1990 and 2019, the age-standardized incidence rate of HFAF decreased among women in Central European countries, whereas it increased among men (14). In 2019, U.S. adults aged ≥65 years had 318,797 emergency department visits, 290,130 hospitalizations, and 7,731 deaths related to hip fractures. Approximately 88% of emergency department visits and hospitalizations, and 83% of hip fracture-related deaths, were attributed to falls (15). However, existing studies have significant limitations: most focus on high-income countries, with insufficient coverage of low- and middle-income regions, thus failing to reflect the global distribution of HFAF in older adults. Additionally, variations in diagnostic criteria and data collection methods across studies reduce result comparability. Therefore, this study aims to analyze the global epidemiological characteristics of HFAF in older adults using specific methods.

The Global Burden of Disease (GBD) study, led by the Institute for Health Metrics and Evaluation at the University of Washington (USA) with participation from multiple countries and regions worldwide, is a comprehensive epidemiological survey project (16). This dataset includes indicators of how various diseases, injuries, and risk factors impact human health, and is currently a highly representative data source for investigating disease epidemiological trends (17–19). Using the GBD 2021 database, the present study aims to systematically analyze the prevalence, incidence, and years lived with disability (YLD) of HFAF in older adults from 1990 to 2021 across the globe, five Socio-demographic Index (SDI) regions, 21 GBD regions, and 204 countries. It will also explore distribution characteristics and temporal trends across different age groups and genders. Furthermore, through health inequality analysis, decomposition analysis, age-period-cohort analysis, and ARIMA model prediction, this study will further reveal: how cross-national disparities in the disease burden of HFAF in older adults have evolved over time; which socioeconomic and health factors contribute to these disparities; and which countries have suboptimal prevention and control of HFAF in older adults given their existing resources and technical conditions. This study will deepen understanding of the epidemiological trends of HFAF in older adults, provide data support for countries in formulating prevention, intervention, and health service policies related to such fractures, and facilitate global healthcare resource allocation.

## 2 Methods and data

### 2.1 Data source

The GBD 2021 provides disease burden data for 369 diseases and injuries across 204 countries and territories (16). In this study, data on the disease burden of HFAF in older adults were extracted from GBD 2021 via the Global Health Data Exchange (GHDx) Results Tool (https://vizhub.healthdata.org/gbd-results/). HFAF in older adults were defined as the etiological factor, with “prevalence,” “incidence,” and “YLDs” as measurement metrics. Data were further retrieved by selecting different geographic locations (global, regional, and national), genders, and age groups (>55 years). Detailed retrieval methods are provided in Supplementary Figure S1.

### 2.2 Definitions

The Socio-demographic Index (SDI) is an evaluation metric developed by GBD Collaborators, based on national/territorial rankings of per capita income, average educational attainment, and total fertility rate. It measures the socio-demographic development level of individual countries or territories, with values ranging from 0 to 1, where higher values indicate a higher level of socio-demographic development. Using SDI, GBD Collaborators categorized 204 countries/territories worldwide into five SDI regions: Low SDI (<0.466), Low-middle SDI (0.466–0.619), Middle SDI (0.620–0.712), High-middle SDI (0.713–0.810), and High SDI (>0.810) (20). To eliminate the influence of age distribution differences across populations on analytical results, prevalence, incidence, and YLDs were age-standardized in this study. The specific method was based on the world standard population age structure provided in the GBD 2021 report, with the calculation formula as follows:


$$\begin{array}{l} Age-standardized\;=\;\frac{\sum_{i=1}^{A}a_{i}w_{i}}{\sum_{i=1}^{A}w_{i}}\;\times 100,000 \end{array}$$

*a*_*i*_ being the age-specific rate in the i^th^ age group, *w*_*i*_ being the number of people in the i^th^ corresponding age group among the standard population and *A* being the number of age groups (21).

### 2.3 Descriptive analysis

This section primarily describes the age-standardized incidence rate (ASIR), age-standardized prevalence rate (ASPR), and age-standardized YLD rate (ASYR) of HFAF in older adults in 2021, across the globe, five SDI regions, 21 GBD regions, and 204 countries. The estimated annual percentage change (EAPC) was also calculated to analyze trends in the disease burden of HFAF in older adults from 1990 to 2021. The EAPC is derived by fitting a regression model to the natural logarithm of the rate, with time as the variable. Specifically, the natural logarithm of each observed value is fitted to a straight line, and the EAPC is calculated using the slope of this line, with the specific formula as follows:


$$\begin{array}{l} y\;=\;\alpha +\beta x+\varepsilon \end{array}$$

$$\begin{array}{l} EAPC\left(\%\right)\;=\;100\times \left(exp\left(\beta \right)-1\right) \end{array}$$

Where x represents the year, y represents the natural logarithm of the age-standardized rate, α represents the intercept, β represents the slope, and ε represents the random error (22, 23).

### 2.4 Joinpoint regression analysis

Further, Joinpoint regression analysis was used to analyze trends in the ASIR, ASPR, and ASYR of HFAF in older adults globally. The annual percent change (APC) and average annual percent change (AAPC) were calculated to analyze changes across multiple stages. A positive AAPC indicates an annual increase in disease burden, whereas a negative AAPC indicates an annual decrease; an AAPC of 0 indicates a monotonic trend. The maximum number of joinpoints selected was 5 (24).


$$\begin{array}{l} APC\;=\;\left[exp\left(\beta \right)-1\right]\times 100 \end{array}$$

$$\begin{array}{l} AAPC\;=\;\left[exp\;\left(\frac{\sum w_{i}b_{i}}{\sum w_{i}}\right)-1\right]\times 100 \end{array}$$

b_i_ is the slope coefficient of the i-th segment. Here, i indexes each segment within the required year range. The w of the segment interval weights the regression coefficients of each interval. w_i_ represents the interval width of each piecewise function (i.e., the number of years included in the interval), and β is the regression coefficient corresponding to each interval.

### 2.5 Decomposition analysis

Das Gupta's decomposition analysis method was used to explore the relative impacts of three factors—aging, population growth, and epidemiological changes—on changes in the disease burden of HFAF in older adults from 1990 to 2021. The sum of the contribution rates of these three factors within the same region equals 100% (25).

### 2.6 Cross-country inequality analysis

The slope index of inequality (SII) and health inequality concentration index (CI) were used to assess the unequal distribution of the burden of HFAF in older adults across countries. The SII directly measures the absolute burden difference between extreme SDI groups: a positive value indicates a greater disease burden in high-SDI countries, a negative value indicates a greater burden in low-SDI countries, and its absolute value is positively correlated with the degree of inequality. The concentration index is calculated using Lorenz curve plots (ranging from −1 to 1); a negative value indicates that the health burden is concentrated in low-SDI countries, and the absolute value reflects the degree of inequity in social stratification. These two indicators form a comprehensive evaluation system covering absolute difference and relative distribution (26).

### 2.7 Age-period-cohort analysis

The Poisson-based age-period-cohort model was used to explore the effects of age, period, and birth cohort on HFAF in older adults. This model is currently widely applied in demography, epidemiology, sociology, and other fields (27). Local drift represents the log-linear trend of specific periods and birth cohorts within each age group, reflecting the total age-standardized proportion of expected age-specific rates over time. The age effect refers to differences in the disease burden of HFAF among different age groups of older adults. The period effect reflects changes in the disease burden of HFAF during specific time periods. The cohort effect refers to the impact of unique environmental exposures or experiences of a birth cohort on the disease burden of HFAF in older adults. In this study, age groups were divided into nine categories at 5-year intervals: “55–59 years,” “60–64 years,” “65–69 years,” “70–74 years,” “75–79 years,” “80–84 years,” “85–89 years,” “90–94 years,” and “95+ years.” Periods were divided into six groups: 1992–1996, 1997–2001, 2002–2006, 2007–2011, 2012–2016, and 2017–2021. Birth cohorts were calculated as period minus age, including five partially overlapping 10-year birth cohorts (referenced by the mid-year of birth), ranging from 1893–1901 (the 1897 cohort) to 1958–1966 (the 1962 cohort) (Supplementary Table S1). This method has been validated as effective in previous studies (28).

### 2.8 Frontier analysis

After clarifying the current situation, trends, and influencing factors, frontier analysis was used to evaluate the optimization potential of the disease burden of HFAF in older adults. An SDI-based optimal practice boundary was used to calculate the “effective difference” between the observed value of each country/region and the theoretical optimal value. The distance between each country/region's representative point and the boundary is termed the “effective difference,” indicating the gap between the observed disease burden of HFAF in older adults and the minimum achievable burden for a country/region given its SDI. This indicator reflects the potential reduction in the disease burden of such fractures achievable through optimized resource allocation under specific sociodemographic conditions (29).

### 2.9 Forecasting analysis

The autoregressive integrated moving average (ARIMA) model was used to predict future trends of the disease burden of HFAF in older adults from 2022 to 2036. The model is denoted as ARIMA (p, d, q), where p is the autoregressive order, d is the number of differences, and q is the moving average order. The optimal model was selected based on the Akaike information criterion (AIC) value: models were searched according to the provided constraint order, and the optimal one was determined. The Ljung-Box test for white noise was used to examine residual serial correlation. This model has been applied in numerous disease prediction studies (30, 31).

## 3 Results

### 3.1 Increased global disease burden of HFAF in older adults

Globally, from 1990 to 2021, the age-standardized incidence rate (ASIR) of HFAF in older adults increased from 847.64 per 100,000 population (95% UI: 559.84, 1,222.97) to 930.73 per 100,000 (95% UI: 624.14, 1,317.39), representing a total increase of 9.8% over 32 years (EAPC = 0.28, 95% CI: 0.25, 0.32; Table 1). The age-standardized prevalence rate (ASPR) rose from 1,606.23 per 100,000 (95% UI: 1,274.92, 2,019.73) to 1,834.14 per 100,000 (95% UI: 1,470.71, 2,274.39), with a larger total increase of 14.2% (EAPC = 0.48, 95% CI: 0.43, 0.52). In contrast, the age-standardized YLD rate (ASYR) decreased slightly by 7.2% [from 177.19 per 100,000 [95% UI: 118.04, 251.3] to 164.5 per 100,000 (95% UI: 109.93, 233.22); EAPC = −0.18, 95% CI: −0.22, −0.14] (Table 1).

**Table 1:** ASIR, ASPR, and ASYR in 1990 and 2021, and EAPC from 1990 to 2021 at the global level and different regions.

**Location**	**ASIR per 100,000 population, 1990**	**ASIR per 100,000 population, 2021**	**EAPC of ASIR**	**ASPR per 100,000 population, 1990**	**ASPR per 100,000 population, 2021**	**EAPC of ASPR**	**ASYR per 100,000 population, 1990**	**ASYR per 100,000 population, 2021**	**EAPC of ASYR**
Global	847.64 (559.84, 1,222.97)	930.73 (624.14, 1,317.39)	0.28 (0.25, 0.32)	1,606.23 (1,274.92, 2,019.73)	1,834.14 (1,470.71, 2,274.39)	0.48 (0.43, 0.52)	177.19 (118.04, 251.3)	164.5 (109.93, 233.22)	−0.18 (−0.22,−0.14)
**Sex**									
Female	1,060.7 (699.29,1,533.1)	1,129.07 (754.2, 1,607.57)	0.19 (0.15, 0.23)	2,072.51 (1,644.05, 2,604.8)	2,313.46 (1,854.56, 2,870.17)	0.4 (0.37, 0.43)	225.96 (150.65, 320.58)	207.9 (139.02, 294.47)	−0.19 (−0.23, −0.16)
Male	481.23 (313.67, 704.35)	635.76 (426.75, 904.48)	0.91 (0.84, 0.97)	828.23 (647.59, 1,060.12)	1,141.48 (908.43, 1,426.3)	1.13 (1.05, 1.22)	98.06 (64.81, 139.81)	103.08 (68.38, 147.34)	0.2 (0.14, 0.26)
**SDI regions**									
High SDI	1,312.11 (886.91, 1,844.73)	1,469.17 (1,013.83, 2,039.11)	0.46 (0.4, 0.52)	2,791.61 (2,225.12, 3,489.53)	3,421.55 (2,759.5, 4,210.12)	0.77 (0.7, 0.85)	231.56 (154.31, 327.92)	236.3 (154.2, 339.97)	0.33 (0.25, 0.4)
High-middle SDI	721.82 (465.25, 1,070.6)	778.49 (517.38, 1,117.05)	0.07 (−0.02, 0.16)	1352.48 (1,062.63,1,715.48)	1,477.82 (1,180.6, 1,842.19)	0.23 (0.18, 0.28)	151.15 (100.87, 214.93)	116.07 (75.91, 167.87)	−1.04 (−1.15, −0.93)
Middle SDI	413.54 (251.23, 645.72)	639.98 (403.63,959.32)	1.22 (1.02, 1.42)	624.65 (474.18, 822.57)	1,009.64 (779.57, 1,304.04)	1.44 (1.26, 1.61)	128.18 (83.35, 184.67)	123.85 (81.74, 176.74)	−0.29 (−0.41,−0.17)
Low-middle SDI	514.2 (305.26, 812.52)	632.05 (396.82, 951.91)	0.62 (0.55, 0.68)	682.03 (505.62, 920.24)	961.67 (731.94, 1,255.97)	1.14 (1.04, 1.23)	161.39 (102.95,236.53)	174.59 (113.49, 248.28)	0.24 (0.1, 0.37)
Low SDI	349.48 (214.35, 539.27)	464.74 (293.86, 693.83)	0.86 (0.81, 0.92)	459.15 (342.86, 612.47)	693.26 (526.32, 905.06)	1.36 (1.29, 1.44)	115.4 (73.5, 168.51)	140.55 (91.62, 200.8)	0.64 (0.55, 0.73)
**GBD regions**									
Andean Latin America	153.41 (100.36, 220.59)	238.1 (158.16, 337.8)	1.37 (1.3, 1.44)	281.85 (224.94, 353.35)	445.35 (352.94, 558.08)	1.43 (1.31,1.54)	62.83 (41.63, 88.82)	61.46 (41.15, 85.91)	−0.18 (−0.28, −0.07)
Australasia	1,606.97 (1,109.79, 2,204.07)	2,358.9 (1,676.77, 3,151.75)	1.71 (1.54,1.88)	3,182.39 (2,559.01, 3,910.96)	4,725.35 (3,901.08, 5,685.83)	1.71 (1.54, 1.88)	230.58 (151.67, 331.43)	338.62 (225.18, 484.8)	1.68 (1.51, 1.84)
Caribbean	511.63 (349.57, 720.07)	808.03 (557.69, 1,109.79)	1.38 (1.26, 1.51)	782.09 (623.54, 976.41)	1,273.59 (1,022.35, 1,560.2)	1.5 (1.36, 1.64)	121.3 (80.45, 170.22)	134.4 (88.86, 188.7)	0.27 (0.18, 0.37)
Central Asia	122.83 (80.39, 177.83)	165.74 (111.1, 236.12)	1.53 (1.31,1.76)	247.15 (196.39, 310.08)	284.67 (225.65, 357.52)	1.02 (0.73, 1.31)	44.37 (29.83, 62.19)	39.62 (26.51, 55.42)	0.06 (−0.12, 0.24)
Central Europe	1177.31 (800.56, 1,668.37)	902.44 (608.36, 1,277.38)	−1.11 (−1.2, −1.03)	1,718.37 (1,343.29, 2,179.52)	1,478.32 (1,166.93, 1,855.97)	−0.65 (−0.72, −0.58)	237.83 (157.99, 335.07)	117.66 (75.58, 171.63)	−2.44 (−2.55, −2.33)
Central Latin America	493.18 (311.96, 740.62)	400.49 (262.17, 582.09)	−0.69 (−0.82, −0.56)	857.08 (665.09, 1,106.21)	718.23 (564.59, 910.26)	−0.5 (−0.61, −0.39)	169.59 (112.34, 240.35)	99.08 (66.2, 138.93)	−1.62 (−1.76, – 1.48)
Central Sub-Saharan Africa	185.97 (120.62, 270.38)	250.06 (165.8, 357.62)	0.9 (0.8, 1)	246.91 (188.45, 319.12)	360.53 (278.59, 457.52)	1.24 (1.1, 1.39)	63.64 (41.02, 92.53)	80.02 (52.07, 114.37)	0.77 (0.69, 0.85)
East Asia	383.44 (225.21, 610.04)	729.25 (454.35, 1,099.14)	1.75 (1.24, 2.27)	571.95 (429.16, 763.1)	1,121.95 (860.59, 1,453.4)	2.01 (1.56, 2.47)	108.25 (69.94,157.36)	96.32 (61.41, 142.44)	−0.99 (−1.54, −0.44)
Eastern Europe	301.05 (179.15, 472.88)	349.37 (215.99, 530.75)	0.48 (0.3, 0.66)	595.45 (456.91, 779.52)	689.41 (531.98, 893.66)	0.65 (0.52, 0.79)	82.27 (54.9, 117.57)	61.49 (40.04, 88.53)	−1.06 (−1.39, −0.74)
Eastern Sub-Saharan Africa	199.12 (125.75, 295.62)	264.42 (173.18, 382.9)	0.85 (0.8, 0.9)	263.79 (200.43, 346.71)	386.42 (297.76, 498.08)	1.31 (1.22, 1.39)	66.33 (42.84, 96.65)	82.24 (53.92, 117.42)	0.78 (0.72, 0.85)
High-income Asia Pacific	758.25 (463.52, 1,164.82)	821.59 (542.76, 1,180.45)	0.16 (0.02, 0.29)	1,902.65 (1,476.84, 2,438.37)	2,186.04 (1,749.28, 2,707.11)	0.33 (0.15, 0.51)	151.87 (99.41, 218.55)	147.59 (95.03, 213.37)	−0.18 (−0.26, −0.1)
High-income North America	1,059.81 (660.84, 1,603)	1,628.82 (1,049.14, 2,361.69)	1.47 (1.21, 1.72)	2,504.15 (1,931.91, 3,225.96)	4,093.01 (3,216.69, 5,163.03)	1.78 (1.62, 1.93)	182.37 (117.32, 267.14)	275.09 (176.07, 398.59)	1.56 (1.4, 1.71)
North Africa and Middle East	190.89 (122.22, 280.58)	344.84 (228.05, 490.58)	2.19 (2.05, 2.34)	300.99 (235.27, 384.51)	558.54 (441.82, 700.33)	2.2 (2.04, 2.37)	59.78 (39.39, 84.2)	63.14 (41.85, 88.47)	0.24 (0.09, 0.4)
Oceania	351.2 (222.87, 522.97)	520.72 (340.69, 755.43)	1.11 (0.98, 1.24)	476.2 (364.33, 621.36)	763.84 (596.1, 962.09)	1.4 (1.29, 1.5)	107.64 (69.48, 155.74)	159.23 (103.25, 225.15)	1.18 (1.07, 1.29)
South Asia	753.23 (441.47, 1,202.49)	946.17 (585.09, 1,438.7)	0.63 (0.57, 0.69)	956.01 (701.33, 1,301.7)	1,418.24 (1,073.26, 1,866.94)	1.28 (1.2, 1.36)	225.39 (142.89, 332.69)	244.51 (158.88, 349.19)	0.2 (0.06, 0.33)
Southeast Asia	360.55 (225.91, 543.18)	481.08 (314.61, 696.14)	0.87 (0.81, 0.92)	556.35 (426.16, 729.28)	775.4 (608.43, 987.28)	1 (0.94, 1.06)	118.32 (77.08, 169.99)	114.54 (75.65, 161.29)	−0.25 (−0.29, −0.2)
Southern Latin America	618.61 (431.16, 849.52)	725.07 (511.65, 977.89)	0.63 (0.49, 0.78)	1,358.92 (1,089.15, 1,680.59)	1,675.07 (1,361.15, 2,042.89)	0.74 (0.67, 0.81)	226.51 (152.2, 315.01)	164.92 (111.62, 227.93)	−0.99 (−1.09, −0.9)
Southern Sub-Saharan Africa	123.29 (75.93, 189.64)	120.06 (75.99, 179.82)	−0.47 (−0.6, −0.34)	209.26 (160.86, 270.73)	169.48 (129.11, 222.01)	−1.01 (−1.2, −0.83)	43.61 (28.56, 62.7)	30.88 (20.17, 44.3)	−1.46 (−1.56, −1.35)
Tropical Latin America	450.73 (265.02, 712.77)	523.73 (325.24, 793.13)	0.56 (0.53, 0.58)	771.62 (588.65, 1,012.45)	994.63 (777.05, 1,270.49)	0.85 (0.77, 0.93)	152.98 (100.53, 219.88)	138.59 (92.92, 195.75)	−0.27 (−0.35, −0.2)
Western Europe	1,756.22 (1,213.24, 2,414.97)	1,733.8 (1,230.39, 2,332.91)	0.06 (0.01, 0.12)	3,626.39 (2,922.96, 4,477.03)	3,961.89 (3,252.74, 4,788.81)	0.4 (0.34, 0.45)	297.32 (198.86, 419.64)	275.02 (181.78, 393.31)	0.08 (−0.04, 0.2)
Western Sub-Saharan Africa	244.47 (153.51, 368.74)	317.88 (205.03, 464.67)	0.83 (0.78, 0.88)	345.07 (260.96, 454.2)	471.06 (362.65, 606.26)	1.01 (0.93, 1.08)	81.92 (52.89, 119.33)	94.44 (61.78, 135.16)	0.45 (0.41, 0.49)

**Figure 1:**
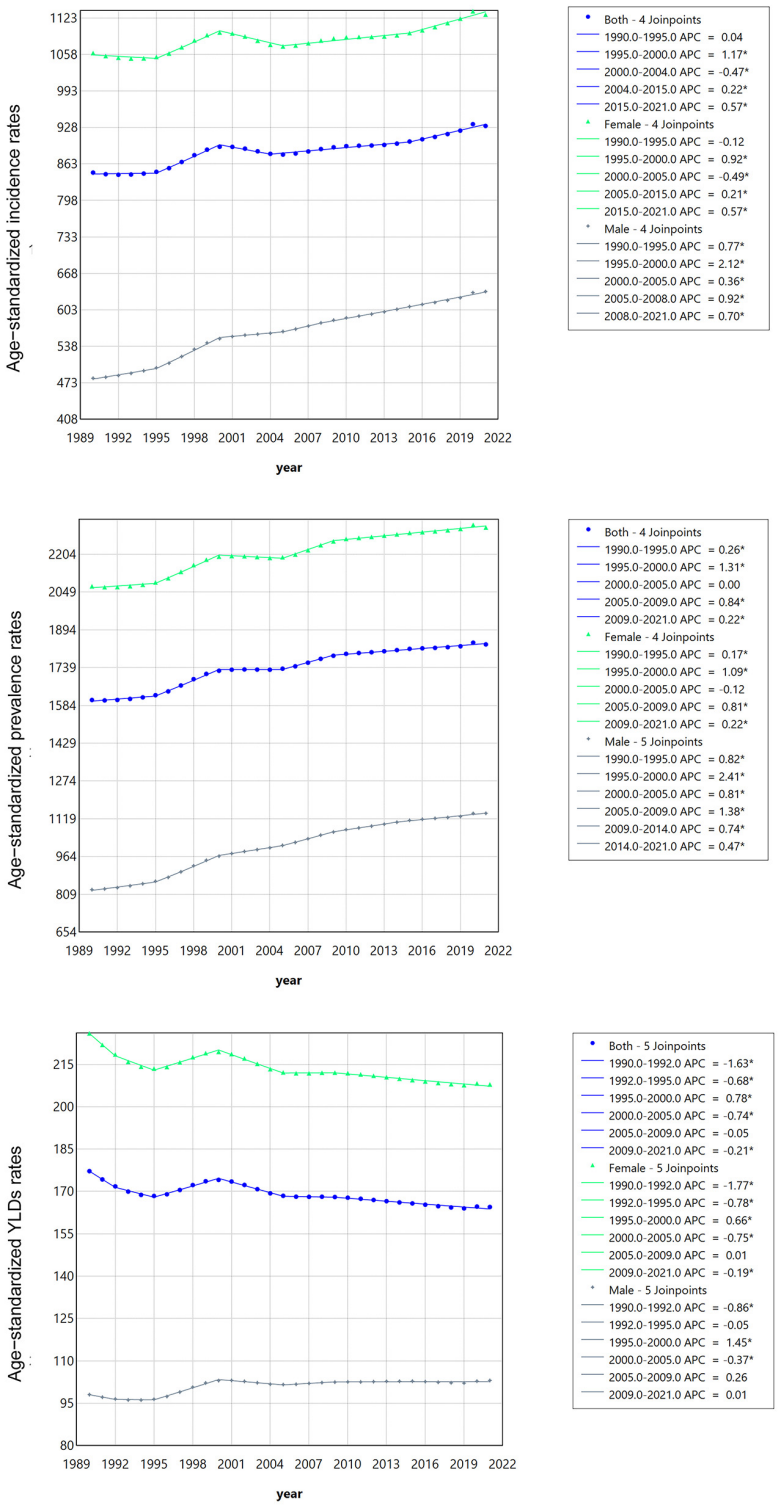
The jointpoint regression analysis of hip fractures attributable to falls in older adults from 1990 to 2021.

Joinpoint regression analysis was used to evaluate the segmented trends of ASIR, ASPR, and ASYR of HFAF in older adults (Figure 1, Supplementary Table S2). Although ASIR [average annual percent change (AAPC) = 0.322%, 95% CI: 0.254, 0.389] and ASPR (AAPC = 0.445%, 95% CI: 0.400%, 0.489%) showed increasing trends, ASYR (AAPC = −0.253%, 95% CI: −0.337%, −0.169%) showed a decreasing trend. These data indicate that over the past three decades, ASIR and ASPR of HFAF in older adults have increased significantly, reflecting a continuously worsening global health burden. However, the decreasing ASYR suggests that medical advancements over the past three decades have reduced disease-related disability duration.

### 3.2 Diverse disease burden of HFAF in older adults across regions and countries

**Figure 2:**
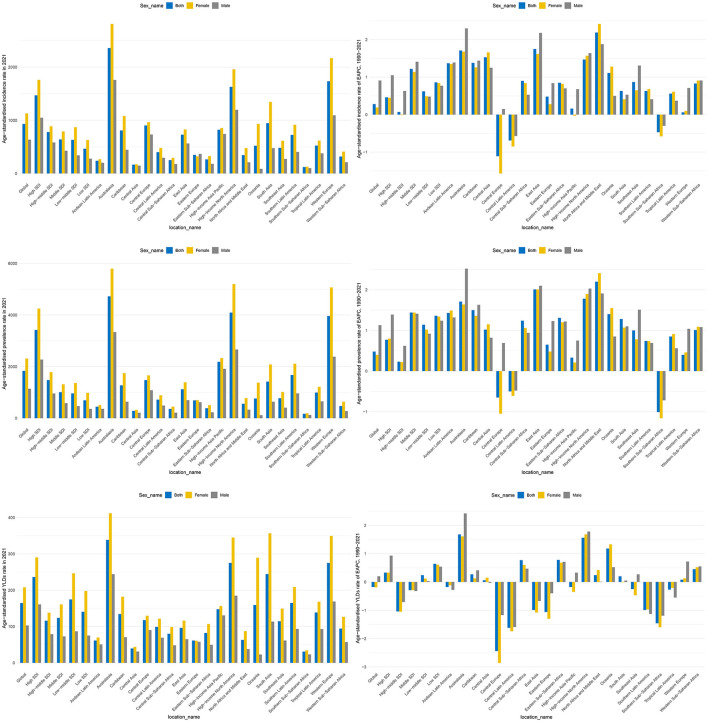
The ASIR, ASPR, and ASYR of hip fractures attributable to falls in older adults globally, across the 5 SDI regions, and 21 GBD regions in 2021, as well as the EAPC of ASIR, ASPR, and ASYR from 1990 to 2021. ASIR, Age-standardized incidence rate; ASPR, Age-standardized prevalence rate; ASYR, Age-standardized years lived with disability rate; EAPC, Estimated annual percentage change.

At the SDI regional level, 2021 data showed a striking gradient in disease burden: High SDI regions had the highest ASIR (1,469.17 per 100,000; 95% UI: 1,013.83, 2,039.11), which was 3.2 times higher than that of Low SDI regions (464.74 per 100,000; 95% UI: 293.86, 693.83). A similar pattern was observed for ASPR: High SDI regions (3,421.55 per 100,000; 95% UI: 2,759.5, 4,210.12) had a 4.9-fold higher rate than Low SDI regions (693.26 per 100,000; 95% UI: 526.32, 905.06). High SDI regions had the highest ASYR [236.3 (95% UI: 154.2, 339.97)] of HFAF in older adults. From 1990 to 2021, the Middle SDI region exhibited the fastest growth in ASIR (EAPC = 1.22, 95% CI: 1.02, 1.42) and ASPR (EAPC = 1.44, 95% CI: 1.26, 1.61), whereas High-middle SDI regions had the largest ASYR decrease (EAPC = −1.04, 95% CI: −1.15, −0.93; Figure 2, Table 1).

At the GBD regional level, in 2021, Australasia had the highest ASIR [2,358.9 (95% UI: 1,676.77, 3,151.75)], ASPR [4,725.35 (95% UI: 3,901.08, 5,685.83)], and ASYR [338.62 (95% UI: 225.18, 484.8)] of HFAF in older adults. From 1990 to 2021, ASIR showed the largest increase in North Africa and the Middle East [EAPC = 2.19 (95% CI: 2.05, 2.34)] and the largest decrease in Central Europe [EAPC = −1.11 (95% CI: −1.2, −1.03)]. ASPR showed the largest increase in North Africa and the Middle East [EAPC = 2.2 (95% CI: 2.04, 2.37)] and the largest decrease in Southern Sub-Saharan Africa [EAPC = −1.01 (95% CI: −1.2, −0.83)]. ASYR showed the largest increase in Australasia [EAPC = 1.68 (95% CI: 1.51, 1.84)] and the largest decrease in Central Europe [EAPC = −2.44 (95% CI: −2.55, −2.33)] (Figure 2, Table 1).

**Figure 3:**
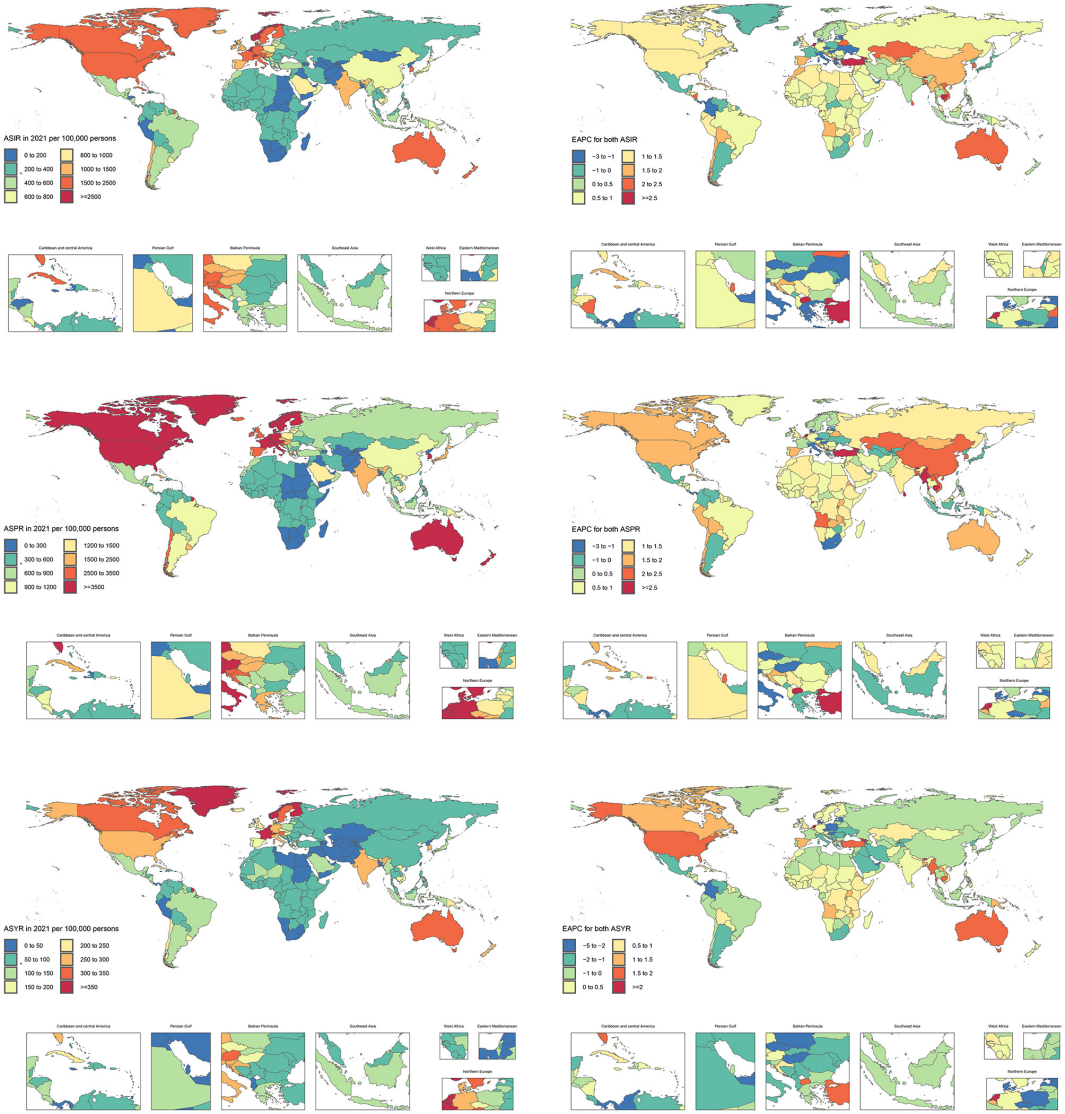
The ASIR, ASPR, and ASYR of hip fractures attributable to falls in older adults in 204 countries in 2021, as well as the EAPC of ASIR, ASPR, and ASYR from 1990 to 2021. ASIR, Age-standardized incidence rate; ASPR, Age-standardized prevalence rate; ASYR, Age-standardized years lived with disability rate; EAPC, Estimated annual percentage change.

At the country level, in 2021, Andorra had the highest ASIR [3,379.92 (95% UI: 2,334.7, 4,603.8)] and ASPR [7,136.9 (95% UI: 5,919.09, 8,514.99)], while Greenland had the highest ASYR [545.42 (95% UI: 368.68, 760.05)]. ASIR showed the largest increase in Turkey [EAPC = 4.4 (95% CI: 4.02, 4.78)] and the largest decrease in Czechia [EAPC = −2.77 (95% CI: −2.94, −2.59)]. ASPR showed the largest increase in Turkey [EAPC = 4.24 (95% CI: 3.88, 4.61)] and the largest decrease in Hungary (EAPC = −2.15 (95% CI: −2.35, −1.96)]. ASYR showed the largest increase in the Netherlands [EAPC = 2.55 (95% CI: 1.71, 3.4)] and the largest decrease in Hungary [EAPC = −4.04 (95% CI: −4.25, −3.82)] (Figure 3, Supplementary Tables S3–S5).

### 3.3 Disease burden of HFAF in older adults by gender

**Figure 4:**
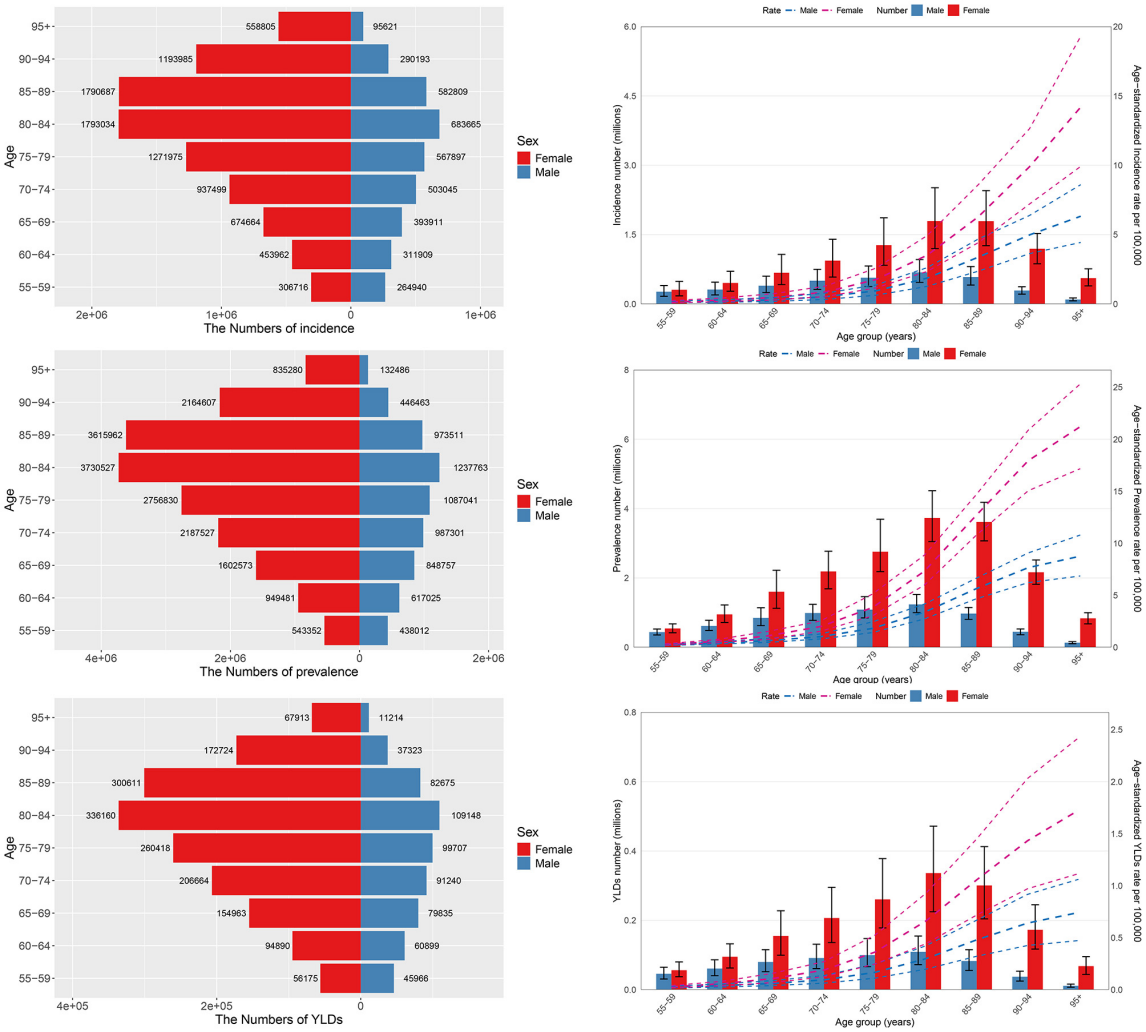
The prevalence, incidence, and YLDs of hip fractures attributable to falls in older adults, stratified by age and gender. YLDs, Years lived with disability.

Significant differences were observed in the age and gender distribution of HFAF in older adults globally. Age group analysis revealed that the 80–84 years group had the highest number of prevalent cases, incident cases, and YLDs. Gender-based analysis showed that females had higher numbers of prevalent cases, incident cases, and YLDs than males across all age groups. Over the past 30 years, the number of prevalent cases, incident cases, and YLDs has continued to increase in both females and males, with females consistently having higher numbers than males in any given year (Figure 4). This disparity is primarily driven by postmenopausal osteoporosis and an accelerated decline in muscle strength among women.

#### 3.3.1 The global disease burden of HFAF in older women and older men

From 1990 to 2021, the global disease burden of HFAF in older females showed an increasing trend. ASIR increased from 1,060.70 cases per 100,000 population (95% UI: 699.29, 1,533.1) to 1,129.07 cases per 100,000 population (95% UI: 754.2, 1,607.57), with an EAPC of 0.19 (95% CI: 0.15, 0.23). ASPR increased from 2,072.51 cases per 100,000 population (95% UI: 1,644.05, 2,604.8) to 2,313.46 cases per 100,000 population (95% UI: 1,470.71, 2,274.39). ASYR decreased from 255.96 cases per 100,000 population (95% UI: 150.65, 320.58) to 207.90 cases per 100,000 population (95% CI: 139.02, 294.47), with an EAPC of −0.19 (95% CI: −0.23, −0.16) (Table 1). Joinpoint analysis showed increasing trends in ASIR (AAPC = 0.227%, 95% CI: 0.16, 0.293) and ASPR (AAPC = 0.374%, 95% CI: 0.33, 0.418) and a decreasing trend in ASYR (AAPC = −0.278%, 95% CI: −0.347, −0.209) among older females. The increase in ASIR and ASPR, coupled with the decrease in ASYR, indicates that while the disease burden of HFAF in older females is increasing, current treatment methods are effective, and efforts should continue to advance hip fracture treatments to mitigate the increasing burden (Figure 1, Supplementary Table S2).

From 1990 to 2021, the global disease burden of HFAF in older males showed an increasing trend. ASIR increased from 481.23 cases per 100,000 population (95% UI: 313.67, 704.35) to 635.76 cases per 100,000 population (95% UI: 426.75, 904.48), with an EAPC of 0.91 (95% CI: 0.84, 0.97). ASPR increased from 828.23 cases per 100,000 population (95% UI: 647.59, 1,060.12) to 1,141.48 cases per 100,000 population (95% CI: 908.43, 1,426.3), with an EAPC of 1.13 (95% CI: 1.05, 1.22). ASYR increased from 98.06 cases per 100,000 population (95% UI: 64.81, 139.81) to 103.08 cases per 100,000 population (95% CI: 68.38, 147.34), with an EAPC of 0.20 (95% CI: 0.14, 0.26) (Table 1). Males showed increasing trends in ASIR (AAPC = 0.907%, 95% CI: 0.835, 0.98), ASPR (AAPC = 1.054%, 95% CI: 0.994, 1.113), and ASYR (AAPC = 0.149%, 95% CI: 0.054, 0.243) (Figure 1, Supplementary Table S2). The increases in ASIR, ASPR, and ASYR indicate that the disease burden of HFAF in older males is growing, with current treatment and care methods being ineffective.

#### 3.3.2 The regional disease burden of HFAF in older women and older men

At the SDI regional level, among older females, High SDI regions had the highest ASIR [1,759.75 (95% UI: 1,206.66, 2,451.1)], ASPR [4,247.6 (95% UI: 3,419.95, 5,226.59)], and ASYR [290.34 (95% UI: 189.1, 417.21)]. ASIR showed the largest increase in Middle SDI regions [EAPC = 1.14 (95% CI: 0.95, 1.34)] and the largest decrease in High-Middle SDI regions [EAPC = −0.01 (95% CI: 20.11, 0.08)]. ASPR showed increasing trends across all SDI regions, with the largest increase in Middle SDI regions [EAPC = 1.44 (95% CI: 1.27, 1.6)]. ASYR showed the largest increase in Low-middle SDI regions [EAPC = 0.61 (95% CI: 0.52, 0.7)] and the largest decrease in High-middle SDI regions [EAPC = −1.05 (95% CI: −1.16, −0.95)] (Figure 2, Supplementary Tables S3–S5).

Among older males, High SDI regions had the highest ASIR [1,047.62 (95% UI: 721.94, 1,462.29)], ASPR [2,272.5 (95% UI: 1,807.67, 2,817.1)], and ASYR [160.8 (95% UI: 104.46, 232.36)]. ASIR showed increasing trends across all SDI regions, with the largest increase in Middle SDI regions [EAPC = 1.41 (95% CI: 1.2, 1.62)]. ASPR also showed increasing trends across all SDI regions, with the largest increase in Middle SDI regions [EAPC = 1.41 (95% CI: 1.22, 1.59)]. ASYR showed the largest increase in High SDI regions [EAPC = 0.93 (95% CI: 0.83, 1.03)] and the largest decrease in High-middle SDI regions [EAPC = −0.71 (95% CI: −0.83, −0.59)] (Figure 2, Supplementary Tables S3–S5).

At the GBD regional level, among older females, Australasia had the highest ASIR [2,812.56 (95% UI: 1,963.16, 3,808.39)], ASPR [5,797.13 (95% UI: 4,768.78, 6,974.74)], and ASYR [411.66 (95% UI: 272.09, 587.86)]. ASIR showed the largest increase in North Africa and the Middle East [EAPC = 2.42 (95% CI: 2.26, 2.58)] and the largest decrease in Central Europe [EAPC = −1.57 (95% CI: −1.67, −1.47)]. ASPR showed the largest increase in North Africa and the Middle East [EAPC = 2.41 (95% CI: 2.23, 2.58)] and the largest decrease in Southern Sub-Saharan Africa [EAPC = −1.16 (95% CI: −1.37, −0.95)]. ASYR showed the largest increase in Australasia [EAPC = 1.68 (95% CI: 1.53, 1.83)] and the largest decrease in Central Europe [EAPC = −2.89 (95% CI: −2.99, −2.72)] (Figure 2, Supplementary Tables S3–S5).

Among older males, Australasia had the highest ASIR [1,758.99 (95% UI: 1,241.18, 2,363.46)], ASPR [3,339.09 (95% UI: 2,716.67, 4,061.08)], and ASYR [244.08 (95% UI: 161.23, 348.53)]. ASIR showed the largest increase in Australasia [EAPC = 2.30 (95% CI: 2.07, 2.53)] and the largest decrease in Central Latin America [EAPC = −0.57 (95% CI: −0.66, −0.47)]. ASPR showed the largest increase in Australasia [EAPC = 2.52 (95% CI: 2.27, 2.78)] and the largest decrease in Southern Sub-Saharan Africa [EAPC = −0.72 (95% CI: −0.86, −0.58)]. ASYR showed the largest increase in Australasia [EAPC = 2.42 (95% CI: 2.17, 2.66)] and the largest decrease in Central Europe [EAPC = −1.59 (95% CI: −1.72, −1.46)] (Figure 2, Supplementary Tables S3–S5).

#### 3.3.3 The disease burden of HFAF in older women and older men across 204 countries and territories

At the country level, among older females, Andorra had the highest ASIR [5,166.82 (95% UI: 3,522.98, 7,125.08)], ASPR [10,988.09 (95% UI: 9,096, 13,132.92)], and ASYR [774.15 (95% UI: 513.46, 1,108.34)]. ASIR showed the largest increase in Turkey [EAPC = 4.6 (95% CI: 4.23, 4.98)] and the largest decrease in Czechia [EAPC = −3.24 (95% CI: −3.44, −3.05)]. ASPR showed the largest increase in Turkey [EAPC = 4.41 (95% CI: 4.08, 4.75)] and the largest decrease in Hungary [EAPC = −2.49 (95% CI: −2.69, −2.29)]. ASYR showed the largest increase in the Netherlands [EAPC = 2.69 (95% CI: 1.81, 3.58)] and the largest decrease in Hungary [EAPC = −4.41 (95% CI: −4.63, −4.19)] (Supplementary Figures S2, S3; Supplementary Tables S3–S5).

Among older males, Norway had the highest ASIR [1,835.45 (95% UI: 1,181.69, 2,670.6)], Finland had the highest ASPR [4,153.63 (95% UI: 3,367.32, 5,024.16)], and Greenland had the highest ASYR [352.96 (95% UI: 235.43, 495.37)]. ASIR showed the largest increase in Turkey [EAPC = 4.15 (95% CI: 3.8, 4.5)] and the largest decrease in Armenia [EAPC = −1.85 (95% CI: −2.13, −1.57)]. ASPR showed the largest increase in Turkey [EAPC = 4.0 (95% CI: 3.73, 4.28)] and the largest decrease in Armenia [EAPC = −1.82 (95% CI: −2.16, −1.48)]. ASYR showed the largest increase in the Netherlands [EAPC = 3.12 (95% CI: 2.32, 3.93)] and the largest decrease in Armenia [EAPC = −3.12 (95% CI: −3.43, −2.82)] (Supplementary Figures S2, S3; Supplementary Tables S3–S5).

### 3.4 Correlation between SDI and disease burden

**Figure 5:**
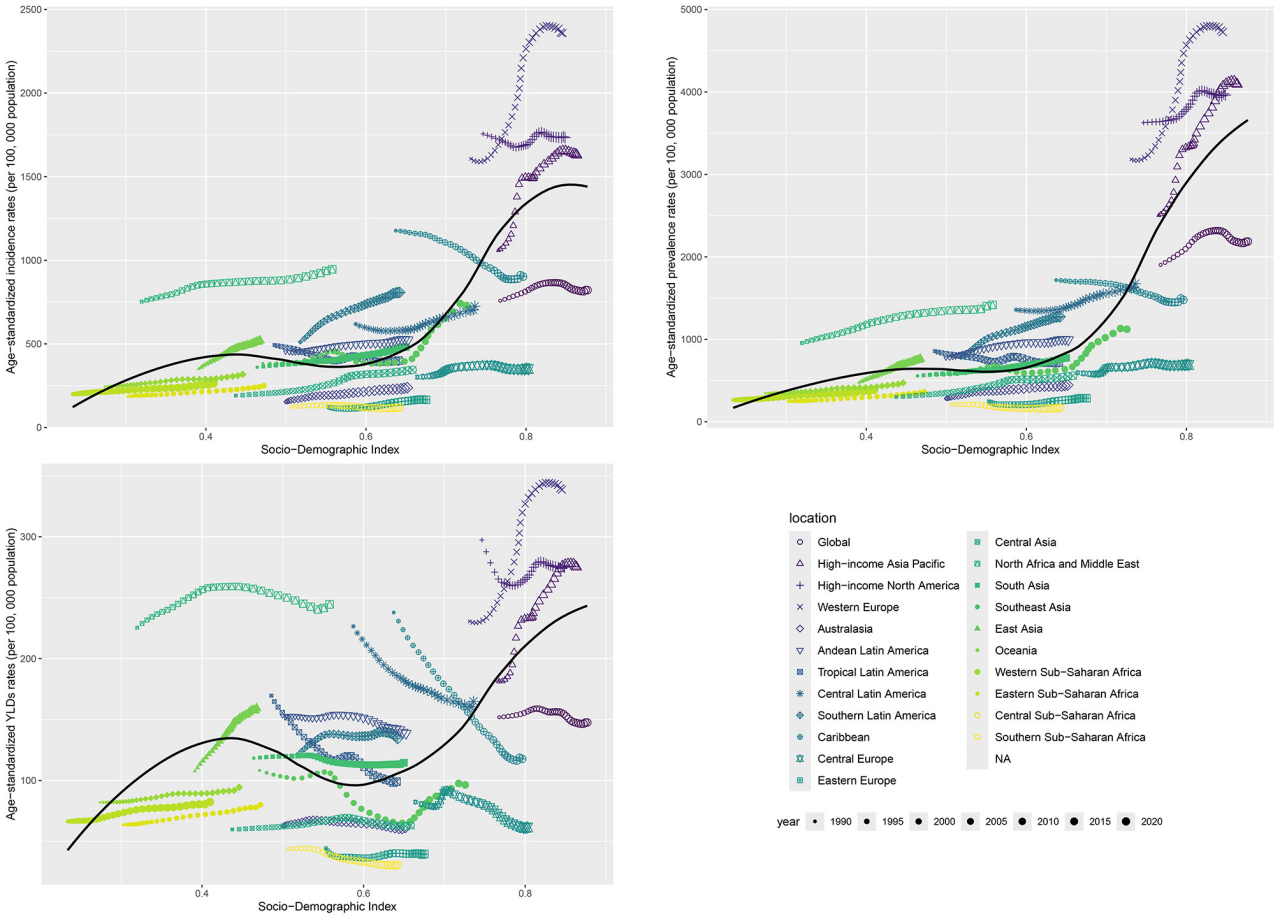
The ASIR, ASPR, and ASYR of hip fractures attributable to falls in older adults in 21 GBD regions by the SDI. ASIR, Age-standardized incidence rate; ASPR, Age-standardized prevalence rate; ASYR, Age-standardized years lived with disability rate; SDI, Socio-demographic index; GBD, global burden of disease.

**Figure 6:**
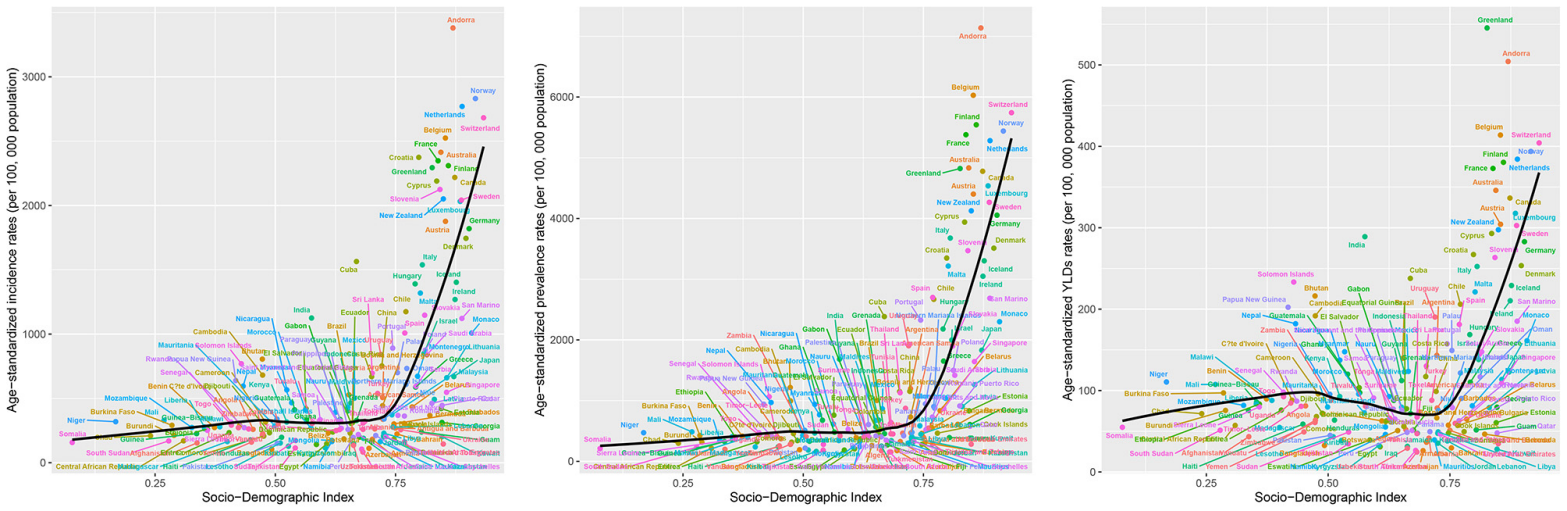
The ASIR, ASPR, and ASYR of hip fractures attributable to falls in older adults in 204 countries by the SDI. ASIR, Age-standardized incidence rate; ASPR, Age-standardized prevalence rate; ASYR, Age-standardized years lived with disability rate; SDI, Socio-demographic index.

Over the past three decades, the overall disease burden of HFAF in older adults increased with economic development across the 21 GBD regions. In general, ASIR, ASPR, and ASYR were positively correlated with SDI. Specifically, ASIR and ASPR remained stable when SDI was between 0 and 0.65 but increased continuously with SDI once SDI exceeded 0.65. ASYR showed a non-linear S-shaped relationship with SDI: it decreased with increasing SDI when SDI was below 0.4 or above 0.6, but was positively correlated with SDI when SDI was between 0.4 and 0.6 (Figure 5). Among the 204 countries, ASIR, ASPR, and ASYR remained stable when SDI was between 0 and 0.7 but increased continuously with SDI once SDI exceeded 0.7 (Figure 6).

### 3.5 Age-period-cohort analysis

**Figure 7:**
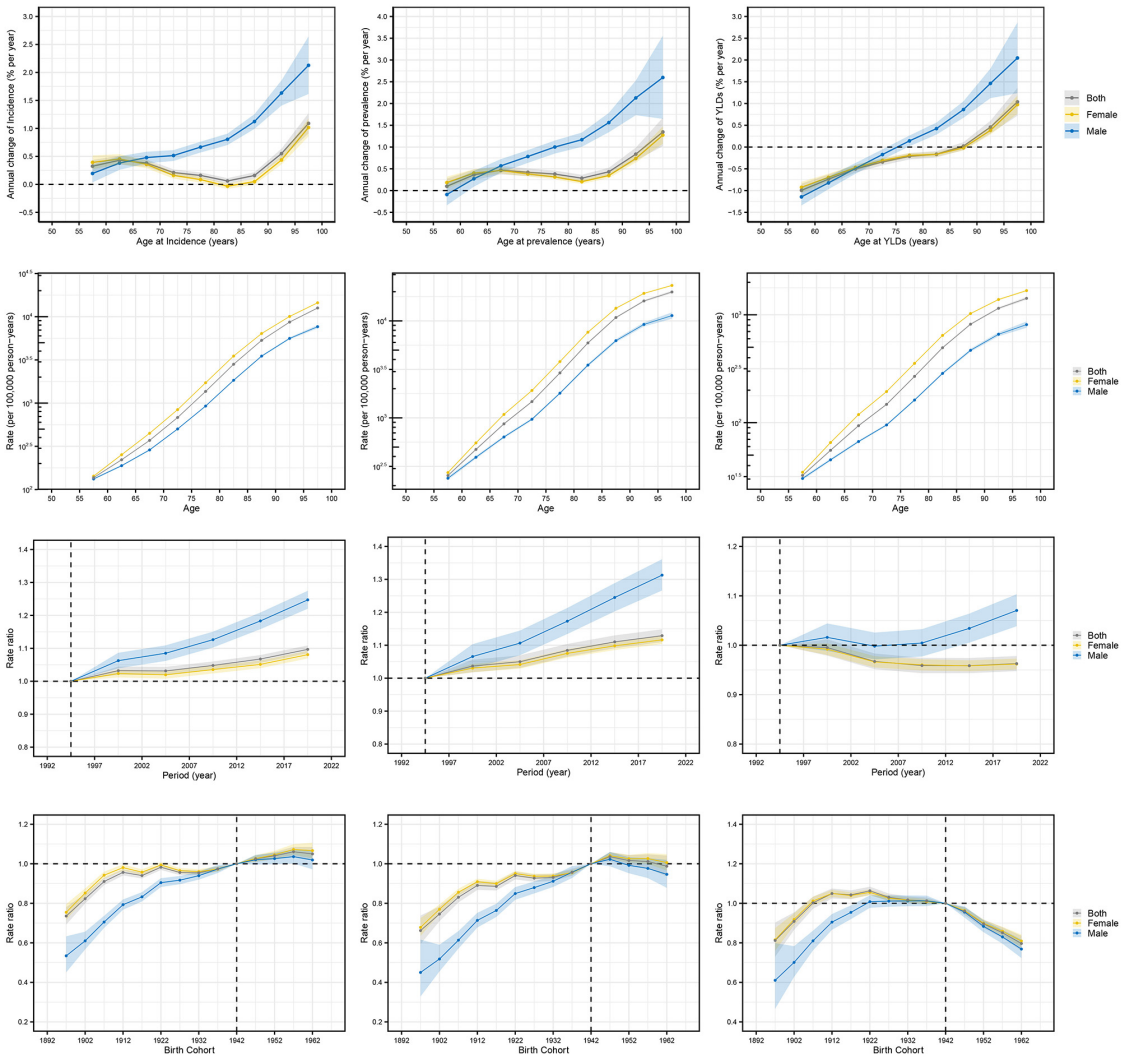
The impacts of age effect, period effect, and cohort effect on the prevalence, incidence, and YLDs of hip fractures attributable to falls in older adults. YLDs, Years lived with disability.

Local drift, calculated using the age-period-cohort model, represents the AAPC of prevalence, incidence, and YLDs across age groups. Incidence and prevalence increased continuously in all age groups, with the most significant increase in the 95+ years age group (local drift coefficients: 1.089 for incidence and 1.347 for prevalence). For HFAF, older males (incidence = 2.217, prevalence = 2.598) and older females (incidence = 1.016, prevalence = 1.274) also showed the most significant increases in the 95+ years age group. YLDs showed increasing trends in the 85–89 years, 90–94 years, and 95+ years age groups, with the largest local drift coefficient (1.038) in the 95+ years group. YLDs showed decreasing trends in other age groups, with the largest local drift coefficient (−0.994) in the 55–59 years group. Among older males, YLDs of HFAF showed increasing trends in the 75–79 years, 80–84 years, 85–89 years, 90–94 years, and 95+ years age groups, with the largest local drift coefficient (1.237) in the 95+ years group; YLDs showed decreasing trends in other age groups, with the largest local drift coefficient (−1.145) in the 55–59 years group. Among older females, YLDs showed increasing trends in the 90–94 years and 95+ years age groups, with the largest local drift coefficient (0.975) in the 95+ years group; YLDs showed decreasing trends in other age groups, with the largest local drift coefficient (−0.925) in the 55–59 years group (Figure 7, Supplementary Tables S6–S8).

Age effects revealed significant differences in prevalence across age groups: the incidence, prevalence, and YLDs of HFAF in older adults increased with age, indicating a higher risk in older individuals. Period effect analysis showed a gradual increase in prevalence and incidence from 1990 to 2021. YLDs of HFAF in older males fluctuated significantly, showing a trend of initial increase, followed by decrease, and then re-increase, while YLDs in older females decreased continuously. Overall, fall-related YLDs in older adults decreased over time. Cohort effect analysis indicated that later-born cohorts had a higher relative risk of prevalence and incidence of HFAF in older adults. However, the cohort effect on YLDs showed a trend of initial increase followed by decrease, which may be influenced by medical policies for hip fracture treatment and care in different periods. Later-born cohorts had a lower relative risk of YLDs. These findings highlight the complex interactions of age, period, and birth cohort in influencing the prevalence, incidence, and YLDs of HFAF in older adults (Figure 7, Supplementary Tables S6–S8).

### 3.6 Decomposition analysis

**Figure 8:**
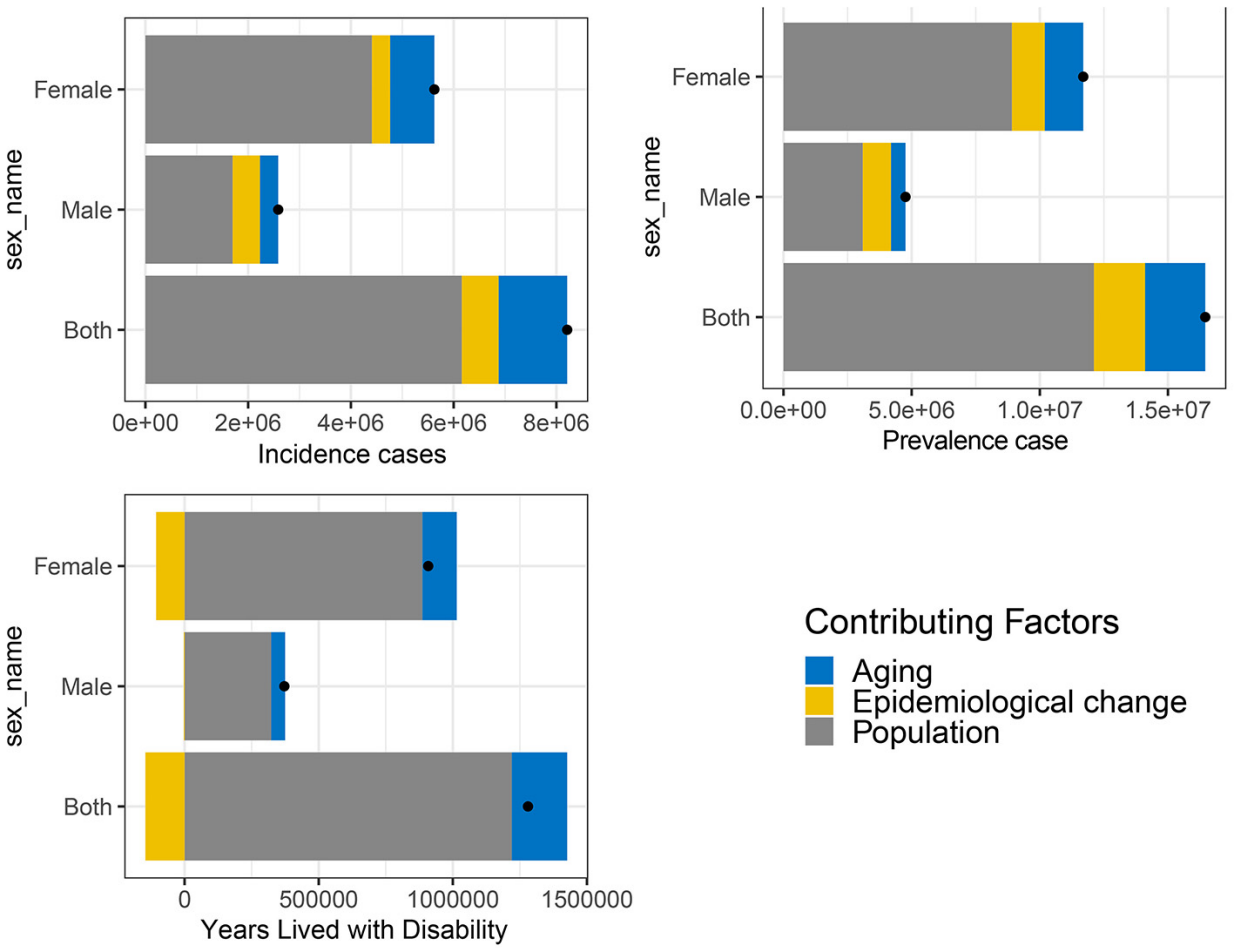
Decomposition analysis of changes in the prevalence, incidence, and YLDs of hip fractures attributable to falls in older adults globally from 1990 to 2021.YLDs, Years lived with disability.

Decomposition analysis was performed to further explore the impacts of aging, population growth, and epidemiological changes on prevalence, incidence, and YLDs. Globally, population growth was the primary factor influencing prevalence, incidence, and YLDs from 1990 to 2021. Over the past 30 years, incidence increased by 8,209,555.97, with population growth contributing 6,159,109.56 (75.02%), aging contributing 1,335,309.80 (16.27%), and epidemiological changes contributing only 715,136.61 (8.71%). Prevalence increased by 16,454,176.50, with population growth contributing 12,111,087.83 (73.61%), aging contributing 2,346,846.37 (14.26%), and epidemiological changes contributing 1,996,242.30 (12.13%). YLDs increased by 1,280,180.85, with population growth contributing 1,219,855.27 (95.29%), aging contributing 206,419.05 (16.12%), and epidemiological changes contributing −146,093.47 (−11.41%) (Figure 8, Supplementary Table S9). When stratified by gender, population growth was also the primary driver of the increase in prevalence, incidence, and YLDs of HFAF in both older males and females (Figure 8, Supplementary Table S9). By gender, population growth remained the top driver, but aging had a larger impact on females than males, reflecting the higher proportion of older females globally.

### 3.7 Cross-country inequalities analysis

**Figure 9:**
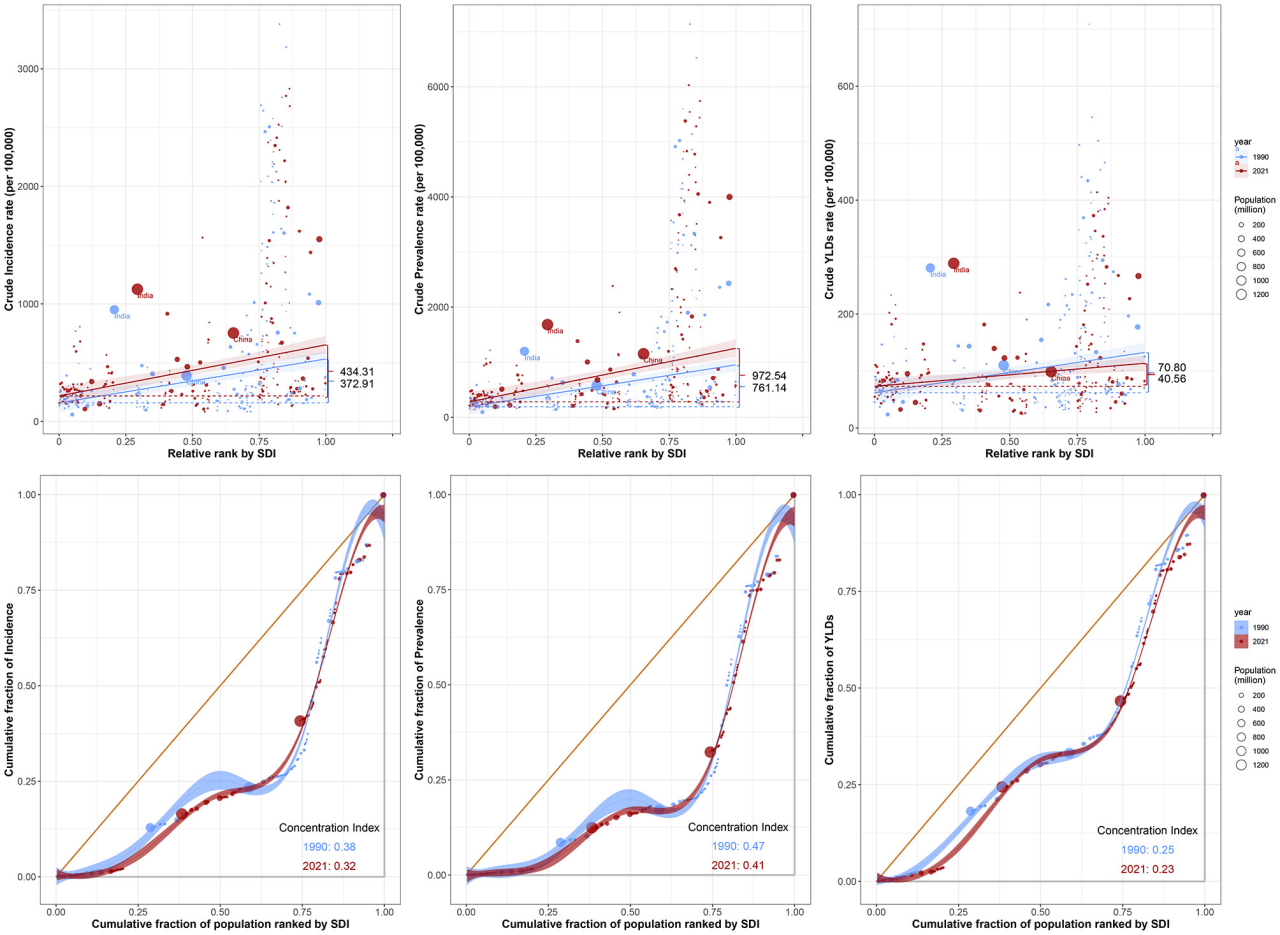
Cross-country inequality analysis of the ASIR, ASPR, and ASYR of hip fractures attributable to falls in older adults in 1990 and 2021. ASIR, Age-standardized incidence rate; ASPR, Age-standardized prevalence rate; ASYR, Age-standardized years lived with disability rate.

Analysis of 204 countries and regions revealed significant differences in the burden of HFAF in older adults associated with SDI, with the burden concentrated primarily in high-SDI regions. Over time, inequalities in ASIR and ASPR increased, while inequalities in ASYR decreased (Figure 9). The slope index of absolute inequality showed that the ASIR difference between the lowest and highest SDI countries/regions increased from 372.91 in 1990 to 434.31 in 2021. Similarly, the ASPR difference between the lowest and highest SDI countries/regions increased from 761.14 in 1990 to 972.54 in 2021. However, the ASYR difference between the lowest and highest SDI countries/regions decreased from 70.80 in 1990 to 40.56 in 2021. In 1990, the concentration indices of health inequality for ASIR, ASPR, and ASYR were 0.38, 0.47, and 0.25, respectively; these values changed to 0.32, 0.41, and 0.23 in 2021. These results indicate that the burden of HFAF in older adults remains concentrated in high-income regions. Although the absolute inequality gap in health outcomes between different socioeconomic groups has widened, the reduced gap in ASYR suggests that current hip fracture treatments are effective (Figure 9).

### 3.8 Frontier analysis

**Figure 10:**
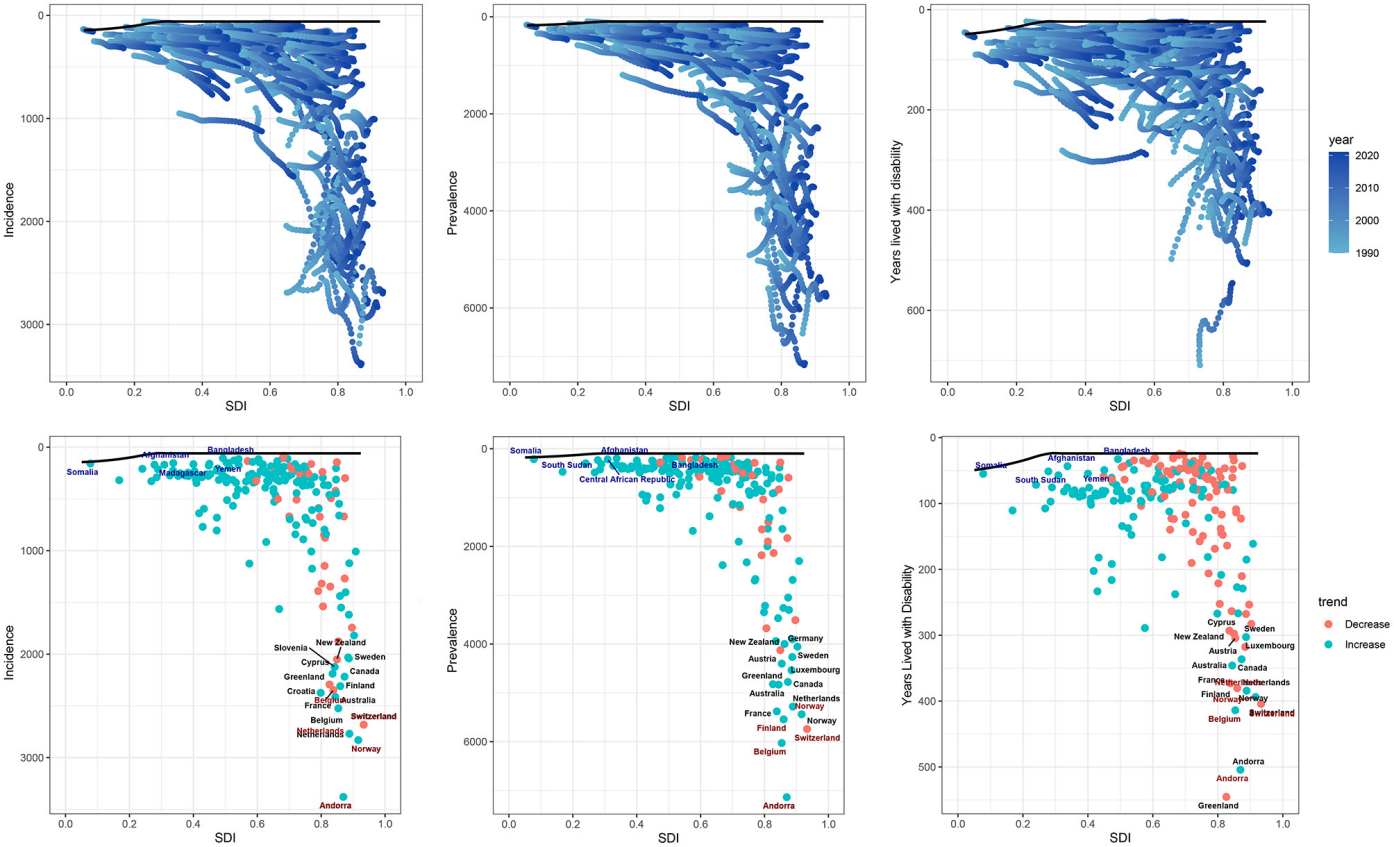
Frontier analysis for hip fractures attributable to falls in older adults in 204 countries in 2021. The optimal practice frontier is delimited by a solid black line, with countries/regions represented as points and the top 15 countries with the largest efficiency gaps labeled. Low-SDI regions and countries/regions with smaller efficiency gaps are labeled in blue font, while high-SDI countries/regions with efficiency gaps disproportionately large for their development levels are labeled in red font.

To better understand the potential for improving the burden of HFAF in older adults based on national SDI, frontier analysis was performed using ASIR, ASPR, ASYR, and SDI. For ASIR, the top 15 countries with the largest effective differences were Andorra, Norway, the Netherlands, Belgium, Switzerland, Australia, Croatia, France, Finland, Greenland, Canada, Cyprus, Slovenia, New Zealand, and Sweden. In low-SDI regions, the five countries with the smallest effective differences were Somalia, Afghanistan, Madagascar, Bangladesh, and Yemen. In high-SDI regions, the top five countries with the largest effective differences were Andorra, Norway, the Netherlands, Belgium, and Switzerland (Figure 10).

For ASPR, the top 15 countries with the largest effective differences were Andorra, Belgium, Switzerland, Finland, Norway, France, the Netherlands, Australia, Greenland, Canada, Luxembourg, Austria, Sweden, New Zealand, and Germany. In low-SDI regions, the five countries with the smallest effective differences were Somalia, Afghanistan, Bangladesh, the Central African Republic, and South Sudan. In high-SDI regions, the top five countries with the largest effective differences were Andorra, Belgium, Switzerland, Finland, and Norway (Figure 10).

For ASYR, the top 15 countries with the largest effective differences were Greenland, Andorra, Belgium, Switzerland, Norway, the Netherlands, Finland, France, Australia, Canada, Luxembourg, Austria, Sweden, New Zealand, and Cyprus. In low-SDI regions, the five countries with the smallest effective differences were Bangladesh, Somalia, Afghanistan, Yemen, and South Sudan. In high-SDI regions, the top five countries with the largest effective differences were Andorra, Belgium, Switzerland, Norway, and the Netherlands (Figure 10). These results indicate that despite favorable medical conditions in high-SDI regions, many countries still have room for optimization.

### 3.9 Forecasting analysis

**Figure 11:**
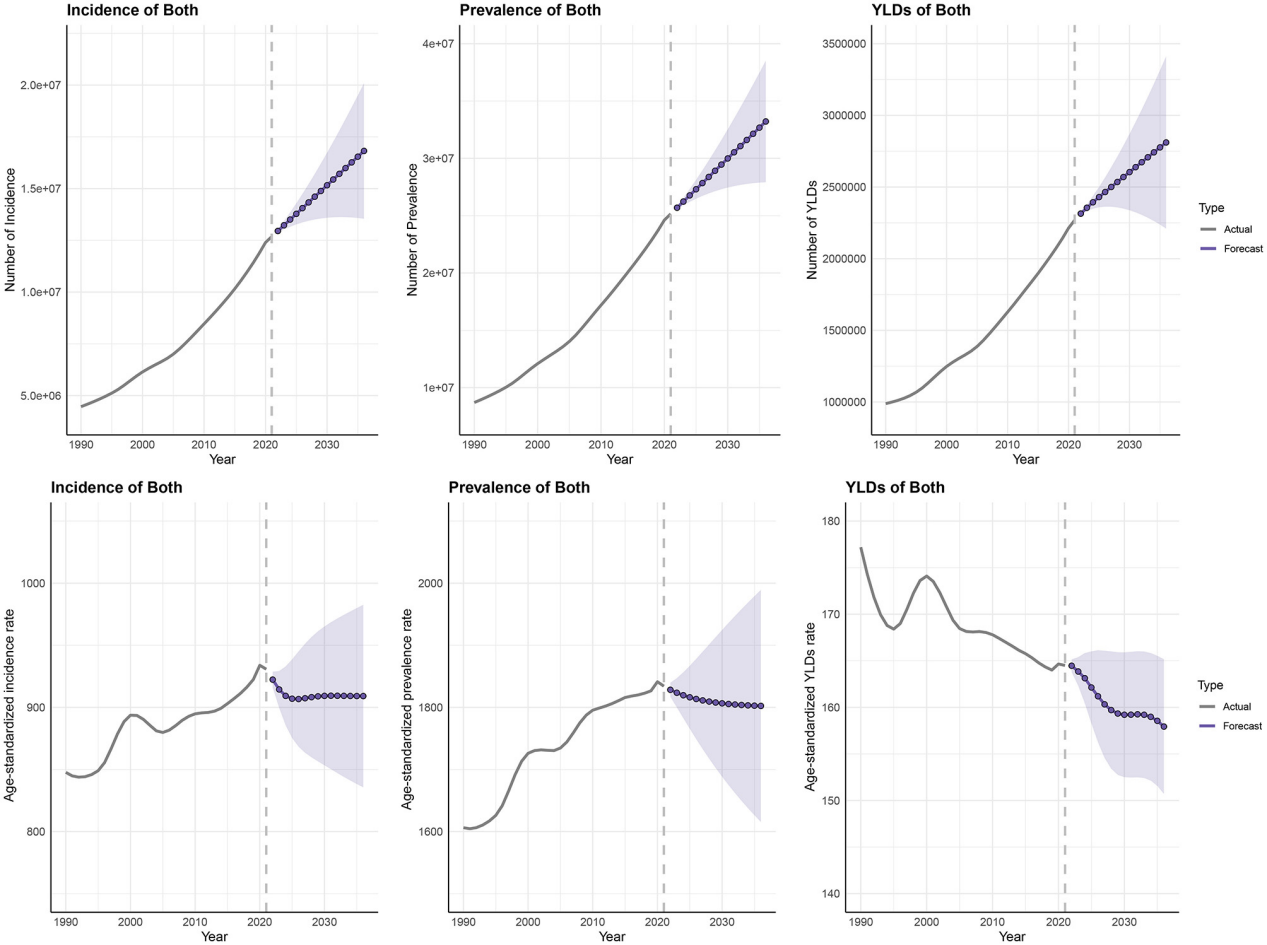
Prediction of the disease burden trend of hip fractures attributable to falls in older adults from 2022 to 2036.

In selecting the optimal model type and parameters, the Akaike Information Criterion (AIC) and Bayesian Information Criterion (BIC) were used to compare the goodness-of-fit of different models, with the model with the smallest criterion value selected. After model establishment, the Ljung-Box test for white noise was used to examine residual serial correlation (Supplementary Table S10). Projections indicate that by 2,036, with continued population growth, the number of incident cases [16,819,103 (95% UI: 13,544,912, 20,093,294)], prevalent cases [33,216,574 (95% UI: 27,917,941, 38,515,207)], and YLDs [2,810,808 (95% UI: 2,210,211, 3,411,405)] of HFAF in older adults will continue to increase. Benefiting from improved prevention policies and medical technologies, ASIR [909.078 (95% UI: 835.603, 982.553) per 100,000 population], ASPR [1,802.288 (95% UI: 1,615.45, 1,989.127) per 100,000 population], and ASYR [157.933 (95% UI: 150.701, 165.165) per 100,000 population] are projected to show a slight downward trend. The number of incident cases, prevalent cases, and YLDs are expected to continue increasing in both older females and males, with females remaining more heavily burdened than males (Figure 11, Supplementary Tables S11–S13, Supplementary Figures S4, S5).

## 4 Discussion

Using the latest data from the GBD 2021 database, the global and regional epidemiological characteristics of HFAF in older adults were systematically summarized, revealing an overall increasing trend in disease burden from 1990 to 2021. Through regional analysis, Socio-demographic Index (SDI) analysis, inequality analysis, and frontier analysis, significant disparities across geographic and SDI regions were identified: compared to 1990, regions with higher SDI levels in 2021 exhibited a heavier disease burden. Furthermore, health inequalities between high-SDI and low-SDI regions gradually widened. Decomposition analysis subsequently revealed that population growth was the primary driver of the increase in HFAF in older adults. Additionally, projections indicate that the number of prevalent cases, incident cases, and YLDs of HFAF in older adults will continue to rise over the next 15 years, while ASIR, ASPR, and ASYR will decline. This reflects the combined effects of substantial growth in the older adults and advancements in public health. These results align with an Italian study highlighting the dominant role of population aging in the hip fracture burden and validate the theory that “demographic transition is the core driver of older adults health issues” (32).

The distribution of HFAF in older adults is highly uneven across regions. In terms of regional distribution, ASIR, ASPR, and ASYR in high-SDI regions (e.g., Australasia, High SDI regions) were significantly higher than in low-SDI regions. A survey of six hospitals in China found that 74.5% of patients with HFAF were from economically developed urban areas, while only 25.5% were from less developed rural areas (33). This may be attributed to the higher proportion of older adults, longer life expectancy, more robust diagnostic systems, and more comprehensive medical records of falls in high-income regions. In contrast, low-SDI regions may underestimate the burden of various diseases due to underdiagnosis or incomplete data collection (34). Over the past 32 years, North Africa and the Middle East exhibited the largest increase in ASIR, while Central Europe showed a decreasing trend. The rapid upward trend in low- and middle-income countries is particularly concerning, as these regions often lack infrastructure for fracture prevention and management. Notably, the relationship between SDI and disease burden is non-linear: ASYR was positively correlated with SDI when SDI ranged from 0.4 to 0.6, but negatively correlated when SDI was below 0.4 or above 0.6. This may be because middle-SDI regions are experiencing a phase where “aging is accelerating faster than the growth of medical resources,” facing risks from high aging while lacking sufficient medical resources to respond. For example, Carlos-Rivera et al. (13) demonstrated in a Mexican study that hip fracture treatment costs in middle-income regions are rising rapidly due to uneven distribution of medical resources.

In terms of population characteristics, disease burden was higher in females than males across all age groups, consistent with female physiological characteristics such as increased osteoporosis risk due to declining postmenopausal estrogen levels and faster muscle strength deterioration (35, 36). Another interesting study found that among older females, Black and Asian females have a much lower fall risk compared to White females (37), suggesting that older white women may face a higher risk of HFAF. Decomposition analysis indicated that population growth is the primary factor driving increased disease burden, a finding that aligns with the population growth pressure reflected in the $80 billion annual fall-related healthcare expenditures in the United States (6). A Chinese study also confirmed that population growth is the main reason for the increase in the number of incident and prevalent hip fracture cases in China (38). As the world's most populous country, China has experienced population growth in recent decades. Additionally, with economic and medical development, life expectancy has extended, and China has entered an aging society, implying a heavier burden of HFAF in the future. Future public health policies must address both population expansion and aging. This suggests that health resource allocation requires a “dual assessment” mechanism, considering both changes in age-specific risks and anticipating shifts in medical resources driven by demographic structural changes.

Results from the age-period-cohort analysis showed that, in terms of age effects, the incidence, prevalence, and YLDs of HFAF in older adults increased significantly with age, consistent with the objective law of physiological deterioration in the older adults (35, 36). Period effect analysis revealed a continuous upward trend in prevalence and incidence over these 32 years, closely linked to accelerating global population aging and an increase in the absolute number of older adults. Period effects further reflect the combined impact of macro factors such as public health environments and medical practices. Later-born cohorts exhibited a higher relative risk of prevalence and incidence, which may be closely related to cross-generational lifestyle changes. Later-born cohorts were more likely to be exposed to environmental factors such as increased sedentary behavior and reduced physical activity during middle age, leading to insufficient muscle strength and bone mineral density reserves, making them more prone to HFAF in old age (39). Meanwhile, later-born cohorts have longer life expectancies, extending the exposure window to “fall-related risks for older adults” and further increasing disease burden at the birth cohort level. Notably, the cohort effect on YLDs showed a “first increase then decrease” trend, with later-born cohorts having a lower relative risk of YLDs, directly related to advancements in medical technology. In recent decades, the popularization of hip fracture surgeries (e.g., total hip arthroplasty), improved postoperative rehabilitation systems, and antibiotic-based infection control have significantly reduced disability duration. Even though later-born cohorts have a higher fracture incidence, disability-related life loss has decreased, reflecting the positive intervention of medical progress on disease outcomes (40, 41). In summary, the age-period-cohort analysis not only quantified the impact of each dimension on the burden of HFAF in older adults but also revealed dynamic relationships between physiological aging, social changes, and medical progress.

Health inequality analysis showed that disease burden is concentrated in high-SDI regions, with widening absolute inequalities in ASIR and ASPR, consistent with the global uneven distribution of health resources (42). Frontier analysis found that high-SDI countries such as Andorra and Norway had the largest effective differences, indicating room for improving prevention and control efficiency even in resource-rich regions, possibly due to insufficient coverage of preventive measures (e.g., home environment modifications) (43, 44). In contrast, low-SDI regions such as Somalia had smaller effective differences, which may be due to low baseline burdens or incomplete data collection. These findings highlight the urgency of allocating resources to high-burden countries and provide precise targets for international assistance. Forecasting analysis showed that the number of prevalent and incident cases and YLDs will continue to increase by 2036, while ASIR, ASPR, and ASYR will decrease, resulting from the combined effects of older adults growth and public health interventions. For example, Sing et al. (45) demonstrated that anti-osteoporotic drug use can reduce post-hip fracture mortality, thereby affecting age-standardized rates.

Previous studies have primarily relied on GBD 2019 data, which only covers up to 2019, and thus cannot reflect changes in disease burden following the COVID-19 pandemic in 2020–2021. Additionally, no studies have specifically analyzed the impact of falls on hip fractures in the older adults. This study is based on the latest GBD 2021 database, extending data analysis to 2021, and provides the most up-to-date and comprehensive global, regional, and national burden assessment of hip fractures caused by falls among the older adults to date. We specifically focused on the “fall” mechanism and older adults. Falls are the leading cause of hip fractures in the older adults, and this focus allows our findings to directly address the important public health issue of fall prevention in the older adults. The methods employed, such as age-period-cohort analysis and decomposition analysis, also offer methodological references for similar disease burden studies. At the public health policy level, clarifying the increasing trend of global disease burden and identifying population growth as the main driver provides data support for countries to formulate prevention and control strategies. The high disease burden and optimization potential in high-SDI regions suggest the need to strengthen preventive measures, while rapid growth in low-SDI regions highlights the importance of improving medical resource accessibility, aiding in the optimization of global medical resource allocation. The study also revealed population characteristics such as the 80–84 years peak age and higher female burden, providing a basis for precise clinical interventions (e.g., enhancing osteoporosis screening and fall prevention in older women). Additionally, clarifying the high mortality and economic costs of hip fractures emphasizes the importance of early surgery and comprehensive rehabilitation, which can promote improvements in clinical diagnosis and treatment standards.

Our study unveils several critical avenues for future research. First, the alarming rise in incidence rates within Middle SDI regions demands urgent, localized investigation. Future studies should employ individual-level data in these regions to identify modifiable risk factors that are driving this epidemic, as these factors may differ from those in high-income countries. Second, the concerning increase in ASYR among older males, in stark contrast to the decrease seen in females, points to a significant gender gap in post-fracture care. Prospective cohort studies are needed to compare recovery pathways, rehabilitation adherence, and comorbidity profiles between men and women to explain this disparity and develop gender-tailored intervention protocols. Finally, our frontier analysis identified specific high-SDI countries with a substantial “efficiency gap” between their current burden and the theoretical minimum achievable given their resources. Future implementation science research should focus on these countries to identify the health policy, clinical practice, or socioeconomic barriers that prevent them from achieving optimal outcomes, serving as a model for other nations.

However, this study has several limitations. First, GBD data relies on statistical models and literature synthesis rather than direct clinical diagnoses, which may result in underdiagnosis in low-SDI regions not being fully corrected. For example, some sub-Saharan African regions lack standardized diagnostic tools, potentially leading to severe underestimation of disease burden. Second, the impact of the COVID-19 pandemic on HFAF in older adults was not included in the analysis. The popularization of telemedicine during the pandemic may have temporarily improved diagnosis and treatment rates in high-SDI countries, but low-SDI regions may have experienced inaccurate 2020–2021 data due to low internet penetration. Finally, each statistical analysis model has inherent limitations: Joinpoint regression analysis relies on a preset number of inflection points to explain trends, while the ARIMA model assumes stable data trends and cannot account for future sudden factors such as pandemics, policy adjustments, or drug development.

## 5 Conclusion

This study highlights the intensifying global challenge of HFAF in older adults. With rising incidence, prevalence, and widening socioeconomic disparities, these factors are placing enormous pressure on medical resources and socioeconomic systems worldwide. Analyses show that the global burden of HFAF in older adults increased significantly from 1990 to 2021, with inequalities worsening at an accelerated rate in regions with weak health systems due to scattered medical resources and lagging diagnostic capabilities. Specifically, high-SDI regions need to strengthen preventive measures, while low-SDI regions need to improve medical accessibility—findings with important implications for optimizing global medical resource allocation and precise interventions. The gender disparity in HFAF in older adults underscores the need to strengthen osteoporosis prevention in postmenopausal older women. The reduction in YLDs indicates that current hip fracture treatments and care measures are effective. With medical technology development, the disease burden of HFAF in older adults can be reduced through both prevention and treatment. Overall, this study provides systematic epidemiological evidence in the field of HFAF in older adults, emphasizing the importance of global collaborative prevention and control. It is hoped that these findings will promote in-depth academic research and public health practice innovation, ultimately improving the health status of older populations.

## Data Availability

The original contributions presented in the study are included in the article/Supplementary material, further inquiries can be directed to the corresponding author.
