# Supplementary material for: Global, regional, and national burden of hip fractures attributable to falls in older adults: changes from 1990–2021 and 2036 projections
Source: Front Public Health. 2025 Sep 18;13:1674881. doi: 10.3389/fpubh.2025.1674881 (PMC12488404; doi:10.3389/fpubh.2025.1674881)
Supplement: Supplementary file 1 [file Table_1.docx]

**Global, regional, and national burden of hip fractures attributable to falls in older adults: changes from 1990-2021 and 2036 projections**

Binbin Zhang^1†^, Bin Dou^1†^, Kewen Li^1*^

1 Department of Orthopedics, Qinghai University Affiliated Hospital, The Clinical Medical College of Qinghai University, Xining, Qinghai, China.

Corresponding author: Kewen Li*

Email: qdfykwl@126.com

Binbin Zhang^1†^ and Bin Dou^1†^ contributed equally to this work.


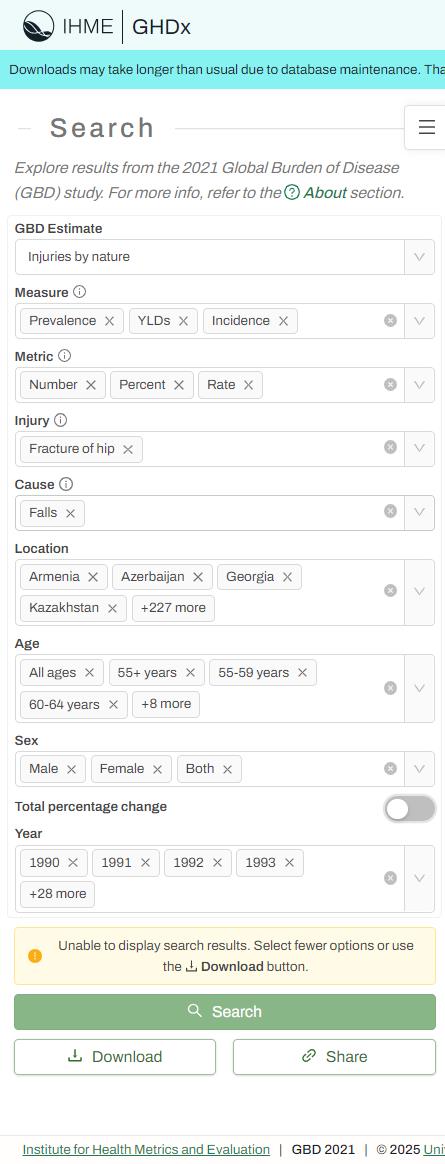


**Figure. S1** Data screening criteria for hip fractures attributable to falls in older adults

in the Global Burden of Disease Study 2021.


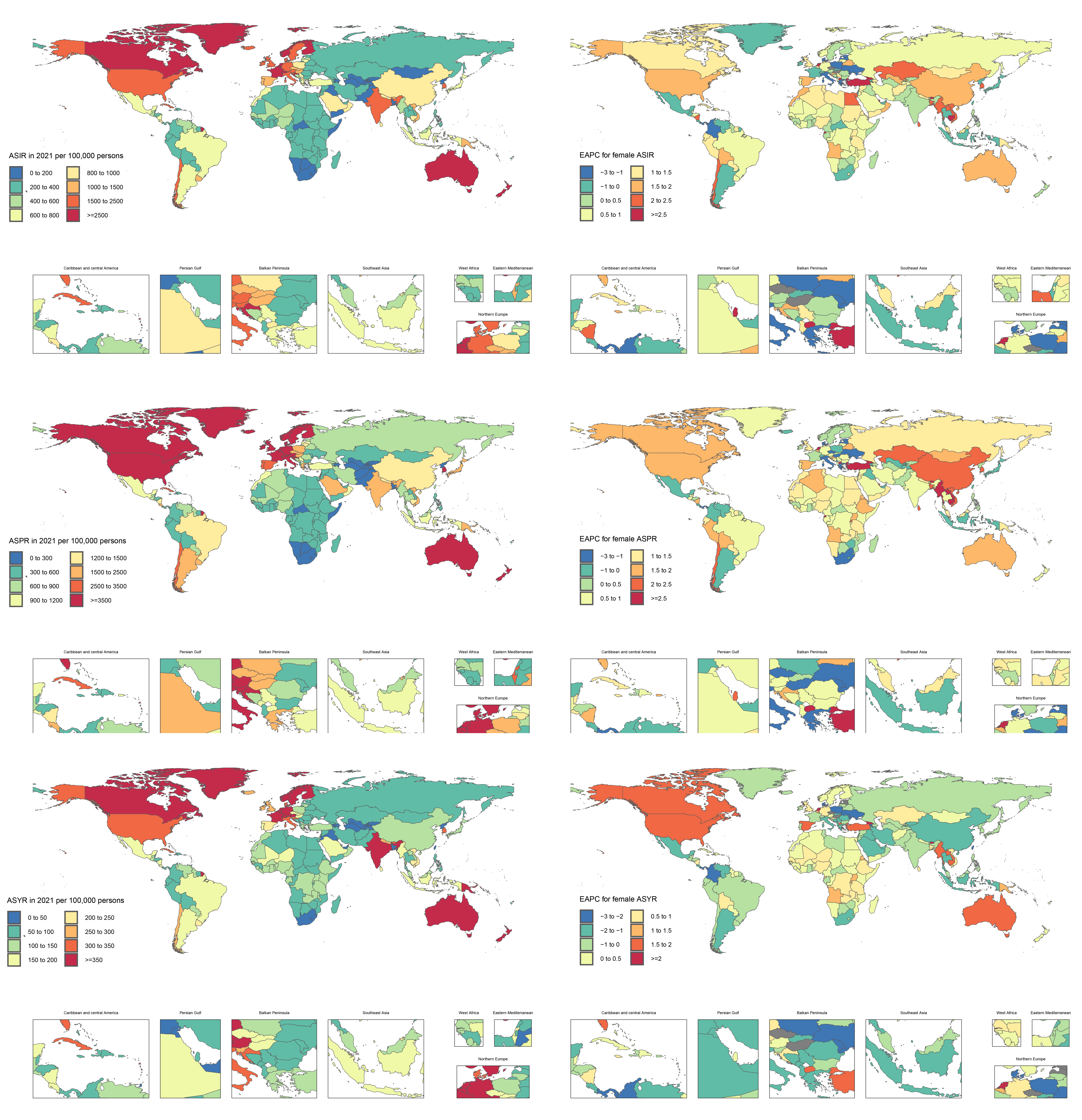


**Figure S2.** The ASIR, ASPR, and ASYR of hip fractures attributable to falls in older female in 204 countries in 2021, as well as the EAPC of ASIR, ASPR, and ASYR from 1990 to 2021.

Abbreviations: ASIR, Age-standardized incidence rate; ASPR, Age-standardized prevalence rate; ASYR, Age-standardized years lived with disability rate; EAPC, Estimated annual percentage change.


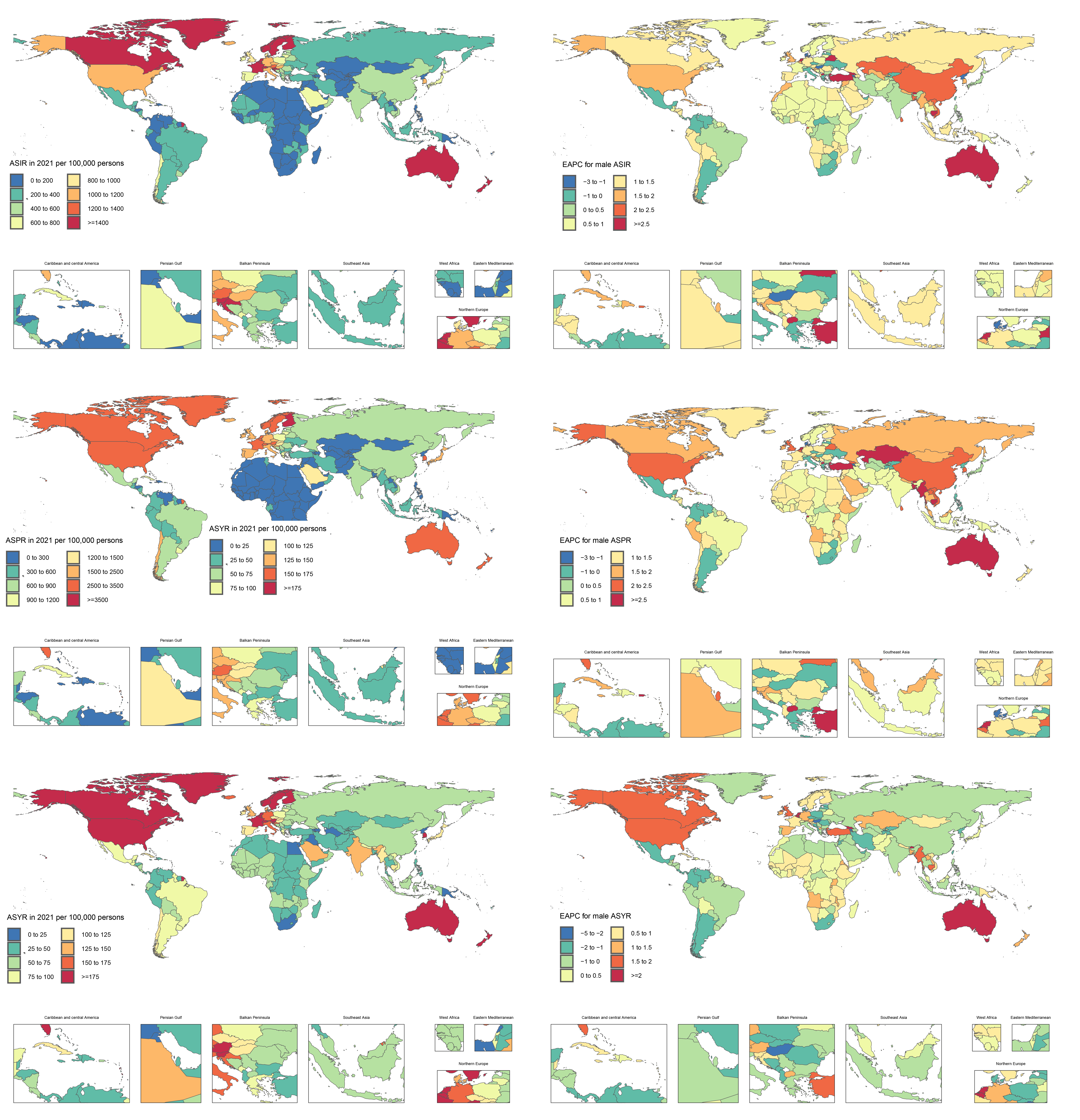


**Figure S3.** The ASIR, ASPR, and ASYR of hip fractures attributable to falls in older male in 204 countries in 2021, as well as the EAPC of ASIR, ASPR, and ASYR from 1990 to 2021.

Abbreviations: ASIR, Age-standardized incidence rate; ASPR, Age-standardized prevalence rate; ASYR, Age-standardized years lived with disability rate; EAPC, Estimated annual percentage change.


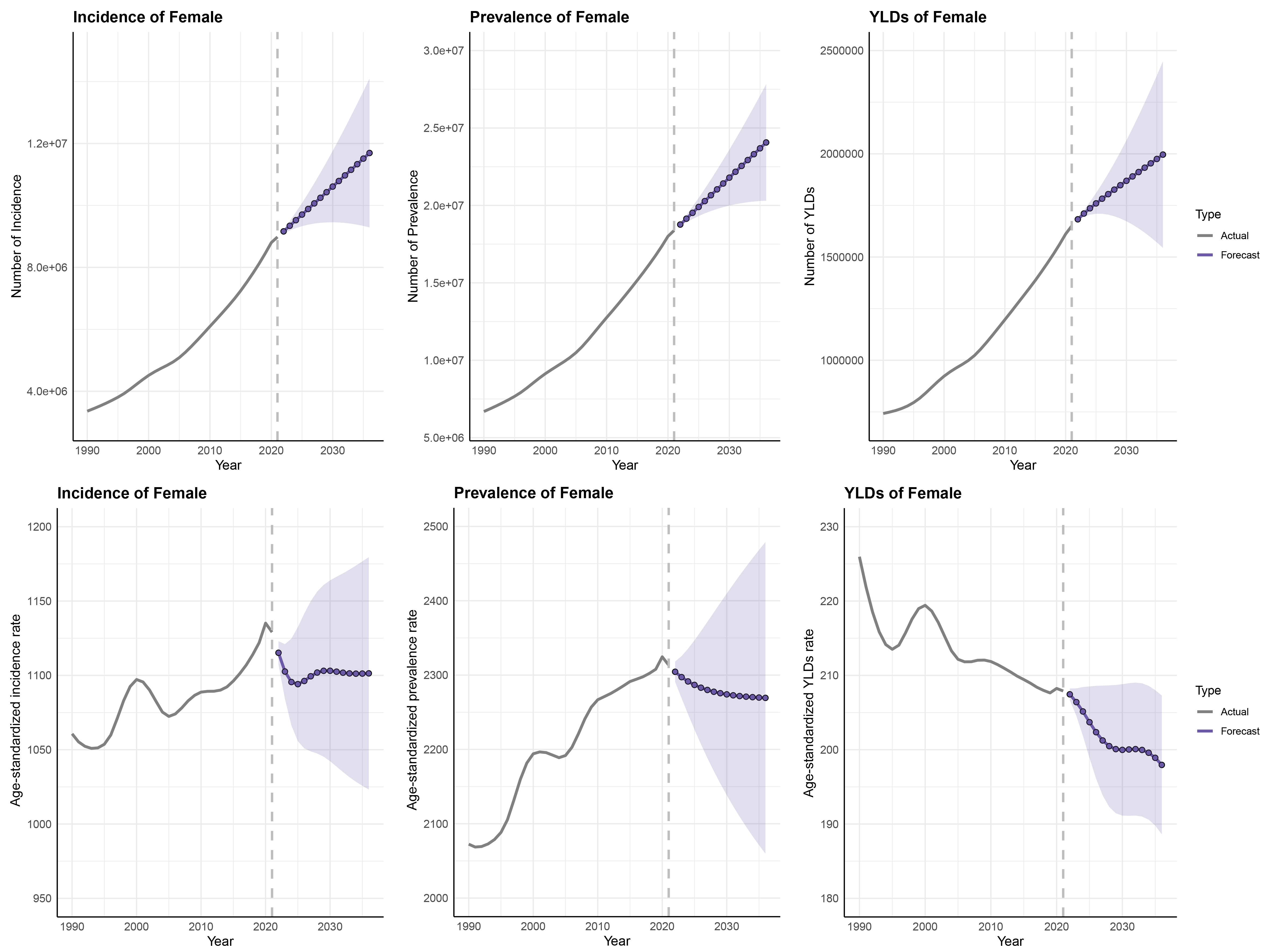


**Figure S4.** Prediction of the disease burden trend of hip fractures attributable to falls in older female from 2022 to 2036.


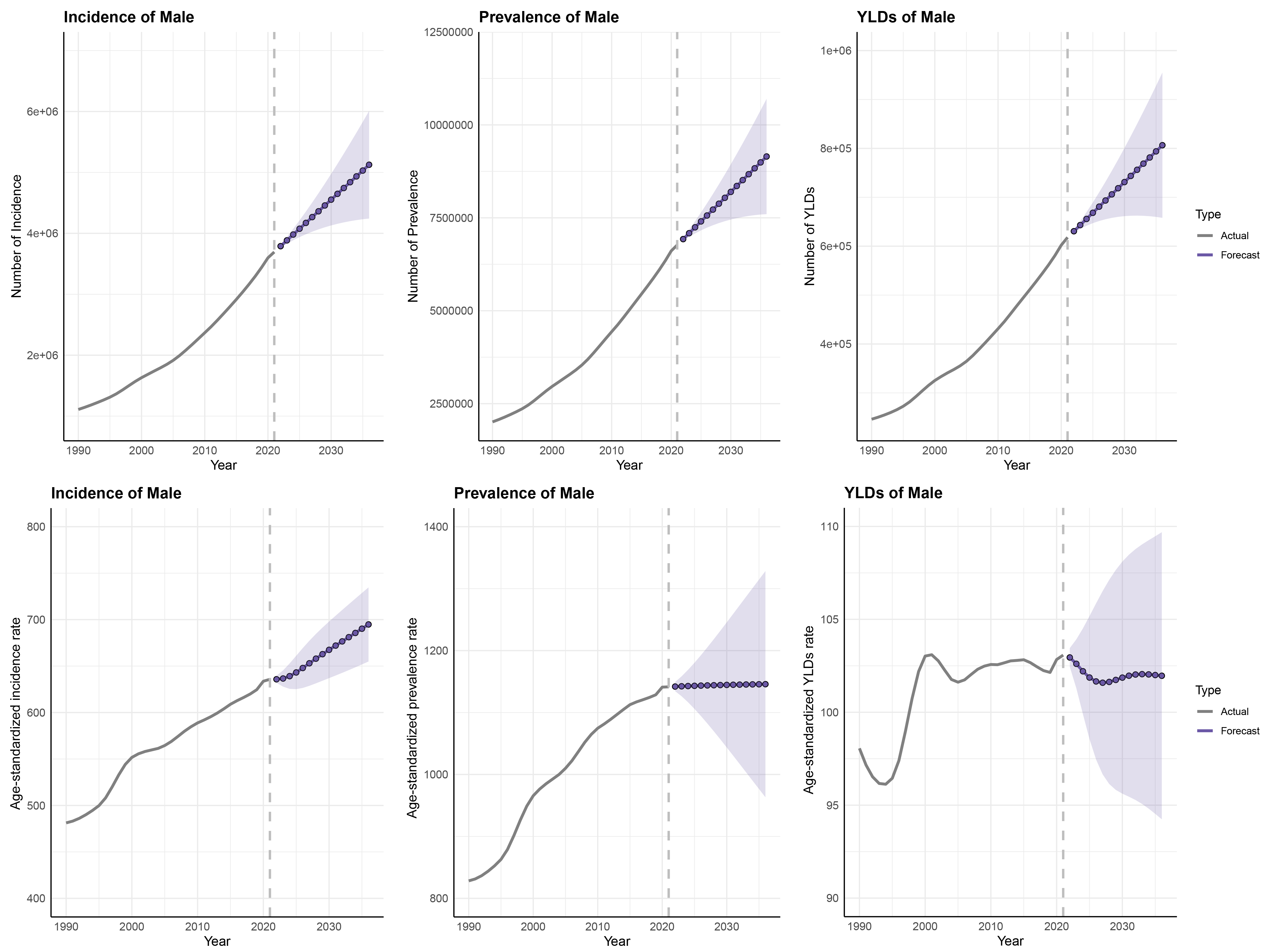


**Figure S5.** Prediction of the disease burden trend of hip fractures attributable to falls in older male from 2022 to 2036.

| TableS1. **The lexis diagram of hip fractures attributable to falls in older adults for the APC model.** | | | | | | | | | | |
| --- | --- | --- | --- | --- | --- | --- | --- | --- | --- | --- |
|  | Age groups | | | | | | | | |  |
| Period (five-year average) | 55-59 | 60-64 | 65-69 | 70-74 | 75-79 | 80-84 | 85-89 | 90-94 | 95+ | Birth cohort |
|  |  |  |  |  |  |  |  |  | X | 1893—1901 |
|  |  |  |  |  |  |  |  | X | X | 1898—1906 |
|  |  |  |  |  |  |  | X | X | X | 1903—1911 |
|  |  |  |  |  |  | X | X | X | X | 1908—1916 |
|  |  |  |  |  | X | X | X | X | X | 1913—1921 |
|  |  |  |  | X | X | X | X | X | X | 1918—1926 |
|  |  |  | X | X | X | X | X | X |  | 1923—1931 |
|  |  | X | X | X | X | X | X |  |  | 1928—1936 |
| 1992—1996 | X | X | X | X | X | X |  |  |  | 1933—1941 |
| 1997—2001 | X | X | X | X | X |  |  |  |  | 1938—1946 |
| 2002—2006 | X | X | X | X |  |  |  |  |  | 1943—1951 |
| 2007—2011 | X | X | X |  |  |  |  |  |  | 1948—1956 |
| 2012—2016 | X | X |  |  |  |  |  |  |  | 1953—1961 |
| 2017—2021 | X |  |  |  |  |  |  |  |  | 1958—1966 |

| **Table S2. The joinpoint regression analysis of Hip fractures attributable to falls in the elderly from 1990 to 2021** | | | | | | | | | | | |
| --- | --- | --- | --- | --- | --- | --- | --- | --- | --- | --- | --- |
|  | **ASIR** | | |  | **ASPR** | | |  | **ASYR** | | |
| **Sex name** | **Range** | **APC (95% CI)** | ***P*** | **Sex name** | **Range** | **APC (95% CI)** | ***P*** | **Sex name** | **Range** | **APC (95% CI)** | ***P*** |
| Both | 1990-1995 | 0.038(-0.125,0.2) | 0.633558 | Both | 1990-1995 | 0.261(0.157,0.365) | 0.000052 | Both | 1990-1992 | -1.627(-2.209,-1.041) | 0.00003 |
| Both | 1995-2000 | 1.167(0.932,1.401) | < 0.001 | Both | 1995-2000 | 1.306(1.159,1.453) | < 0.001 | Both | 1992-1995 | -0.682(-1.274,-0.087) | 0.027516 |
| Both | 2000-2004 | -0.47(-0.834,-0.106) | 0.014345 | Both | 2000-2005 | 0.002(-0.14,0.145) | 0.975279 | Both | 1995-2000 | 0.783(0.592,0.974) | < 0.001 |
| Both | 2004-2015 | 0.222(0.166,0.277) | < 0.001 | Both | 2005-2009 | 0.835(0.612,1.059) | < 0.001 | Both | 2000-2005 | -0.735(-0.923,-0.548) | 0.000001 |
| Both | 2015-2021 | 0.573(0.456,0.69) | < 0.001 | Both | 2009-2021 | 0.219(0.193,0.245) | < 0.001 | Both | 2005-2009 | -0.055(-0.352,0.243) | 0.699205 |
| Female | 1990-1995 | -0.117(-0.29,0.056) | 0.172045 | Female | 1990-1995 | 0.175(0.072,0.278) | 0.002205 | Both | 2009-2021 | -0.209(-0.243,-0.174) | < 0.001 |
| Female | 1995-2000 | 0.919(0.671,1.168) | < 0.001 | Female | 1995-2000 | 1.089(0.944,1.234) | < 0.001 | Female | 1990-1992 | -1.768(-2.246,-1.287) | 0.000001 |
| Female | 2000-2005 | -0.49(-0.735,-0.244) | 0.000554 | Female | 2000-2005 | -0.12(-0.261,0.021) | 0.090609 | Female | 1992-1995 | -0.782(-1.268,-0.293) | 0.003917 |
| Female | 2005-2015 | 0.21(0.141,0.28) | 0.000006 | Female | 2005-2009 | 0.815(0.594,1.037) | < 0.001 | Female | 1995-2000 | 0.662(0.506,0.819) | < 0.001 |
| Female | 2015-2021 | 0.566(0.44,0.692) | < 0.001 | Female | 2009-2021 | 0.22(0.194,0.245) | < 0.001 | Female | 2000-2005 | -0.752(-0.905,-0.599) | < 0.001 |
| Male | 1990-1995 | 0.772(0.633,0.911) | < 0.001 | Male | 1990-1995 | 0.825(0.699,0.951) | < 0.001 | Female | 2005-2009 | 0.006(-0.238,0.251) | 0.957336 |
| Male | 1995-2000 | 2.124(1.924,2.324) | < 0.001 | Male | 1995-2000 | 2.415(2.236,2.594) | < 0.001 | Female | 2009-2021 | -0.188(-0.217,-0.16) | < 0.001 |
| Male | 2000-2005 | 0.36(0.167,0.553) | 0.000995 | Male | 2000-2005 | 0.815(0.642,0.988) | < 0.001 | Male | 1990-1992 | -0.86(-1.523,-0.194) | 0.01493 |
| Male | 2005-2008 | 0.922(0.32,1.527) | 0.004739 | Male | 2005-2009 | 1.378(1.109,1.647) | < 0.001 | Male | 1992-1995 | -0.045(-0.717,0.631) | 0.887989 |
| Male | 2008-2021 | 0.702(0.672,0.733) | < 0.001 | Male | 2009-2014 | 0.738(0.572,0.904) | < 0.001 | Male | 1995-2000 | 1.449(1.233,1.665) | < 0.001 |
|  |  |  |  | Male | 2014-2021 | 0.466(0.397,0.535) | < 0.001 | Male | 2000-2005 | -0.367(-0.578,-0.156) | 0.002148 |
|  |  |  |  |  |  |  |  | Male | 2005-2009 | 0.259(-0.075,0.593) | 0.119147 |
|  |  |  |  |  |  |  |  | Male | 2009-2021 | 0.007(-0.033,0.046) | 0.716189 |
| **Sex name** | **Range** | **AAPC (95% CI)** | ***P*** | **Sex name** | **Range** | **AAPC (95% CI)** | ***P*** | **Sex name** | **Range** | **AAPC (95% CI)** | ***P*** |
| Both | 1990-2021 | 0.322(0.254,0.389) | < 0.001 | Both | 1990-2021 | 0.445(0.4,0.489) | < 0.001 | Both | 1990-2021 | -0.253(-0.337,-0.169) | < 0.001 |
| Female | 1990-2021 | 0.227(0.16,0.293) | < 0.001 | Female | 1990-2021 | 0.374(0.33,0.418) | < 0.001 | Female | 1990-2021 | -0.278(-0.347,-0.209) | < 0.001 |
| Male | 1990-2021 | 0.907(0.835,0.98) | 0 | Male | 1990-2021 | 1.054(0.994,1.113) | 0 | Male | 1990-2021 | 0.149(0.054,0.243) | 0 |

| **Table S3. The ASIR for 1990 and 2021, and the EAPC of ASIR from 1990 to 2021.** | | | | | | | | | |
| --- | --- | --- | --- | --- | --- | --- | --- | --- | --- |
|  | **Both** | | | **Female** | | | **Male** | | |
|  | **ASIR per 100,000 population, 1990** | **ASIR per 100,000 population, 2021** | **EAPC** | **ASIR per 100,000 population, 1990** | **ASIR per 100,000 population, 2021** | **EAPC** | **ASIR per 100,000 population, 1990** | **ASIR per 100,000 population, 2021** | **EAPC** |
| Global | 847.64 (559.84,1222.97) | 930.73 (624.14,1317.39) | 0.28 (0.25,0.32) | 1060.7 (699.29,1533.1) | 1129.07 (754.2,1607.57) | 0.19 (0.15,0.23) | 481.23 (313.67,704.35) | 635.76 (426.75,904.48) | 0.91 (0.84,0.97) |
| High SDI | 1312.11 (886.91,1844.73) | 1469.17 (1013.83,2039.11) | 0.46 (0.4,0.52) | 1580.2 (1066.5,2223.69) | 1759.75 (1206.66,2451.1) | 0.45 (0.4,0.49) | 783.57 (524.35,1114.97) | 1047.62 (721.94,1462.29) | 1.05 (0.92,1.18) |
| High-middle SDI | 721.82 (465.25,1070.6) | 778.49 (517.38,1117.05) | 0.07 (-0.02,0.16) | 845.96 (542.69,1258.85) | 887.21 (586.89,1273.1) | -0.01 (-0.11,0.08) | 459.71 (295.67,682.51) | 582.37 (384.59,840.41) | 0.63 (0.55,0.71) |
| Middle SDI | 413.54 (251.23,645.72) | 639.98 (403.63,959.32) | 1.22 (1.02,1.42) | 524.92 (319,819.28) | 790.44 (496.88,1188.72) | 1.14 (0.95,1.34) | 259.47 (155.86,404.29) | 426.5 (267.04,638.39) | 1.41 (1.2,1.62) |
| Low-middle SDI | 514.2 (305.26,812.52) | 632.05 (396.82,951.91) | 0.62 (0.55,0.68) | 734.32 (435.46,1159.78) | 869.12 (542.86,1314.89) | 0.49 (0.44,0.55) | 285.01 (170.38,445.23) | 340.57 (213.56,513.3) | 0.48 (0.39,0.57) |
| Low SDI | 349.48 (214.35,539.27) | 464.74 (293.86,693.83) | 0.86 (0.81,0.92) | 476.37 (290.56,740.27) | 628.83 (396.28,948.19) | 0.84 (0.79,0.9) | 213.12 (130.43,325.68) | 277.59 (175.78,413.68) | 0.77 (0.69,0.85) |
| Andean Latin America | 153.41 (100.36,220.59) | 238.1 (158.16,337.8) | 1.37 (1.3,1.44) | 173.31 (113.61,249) | 266.46 (175.92,378.45) | 1.35 (1.27,1.43) | 128.67 (83.53,186.88) | 200.33 (131.72,288.98) | 1.39 (1.32,1.46) |
| Australasia | 1606.97 (1109.79,2204.07) | 2358.9 (1676.77,3151.75) | 1.71 (1.54,1.88) | 1950.05 (1340.31,2704.13) | 2812.56 (1963.16,3808.39) | 1.68 (1.52,1.84) | 978.35 (676.87,1344.15) | 1758.99 (1241.18,2363.46) | 2.3 (2.07,2.53) |
| Caribbean | 511.63 (349.57,720.07) | 808.03 (557.69,1109.79) | 1.38 (1.26,1.51) | 703.74 (473.66,996.45) | 1079.17 (741.17,1482.28) | 1.26 (1.13,1.4) | 285.18 (194.87,402.17) | 445.31 (306.2,621.55) | 1.44 (1.38,1.5) |
| Central Asia | 122.83 (80.39,177.83) | 165.74 (111.1,236.12) | 1.53 (1.31,1.76) | 124.52 (80.19,181.76) | 172.83 (114.77,247.12) | 1.66 (1.4,1.92) | 115.84 (76.64,167.33) | 146.83 (98.95,209.54) | 1.25 (1.08,1.43) |
| Central Europe | 1177.31 (800.56,1668.37) | 902.44 (608.36,1277.38) | -1.11 (-1.2,-1.03) | 1432.66 (971.43,2033) | 963.63 (648.07,1354.97) | -1.57 (-1.67,-1.47) | 668.81 (446.08,954.51) | 732.93 (493.24,1045.29) | 0.15 (0.06,0.24) |
| Central Latin America | 493.18 (311.96,740.62) | 400.49 (262.17,582.09) | -0.69 (-0.82,-0.56) | 625.19 (396.86,934.14) | 478.5 (313.39,694.46) | -0.85 (-1.01,-0.7) | 345.86 (215.6,524.07) | 294.88 (190.77,430.97) | -0.57 (-0.66,-0.47) |
| Central Sub-Saharan Africa | 185.97 (120.62,270.38) | 250.06 (165.8,357.62) | 0.9 (0.8,1) | 221.51 (142.6,323.02) | 292.03 (192.49,420.06) | 0.84 (0.72,0.97) | 145.81 (93.48,212.64) | 175.97 (115.77,253.72) | 0.53 (0.41,0.64) |
| East Asia | 383.44 (225.21,610.04) | 729.25 (454.35,1099.14) | 1.75 (1.24,2.27) | 450.95 (265.06,720.95) | 826.96 (513.76,1255.79) | 1.62 (1.09,2.15) | 271 (157.42,430.25) | 563.98 (352.28,848.06) | 2.18 (1.75,2.62) |
| Eastern Europe | 301.05 (179.15,472.88) | 349.37 (215.99,530.75) | 0.48 (0.3,0.66) | 292.98 (173.85,462.61) | 321.74 (198.53,490.12) | 0.28 (0.21,0.36) | 286.11 (169.22,450.17) | 367.32 (225.83,563.13) | 0.84 (0.49,1.2) |
| Eastern Sub-Saharan Africa | 199.12 (125.75,295.62) | 264.42 (173.18,382.9) | 0.85 (0.8,0.9) | 247.07 (155.33,368.49) | 326.5 (213.06,472.54) | 0.82 (0.75,0.89) | 143.74 (90.65,214.08) | 181.06 (117.4,263.64) | 0.7 (0.68,0.72) |
| High-income Asia Pacific | 758.25 (463.52,1164.82) | 821.59 (542.76,1180.45) | 0.16 (0.02,0.29) | 829.73 (506.78,1273.25) | 854.1 (561.17,1224.98) | -0.03 (-0.13,0.06) | 590.97 (357.95,913.14) | 742.11 (486.46,1075.23) | 0.68 (0.45,0.91) |
| High-income North America | 1059.81 (660.84,1603) | 1628.82 (1049.14,2361.69) | 1.47 (1.21,1.72) | 1237.61 (768.84,1878.19) | 1958.93 (1260.11,2853.16) | 1.57 (1.32,1.83) | 750.12 (473.5,1131.27) | 1196.77 (782.02,1735.82) | 1.64 (1.4,1.87) |
| North Africa and Middle East | 190.89 (122.22,280.58) | 344.84 (228.05,490.58) | 2.19 (2.05,2.34) | 252.22 (161.8,371.55) | 475.98 (314.78,675.86) | 2.42 (2.26,2.58) | 120.45 (76.02,177.76) | 208.64 (136.76,299.85) | 1.88 (1.8,1.95) |
| Oceania | 351.2 (222.87,522.97) | 520.72 (340.69,755.43) | 1.11 (0.98,1.24) | 597.58 (375.9,898.17) | 931.56 (605.63,1357.03) | 1.28 (1.15,1.42) | 72.95 (47.52,104.97) | 86.6 (57.35,123.59) | 0.5 (0.48,0.52) |
| South Asia | 753.23 (441.47,1202.49) | 946.17 (585.09,1438.7) | 0.63 (0.57,0.69) | 1137.8 (666.79,1812.74) | 1344.99 (825.73,2048.41) | 0.41 (0.34,0.47) | 386.23 (226.03,612.44) | 475.31 (292.67,723.91) | 0.53 (0.43,0.62) |
| Southeast Asia | 360.55 (225.91,543.18) | 481.08 (314.61,696.14) | 0.87 (0.81,0.92) | 485.45 (303.27,733.85) | 614.57 (402.37,891.28) | 0.65 (0.59,0.72) | 181.9 (112.85,275.34) | 273.24 (176.52,401.1) | 1.31 (1.28,1.35) |
| Southern Latin America | 618.61 (431.16,849.52) | 725.07 (511.65,977.89) | 0.63 (0.49,0.78) | 778.67 (537.33,1078.06) | 914.87 (641.03,1243.4) | 0.68 (0.51,0.84) | 356.12 (247.66,490.12) | 404.45 (280.85,551.93) | 0.41 (0.34,0.49) |
| Southern Sub-Saharan Africa | 123.29 (75.93,189.64) | 120.06 (75.99,179.82) | -0.47 (-0.6,-0.34) | 135.56 (83.15,209.37) | 128.04 (80.46,191.92) | -0.58 (-0.7,-0.45) | 98.88 (59.82,151.87) | 100.31 (62.65,150.75) | -0.3 (-0.45,-0.16) |
| Tropical Latin America | 450.73 (265.02,712.77) | 523.73 (325.24,793.13) | 0.56 (0.53,0.58) | 529.99 (313.34,841.82) | 617.85 (382.96,936.12) | 0.61 (0.56,0.66) | 335.76 (195.42,532.62) | 378.83 (233.4,575.29) | 0.37 (0.24,0.49) |
| Western Europe | 1756.22 (1213.24,2414.97) | 1733.8 (1230.39,2332.91) | 0.06 (0.01,0.12) | 2175.65 (1493.75,3006.73) | 2168.67 (1525.87,2921.99) | 0.1 (0.03,0.17) | 915.27 (630.05,1264.94) | 1092.72 (778.98,1480.55) | 0.71 (0.64,0.77) |
| Western Sub-Saharan Africa | 244.47 (153.51,368.74) | 317.88 (205.03,464.67) | 0.83 (0.78,0.88) | 310.63 (194.19,471.9) | 410.75 (264.39,601.55) | 0.91 (0.86,0.95) | 160.4 (100.08,240.96) | 210.85 (136.27,308.21) | 0.91 (0.85,0.97) |
| Afghanistan | 112.77 (72.16,166.45) | 153.78 (102.09,220.53) | 0.98 (0.91,1.05) | 154.93 (98.84,229.7) | 215.82 (142.06,310.13) | 1.06 (1,1.12) | 78.58 (49.25,117.69) | 97.84 (63.63,141.41) | 0.73 (0.65,0.82) |
| Albania | 205.06 (126.22,313.31) | 263.34 (169.74,380.06) | 0.99 (0.68,1.29) | 233.65 (140.89,362.16) | 307.78 (196.72,448.27) | 1.15 (0.83,1.47) | 148.51 (92.67,221.98) | 204.05 (130.83,297.52) | 1.16 (0.89,1.43) |
| Algeria | 176.26 (113.48,256.47) | 257.58 (172.29,366.71) | 1.11 (1.01,1.2) | 240.64 (154.64,353.9) | 365.7 (243.98,522.87) | 1.41 (1.37,1.45) | 135.59 (86.52,199.52) | 179.08 (117.27,255.67) | 0.76 (0.71,0.82) |
| American Samoa | 246.43 (161.69,352.42) | 337.75 (226.8,475.06) | 1.14 (1.09,1.19) | 254.68 (166.63,369.27) | 407.97 (269.91,579.53) | 1.74 (1.66,1.81) | 224.54 (145.77,325.37) | 252.45 (168.33,361.62) | 0.31 (0.24,0.38) |
| Andorra | 2577.57 (1723.04,3661.96) | 3379.92 (2334.7,4603.8) | 0.98 (0.93,1.04) | 3774.72 (2492.05,5424.31) | 5166.82 (3522.98,7125.08) | 1.07 (1.02,1.12) | 1172.81 (787.95,1649.64) | 1261.56 (878.78,1732.02) | 0.18 (0.11,0.25) |
| Angola | 162.87 (104.78,237.56) | 259.34 (172.32,370.92) | 1.52 (1.46,1.58) | 184.84 (117.15,274.26) | 300.13 (197.12,432.58) | 1.61 (1.55,1.67) | 134.51 (86.31,197.11) | 192.96 (127.57,279.86) | 1.17 (1.11,1.23) |
| Antigua and Barbuda | 159.23 (105.65,227.79) | 269.17 (183.14,375.49) | 1.72 (1.63,1.82) | 156.88 (101.52,228.89) | 300.61 (200.98,425.34) | 2.06 (1.87,2.25) | 160.89 (107.18,229.4) | 223.56 (151.65,314.83) | 1.21 (1.09,1.33) |
| Argentina | 573.47 (395.83,789.09) | 507.05 (352.79,695.65) | -0.45 (-0.59,-0.31) | 725.42 (495.74,1012.1) | 637.85 (438.33,884.95) | -0.43 (-0.59,-0.28) | 323.36 (222.15,449.01) | 286.97 (196.49,395.51) | -0.55 (-0.65,-0.45) |
| Armenia | 265.01 (175.99,383.04) | 182.03 (121.22,260.62) | -0.99 (-1.48,-0.48) | 299.38 (195.77,435.31) | 218.69 (143.73,314.06) | -0.61 (-1.23,0.02) | 204.74 (134.79,298.12) | 121.87 (81.58,173.95) | -1.85 (-2.13,-1.57) |
| Australia | 1484.58 (1040.8,2017.77) | 2413.69 (1714.57,3225.45) | 2 (1.83,2.18) | 1790.73 (1238.18,2459.03) | 2866.82 (2000.94,3898.28) | 1.97 (1.81,2.13) | 928.34 (644.73,1275.97) | 1815.28 (1278.09,2446.62) | 2.56 (2.32,2.79) |
| Austria | 2035.13 (1427.04,2763.13) | 1876.38 (1316.98,2536.2) | 0.19 (0.01,0.37) | 2437.4 (1697.33,3336.14) | 2201.74 (1517.85,3017.63) | 0.16 (-0.06,0.38) | 1102.36 (764.4,1519.44) | 1358.84 (945.72,1848.71) | 1.03 (0.88,1.18) |
| Azerbaijan | 83.67 (53.4,123.31) | 111.91 (72.48,162.79) | 1.31 (1.09,1.52) | 84.9 (53.03,127.07) | 116.66 (73.88,172.52) | 1.43 (1.19,1.67) | 77.63 (49.8,114.07) | 101.75 (66.26,147.51) | 1.21 (1.02,1.41) |
| Bahamas | 220.77 (147.8,313.53) | 295.51 (200.28,412.5) | 0.84 (0.65,1.03) | 253.06 (166.37,363.83) | 357.59 (238.99,505.9) | 1.05 (0.84,1.26) | 159.26 (106.08,226.66) | 201.97 (136.59,283.97) | 0.67 (0.56,0.79) |
| Bahrain | 160.73 (104.5,231.9) | 223.53 (149.99,314.66) | 1.03 (0.94,1.12) | 165.68 (107.45,240.84) | 262.36 (172.76,374.17) | 1.47 (1.38,1.56) | 145.69 (92.74,213.74) | 172.71 (114.95,247.73) | 0.45 (0.36,0.53) |
| Bangladesh | 59.03 (37.36,87.01) | 106.37 (69.55,153.38) | 2.19 (1.71,2.67) | 62.96 (39.04,95.22) | 115.99 (74.74,168.58) | 2.32 (1.68,2.95) | 56.41 (35.7,83.07) | 97.93 (64.12,141.87) | 2 (1.68,2.31) |
| Barbados | 198.68 (133.7,281.81) | 301.53 (203.51,418.16) | 1.38 (1.24,1.51) | 244.27 (161.87,351.56) | 407.89 (273.64,569.19) | 1.69 (1.55,1.83) | 114.6 (76.15,163.75) | 155.17 (105.4,218.36) | 0.9 (0.8,1) |
| Belarus | 230.13 (150.17,333.63) | 433.39 (286.34,625.68) | 2.16 (1.73,2.59) | 221.04 (141.57,324.08) | 387.65 (253.47,562.93) | 1.78 (1.47,2.1) | 235.78 (153.58,345.56) | 469.88 (306.67,693.33) | 2.58 (1.99,3.16) |
| Belgium | 1875.76 (1302.9,2557.49) | 2524.18 (1765.79,3415.19) | 1.15 (0.99,1.3) | 2389.44 (1645.26,3282.71) | 3301.03 (2261.16,4525.66) | 1.25 (1.1,1.4) | 881.63 (619.64,1208.85) | 1415 (991.98,1927.19) | 1.73 (1.54,1.92) |
| Belize | 156.22 (103.98,221.06) | 231.87 (157.36,323.67) | 1.12 (0.94,1.3) | 192.94 (127.35,277.17) | 276.74 (184.52,389.95) | 1.06 (0.88,1.25) | 111.08 (74,158.61) | 185.36 (124.74,261.5) | 1.41 (1.24,1.59) |
| Benin | 217.14 (139.46,316.48) | 273.74 (180.9,394.08) | 0.64 (0.58,0.69) | 264.13 (168.71,388.19) | 319.73 (209.11,463) | 0.48 (0.42,0.53) | 165.74 (106.71,243.09) | 205.45 (134.56,296.11) | 0.59 (0.54,0.65) |
| Bermuda | 262.77 (176.12,372.01) | 367.57 (250.37,512.98) | 0.82 (0.63,1.01) | 254.18 (166.76,366.25) | 341.26 (226.85,482.47) | 0.56 (0.3,0.81) | 271.76 (181.89,387.15) | 402.98 (274.46,568.46) | 1.23 (1.14,1.33) |
| Bhutan | 298.94 (191.6,439.13) | 805.53 (525.32,1156.64) | 3.36 (3.15,3.57) | 372.33 (235.9,553.19) | 1100.65 (718.32,1588.23) | 3.63 (3.35,3.92) | 198.97 (126.74,292.94) | 500.8 (319.76,732.09) | 3.18 (3.02,3.33) |
| Bolivia (Plurinational State of) | 171.9 (109.18,252.74) | 281.27 (184.91,401.41) | 1.69 (1.65,1.73) | 185.4 (117.27,275.44) | 317.93 (206.74,460.34) | 1.87 (1.8,1.95) | 149.57 (94.14,223.11) | 230.17 (148.09,335.02) | 1.44 (1.4,1.48) |
| Bosnia and Herzegovina | 407.01 (257.34,595.65) | 443.29 (294.97,631.52) | 0.54 (0.26,0.83) | 407.42 (255.93,607.68) | 430.07 (279.52,620.19) | 0.47 (0.19,0.76) | 393.01 (249.94,574.14) | 446.97 (295.55,644.53) | 0.62 (0.35,0.89) |
| Botswana | 154.75 (99.42,225.29) | 184.68 (121.74,267.06) | 0.39 (0.34,0.45) | 163.96 (104.82,240.77) | 191.53 (124.04,279.36) | 0.36 (0.29,0.43) | 132.71 (83.91,195.86) | 161.44 (105.18,233.52) | 0.35 (0.25,0.45) |
| Brazil | 456.55 (267.38,723.91) | 528.08 (327.22,800.85) | 0.54 (0.52,0.57) | 536.92 (316.48,854.93) | 622.85 (385.44,944.71) | 0.59 (0.54,0.64) | 339.82 (197.19,540.38) | 382.06 (234.84,581.04) | 0.35 (0.22,0.48) |
| Brunei Darussalam | 580.28 (388.75,817.69) | 796.7 (553.79,1092.79) | 1 (0.86,1.13) | 631.01 (419.61,895.98) | 903.9 (616.98,1256.3) | 1.03 (0.75,1.31) | 506.52 (336.37,723.19) | 633.52 (439.28,874.01) | 0.97 (0.89,1.06) |
| Bulgaria | 289.47 (191.89,418.52) | 305 (203.65,439.13) | 0.34 (0.15,0.52) | 277.54 (180.45,403.69) | 293.96 (192.41,427.85) | 0.48 (0.26,0.71) | 299.48 (197.22,435.33) | 306.19 (202.31,443.8) | 0.09 (-0.06,0.24) |
| Burkina Faso | 253.11 (164.08,365.5) | 290.94 (191.72,414.71) | 0.38 (0.35,0.41) | 326.41 (209.6,476.62) | 361.19 (237.26,520.57) | 0.24 (0.21,0.27) | 165.51 (106.62,241.27) | 197.98 (129.67,286.4) | 0.59 (0.54,0.64) |
| Burundi | 207.05 (130.98,304.92) | 213.27 (140.92,304.7) | -0.11 (-0.17,-0.05) | 246.88 (155.27,366.95) | 260.36 (169.84,376.83) | -0.1 (-0.18,-0.02) | 153.77 (97.15,227.05) | 159.22 (104.4,229.73) | 0.05 (0.01,0.08) |
| Cabo Verde | 193.68 (125.43,278.9) | 343.44 (229.09,488.84) | 1.97 (1.87,2.08) | 231.42 (150.51,334.75) | 353.35 (236.09,503.82) | 1.58 (1.44,1.72) | 140.66 (89.82,206.44) | 305.27 (200.72,441.14) | 2.44 (2.29,2.6) |
| Cambodia | 316.74 (204.31,461.23) | 681.16 (450.7,972.6) | 2.7 (2.58,2.83) | 382.46 (246.12,558.87) | 805.55 (528.14,1156.56) | 2.63 (2.51,2.74) | 206.82 (131.84,306.49) | 432.74 (282.99,626.21) | 2.67 (2.53,2.81) |
| Cameroon | 255.77 (165.88,370.48) | 316.52 (209.79,450.64) | 0.56 (0.48,0.64) | 315.18 (200.92,461.76) | 374.13 (244.38,537.93) | 0.43 (0.32,0.54) | 189.03 (122.47,275.63) | 237.92 (156.76,343.81) | 0.59 (0.54,0.65) |
| Canada | 1608.07 (1134.32,2183.6) | 2217.84 (1565.63,2989.85) | 1.25 (1.05,1.45) | 1886.1 (1307.8,2579.21) | 2680.26 (1869.18,3652.36) | 1.38 (1.17,1.58) | 1114.83 (778.93,1525.51) | 1588.3 (1122.04,2147.62) | 1.34 (1.19,1.5) |
| Central African Republic | 166.35 (106.04,243.39) | 169.92 (110.65,246.99) | -0.05 (-0.09,-0.02) | 181.04 (114.51,268.49) | 186.41 (119.95,273.76) | -0.05 (-0.09,-0.01) | 135.74 (86.04,200.58) | 132.09 (86.17,192.82) | -0.16 (-0.2,-0.13) |
| Chad | 184.93 (118.73,269.86) | 209.31 (136.66,302.27) | 0.39 (0.35,0.43) | 227.61 (144.51,335.58) | 262.53 (169.58,379.69) | 0.43 (0.38,0.47) | 134.17 (85.31,197.89) | 161.41 (104.42,236.61) | 0.57 (0.54,0.6) |
| Chile | 735.37 (505.79,1017.89) | 1174.44 (828.49,1588.43) | 1.99 (1.83,2.15) | 923.68 (630,1283.97) | 1519.59 (1058.92,2070.78) | 2.14 (1.96,2.33) | 454.73 (306.77,639.8) | 640.47 (443.88,880.64) | 1.38 (1.27,1.49) |
| China | 390.04 (227.3,623.67) | 752.55 (467.79,1136.02) | 1.8 (1.27,2.32) | 459.96 (268.44,738.22) | 854.06 (529.61,1298.25) | 1.66 (1.12,2.2) | 273.58 (157.52,436.82) | 581.39 (362.43,875.86) | 2.26 (1.82,2.7) |
| Colombia | 322.07 (213.89,458.26) | 231.04 (153.44,330.61) | -1.1 (-1.36,-0.84) | 402.74 (265.47,580.13) | 264.17 (172.46,381.05) | -1.44 (-1.74,-1.13) | 227.55 (150.13,331.44) | 183.63 (121.62,263.19) | -0.64 (-0.81,-0.46) |
| Comoros | 161.2 (103.67,232.9) | 207.05 (136.69,296.32) | 0.78 (0.67,0.89) | 194.5 (123.86,285.56) | 252.96 (165.96,365.65) | 0.78 (0.66,0.89) | 118.98 (76.18,174.01) | 147.85 (96.97,214.37) | 0.75 (0.65,0.86) |
| Congo | 212.09 (137.11,308.92) | 256.61 (170.83,369.08) | 0.58 (0.43,0.72) | 242.93 (155.68,357.22) | 309.98 (202.7,449.05) | 0.74 (0.6,0.88) | 164.39 (106.01,239.99) | 185.84 (122.43,269.19) | 0.38 (0.2,0.57) |
| Cook Islands | 199.46 (130.77,289.21) | 288.42 (193.44,409.01) | 1.25 (1.2,1.3) | 204.15 (132.42,299.71) | 293.74 (194.06,423.45) | 1.28 (1.19,1.37) | 183.7 (118.35,268.82) | 269.08 (176.89,390.15) | 1.31 (1.26,1.36) |
| Costa Rica | 727.27 (491.32,1011.18) | 669.3 (454.39,933.83) | -0.39 (-0.49,-0.28) | 1016.93 (682.8,1422.97) | 866.41 (584.89,1207.47) | -0.65 (-0.78,-0.53) | 403.41 (270.19,573.67) | 403.91 (271.81,570.94) | 0.02 (-0.07,0.11) |
| Croatia | 1513.08 (1011.42,2146.18) | 2374.01 (1621.33,3277.24) | 1.8 (1.62,1.98) | 1809.64 (1205.06,2578.36) | 2831.77 (1922.04,3932.43) | 1.86 (1.69,2.03) | 857.07 (568.12,1222.18) | 1444.14 (979.7,2021.09) | 1.78 (1.55,2.01) |
| Cuba | 900.9 (612.94,1269.24) | 1564.38 (1072.35,2152.6) | 1.61 (1.45,1.77) | 1332.51 (891.66,1887.31) | 2166.65 (1475.57,2992.96) | 1.42 (1.27,1.57) | 446.21 (302.03,633.57) | 769.78 (522.03,1078.12) | 1.68 (1.59,1.77) |
| Cyprus | 2042.02 (1396.75,2832.85) | 2189.62 (1552.09,2939.08) | 0.37 (0.23,0.51) | 2765.37 (1881.21,3868.86) | 2900.31 (2037.93,3933.35) | 0.12 (0.04,0.21) | 933.75 (630.75,1304.3) | 1146.8 (798.04,1570.55) | 0.7 (0.37,1.04) |
| Czechia | 2643.05 (1782.58,3689.95) | 1347.47 (911.51,1893.95) | -2.77 (-2.94,-2.59) | 3287.05 (2205.1,4601.29) | 1474.15 (984.5,2080.08) | -3.24 (-3.44,-3.05) | 1216.42 (815.88,1724.6) | 1069.39 (715.26,1522.46) | -0.82 (-0.99,-0.66) |
| C?te d'Ivoire | 231.28 (149.95,332.22) | 290.9 (192.84,413.32) | 0.61 (0.54,0.68) | 274.64 (177.62,400.72) | 344.28 (226.01,493.95) | 0.66 (0.56,0.77) | 183.89 (118.4,268.64) | 226.2 (148.13,327.29) | 0.47 (0.41,0.54) |
| Democratic People's Republic of Korea | 185.87 (119.04,273.67) | 134.1 (88.57,192.77) | -0.95 (-1.02,-0.88) | 197.62 (125.37,294.1) | 149.69 (98.06,216.92) | -0.78 (-0.86,-0.71) | 151.49 (96.63,223.47) | 90.28 (59.09,130.68) | -1.67 (-1.73,-1.62) |
| Democratic Republic of the Congo | 186.22 (120.37,271.65) | 246.54 (161.85,356.36) | 0.84 (0.74,0.95) | 227.38 (146.33,335.86) | 290.44 (188.78,421.49) | 0.74 (0.6,0.88) | 144.53 (91.7,211.82) | 166.83 (108.43,242.77) | 0.35 (0.23,0.47) |
| Denmark | 2757.69 (1942.38,3695.95) | 1744.3 (1251.64,2332.66) | -1.96 (-2.11,-1.81) | 3548.34 (2471.7,4799.07) | 2262 (1596.96,3058.84) | -1.93 (-2.08,-1.78) | 1391.6 (974.46,1884.88) | 998.16 (705.14,1349.33) | -1.57 (-1.73,-1.41) |
| Djibouti | 176.99 (113.33,259.46) | 233.01 (153.53,335.3) | 0.83 (0.74,0.93) | 212.63 (135.41,314.23) | 283.98 (184.63,412.07) | 0.83 (0.74,0.93) | 127.15 (80.39,187.89) | 172.52 (111.95,252.32) | 1.01 (0.93,1.1) |
| Dominica | 138.84 (91.11,200.05) | 184.27 (124.06,262.36) | 0.9 (0.78,1.02) | 123.98 (79.31,183.91) | 166.7 (109.83,240.36) | 1 (0.84,1.16) | 166.56 (110.1,239.92) | 214.47 (143,306.89) | 0.7 (0.56,0.84) |
| Dominican Republic | 155.42 (103.4,223.32) | 238.82 (160.29,334.63) | 1.48 (1.32,1.65) | 210.77 (139.48,303.84) | 301.95 (201.72,424.46) | 1.26 (1.13,1.39) | 97.65 (63.87,141.6) | 158.82 (104.66,228.64) | 1.57 (1.41,1.73) |
| Ecuador | 199.91 (132.18,286.28) | 313.66 (211.34,442.87) | 1.47 (1.38,1.56) | 224.57 (147.64,322.39) | 345.12 (232.07,490.45) | 1.46 (1.39,1.53) | 169.27 (110.59,246.04) | 262.14 (173.79,375.62) | 1.37 (1.23,1.51) |
| Egypt | 97.99 (62.42,145.7) | 147.13 (95.68,214.44) | 1.24 (1.1,1.38) | 160.22 (101.98,238.62) | 296.51 (193.97,426.91) | 2 (1.87,2.13) | 49.72 (31.35,73.89) | 71.32 (45.6,105.17) | 1.03 (0.9,1.17) |
| El Salvador | 284.55 (185.11,409.9) | 420.17 (282.47,592.5) | 1.36 (1.23,1.49) | 337.13 (218.28,490.71) | 500.64 (333.67,709.74) | 1.45 (1.34,1.56) | 218.26 (141.8,316.14) | 301.92 (200.53,432.03) | 1.03 (0.84,1.23) |
| Equatorial Guinea | 156.43 (100.29,230.22) | 305.33 (202.99,436.58) | 2.4 (2.31,2.49) | 172.32 (109.06,256.98) | 352.06 (232.04,507.35) | 2.58 (2.47,2.69) | 130.06 (82.95,190.94) | 232.07 (152.91,334.25) | 2.13 (2.06,2.21) |
| Eritrea | 160.92 (101.74,240.4) | 233.52 (153.18,334.51) | 0.99 (0.87,1.1) | 180.37 (113.04,271.81) | 271.59 (176.86,391.98) | 1.1 (1,1.21) | 112.39 (70.68,166.06) | 149.54 (97.48,218.22) | 0.73 (0.61,0.84) |
| Estonia | 593.96 (395.46,848.74) | 421.46 (279.88,601.28) | -1.14 (-1.19,-1.08) | 615.26 (406.89,884.67) | 358.39 (236.52,516.18) | -1.9 (-2.02,-1.79) | 472.35 (309.15,688.6) | 484.72 (318.83,700.12) | 0.24 (0.05,0.43) |
| Eswatini | 126.42 (81.86,184.49) | 141.89 (93.58,205.76) | 0.21 (0.14,0.27) | 131.46 (84.32,193.72) | 145.01 (94.07,210.29) | 0.15 (0.07,0.24) | 106.97 (68.73,156.12) | 122.44 (80.14,178.73) | 0.32 (0.27,0.38) |
| Ethiopia | 219.14 (128.22,348.92) | 262.51 (159.08,401.64) | 0.56 (0.42,0.69) | 278.77 (162.73,445.74) | 350.74 (212.83,537.3) | 0.65 (0.55,0.75) | 150.41 (86.74,240.64) | 176.96 (107.11,270.94) | 0.59 (0.5,0.67) |
| Fiji | 153.22 (100.47,220.7) | 198.52 (133.04,281.47) | 0.81 (0.77,0.86) | 176.53 (113.48,256.71) | 223.97 (149.01,318.97) | 0.83 (0.77,0.88) | 108.84 (70.34,158.23) | 135.87 (89.37,196.31) | 0.61 (0.53,0.69) |
| Finland | 2167.89 (1521.6,2935.75) | 2309.38 (1623.77,3129.46) | 0.1 (-0.22,0.43) | 2492.74 (1728.12,3411.88) | 2654.14 (1846.83,3636.65) | 0.1 (-0.21,0.41) | 1383.89 (961.83,1898.1) | 1758.47 (1217.7,2412.31) | 0.7 (0.34,1.07) |
| France | 2506.54 (1777.85,3372.98) | 2347.37 (1667.78,3170.63) | -0.13 (-0.19,-0.07) | 3074.41 (2157.2,4188.2) | 2910.15 (2027.13,3953.02) | -0.12 (-0.18,-0.07) | 1290.07 (900.04,1753.48) | 1458.69 (1022.47,1970.18) | 0.57 (0.47,0.67) |
| Gabon | 268.38 (173.35,390.59) | 335.48 (222.14,477.28) | 0.61 (0.49,0.73) | 298.27 (190.78,438.56) | 360.62 (236.2,518.12) | 0.45 (0.34,0.57) | 215.77 (138.32,316.45) | 277.63 (182.84,398.6) | 0.72 (0.6,0.84) |
| Gambia | 243.52 (159.08,353.91) | 346.51 (230.58,491.25) | 1.1 (1.07,1.13) | 298.08 (192.42,437.38) | 426.88 (282.33,608.58) | 1.09 (1.07,1.12) | 166.22 (107.22,242.64) | 232.76 (153.12,336.83) | 1.07 (1.05,1.09) |
| Georgia | 174.46 (113.87,254.04) | 312.95 (208.29,448.37) | 2.91 (2.47,3.34) | 169.02 (107.75,250.79) | 320.36 (211.28,464.76) | 3.03 (2.63,3.43) | 178.57 (117.28,259.2) | 284.45 (186.47,410.32) | 2.57 (2.1,3.04) |
| Germany | 1603.1 (1113.61,2182.16) | 1819.7 (1288.62,2458.41) | 0.6 (0.41,0.8) | 1957.44 (1346.28,2682.62) | 2289.81 (1607.38,3114.43) | 0.73 (0.51,0.95) | 792.04 (550.63,1083.66) | 1135.98 (799.41,1537.54) | 1.36 (1.22,1.5) |
| Ghana | 251.75 (164.7,364.32) | 331.89 (219.38,470.02) | 0.65 (0.58,0.71) | 324.67 (210.81,475.46) | 389.92 (257.33,556.02) | 0.26 (0.17,0.35) | 164.52 (105.44,240.89) | 234.89 (155.1,338.75) | 1.08 (1.03,1.13) |
| Greece | 951.54 (659.63,1310.25) | 588.13 (406.25,810.02) | -1.28 (-1.53,-1.03) | 1263.15 (861.45,1759.19) | 726.67 (492.68,1009.99) | -1.49 (-1.78,-1.2) | 542.42 (372.15,749.26) | 407.29 (281.26,568.37) | -0.72 (-0.83,-0.6) |
| Greenland | 2390.37 (1619.58,3341.81) | 2293.64 (1602.85,3143.4) | -0.2 (-0.29,-0.1) | 2994.56 (2011.44,4216.45) | 2844.01 (1950.77,3962.45) | -0.26 (-0.35,-0.17) | 1366.83 (915.17,1919.26) | 1626.37 (1129.14,2242.62) | 0.74 (0.62,0.86) |
| Grenada | 174.23 (116.14,247.59) | 328.4 (223.23,462.25) | 1.83 (1.69,1.98) | 160.17 (104.79,231.28) | 309.17 (207.35,436.7) | 2.1 (2.02,2.18) | 193.4 (127.61,276.83) | 345.33 (231.77,488.85) | 1.49 (1.04,1.95) |
| Guam | 254.42 (168.1,364.65) | 239.31 (159.99,339.02) | -0.1 (-0.23,0.04) | 305.17 (199,441.39) | 276.09 (182.53,394.96) | -0.2 (-0.31,-0.08) | 192.58 (125.28,279.68) | 175.95 (115.72,253.83) | -0.17 (-0.28,-0.05) |
| Guatemala | 354.55 (236.47,505.99) | 411.4 (275.87,581.76) | 0.11 (-0.05,0.26) | 411.44 (272.21,588.44) | 442.7 (295.94,628.4) | -0.13 (-0.25,0) | 280.3 (184.49,405.34) | 369.33 (246.57,526.07) | 0.53 (0.33,0.73) |
| Guinea | 186.38 (119.17,272.46) | 226.37 (147.3,325.7) | 0.51 (0.43,0.59) | 245.69 (156.94,361.92) | 281.47 (182.03,408.16) | 0.24 (0.18,0.3) | 129.17 (81.74,190.51) | 168.88 (108.87,245.73) | 0.84 (0.74,0.94) |
| Guinea-Bissau | 209.29 (134.18,306) | 264.91 (175.08,376.21) | 0.68 (0.65,0.72) | 242.18 (154.53,358.3) | 306.16 (201.09,439.77) | 0.64 (0.6,0.68) | 168.15 (106.74,246.18) | 197.77 (129.37,284.97) | 0.47 (0.43,0.51) |
| Guyana | 341.26 (228.7,482.97) | 402.71 (272.02,565.42) | 0.15 (0.01,0.28) | 398.94 (265.22,566.08) | 473.23 (317.73,667.23) | 0.18 (0.04,0.33) | 271.39 (179.46,389.29) | 307.35 (205.24,435.64) | 0.02 (-0.12,0.17) |
| Haiti | 173.45 (111.47,254.25) | 177.6 (118.08,253.33) | 0.05 (-0.03,0.12) | 215.94 (136.4,321.35) | 221.56 (145.28,317.09) | 0.07 (-0.06,0.2) | 108.49 (69.78,160.01) | 131.88 (86.87,189.44) | 0.67 (0.61,0.72) |
| Honduras | 140.92 (86.61,214.25) | 197.62 (127.14,290.42) | 1.02 (0.88,1.17) | 167.24 (101.01,257.34) | 225.73 (142.6,337.61) | 0.97 (0.73,1.2) | 105.94 (65.77,159.49) | 163.71 (105.79,239.84) | 1.36 (1.3,1.42) |
| Hungary | 2690.92 (1843.08,3755.4) | 1390.26 (950.48,1936.8) | -2.75 (-2.95,-2.54) | 3248.78 (2214.09,4563.62) | 1498.05 (1010.11,2094.82) | -3.14 (-3.35,-2.92) | 1510.76 (1010.77,2153.57) | 1094.96 (738.57,1550.43) | -1.57 (-1.77,-1.37) |
| Iceland | 1358.78 (945.21,1858.44) | 1402.69 (999.45,1878.78) | 0.12 (0.09,0.15) | 1766.41 (1211.74,2439.44) | 1697.6 (1191.13,2303.65) | -0.18 (-0.22,-0.15) | 793.31 (547.94,1094.97) | 1005.9 (707.54,1361.81) | 0.91 (0.8,1.03) |
| India | 950.99 (557.23,1518.17) | 1124.89 (693.79,1712.09) | 0.42 (0.36,0.47) | 1396.53 (818.42,2224.83) | 1572.97 (964.46,2397.57) | 0.22 (0.15,0.29) | 488.41 (285.06,775.32) | 566.37 (347.5,865.23) | 0.33 (0.27,0.38) |
| Indonesia | 405.08 (237.15,644.08) | 465.34 (286.83,708.16) | 0.14 (0.04,0.25) | 604.79 (353.81,965.06) | 637.63 (392.82,969.98) | -0.19 (-0.32,-0.06) | 159.97 (92,255.24) | 229.22 (139.63,351.21) | 1.01 (0.96,1.07) |
| Iran (Islamic Republic of) | 241.48 (141.31,383.66) | 278.95 (171.67,424.61) | 0.34 (0.27,0.41) | 301.79 (177.24,479.05) | 364.79 (225.79,554.26) | 0.59 (0.53,0.65) | 167.41 (96.62,267.65) | 201.1 (122.16,307.92) | 0.38 (0.31,0.44) |
| Iraq | 125.91 (79.5,186.02) | 148.82 (97.29,215.85) | 0.5 (0.45,0.55) | 156.9 (98.04,232.92) | 165.85 (106.64,244.09) | 0.11 (0.05,0.18) | 88.25 (55.53,130.56) | 124.2 (80.4,181.61) | 1.11 (1.05,1.16) |
| Ireland | 1339.19 (935.79,1817.9) | 1268.87 (899.89,1702.43) | -0.32 (-0.48,-0.16) | 1787.21 (1243.11,2437.42) | 1658.15 (1155.5,2244.31) | -0.39 (-0.51,-0.28) | 599.68 (416.83,821.81) | 750.4 (522.48,1027.29) | 0.66 (0.41,0.92) |
| Israel | 855.09 (592.81,1163.26) | 848.44 (597.83,1137.65) | -0.12 (-0.28,0.04) | 1247.52 (857.39,1701.12) | 1086.16 (755.76,1472.29) | -0.6 (-0.75,-0.44) | 385.24 (266.97,531.11) | 519.6 (363.71,705.66) | 1.06 (0.9,1.21) |
| Italy | 2465.65 (1490.78,3806.35) | 1538.57 (992.55,2230.54) | -1.7 (-1.86,-1.55) | 3093.12 (1866.99,4802.2) | 1861.03 (1198.47,2711.66) | -1.85 (-1.98,-1.72) | 1285.39 (775.04,1993.1) | 1059.09 (685.3,1536.83) | -0.7 (-0.89,-0.51) |
| Jamaica | 115.25 (75.94,165.51) | 189.7 (127.86,267.4) | 1.6 (1.42,1.77) | 143.51 (92.81,208.89) | 238.67 (159.27,338.88) | 1.54 (1.34,1.73) | 75.64 (49.68,108.79) | 120.54 (80.42,173.15) | 1.64 (1.46,1.82) |
| Japan | 755.65 (451.75,1175.57) | 669.78 (421.26,994.24) | -0.49 (-0.61,-0.37) | 826.34 (495.04,1284.19) | 654.31 (412.22,972.66) | -0.9 (-0.96,-0.83) | 590.76 (350.4,928.5) | 665.59 (418.96,989.53) | 0.33 (0.1,0.57) |
| Jordan | 168.97 (110.11,244.5) | 201.48 (132.74,287.87) | 0.62 (0.57,0.68) | 207.51 (134.03,302) | 253.16 (165.94,364.17) | 0.57 (0.48,0.65) | 132.57 (85.42,192.54) | 159.18 (105.2,229.11) | 0.82 (0.7,0.94) |
| Kazakhstan | 127.77 (83.31,186.02) | 208.61 (137.54,298.68) | 2.45 (2.05,2.85) | 128.78 (81.58,190.92) | 208.64 (135.65,301.57) | 2.41 (1.97,2.85) | 119.74 (78.62,174.47) | 193.6 (128.02,279.51) | 2.44 (2.09,2.78) |
| Kenya | 233.12 (137.12,371.21) | 316.81 (194.98,481.78) | 0.93 (0.9,0.96) | 291.42 (171.21,464.86) | 370.27 (226.2,563.36) | 0.73 (0.64,0.82) | 171.73 (100.22,272) | 222.54 (136.09,339.45) | 0.68 (0.57,0.79) |
| Kiribati | 102.55 (64.51,153.04) | 128.24 (83.83,183.66) | 0.67 (0.53,0.81) | 135.53 (85.28,203.64) | 170.64 (110.67,245.49) | 0.69 (0.55,0.84) | 41.18 (25.76,61.4) | 44.93 (29.09,65.33) | 0.14 (0.08,0.19) |
| Kuwait | 159.83 (107.38,227.12) | 202.62 (136.86,285.22) | 0.85 (0.72,0.97) | 194.21 (128.53,279.07) | 253.91 (168.44,362.06) | 0.96 (0.77,1.16) | 110.03 (71.75,160.09) | 164.38 (109.35,236.31) | 1.35 (1.18,1.52) |
| Kyrgyzstan | 113.01 (72.88,166.02) | 115.52 (76.78,165.75) | 0.06 (0.02,0.11) | 105.47 (66.72,159.29) | 115.66 (74.54,167.84) | 0.38 (0.31,0.45) | 121.51 (79.29,177.77) | 108.08 (71.91,154.83) | -0.52 (-0.58,-0.45) |
| Lao People's Democratic Republic | 144.75 (92.48,213.07) | 252.53 (166.13,363.26) | 1.95 (1.86,2.05) | 169.55 (107.67,252.33) | 314.38 (205.81,453.75) | 2.15 (2.08,2.22) | 102.21 (64.58,152.26) | 165.96 (108.24,239.81) | 1.69 (1.59,1.8) |
| Latvia | 729.14 (486.81,1043.22) | 492.33 (328.48,700.23) | -1.98 (-2.24,-1.72) | 792.17 (522.54,1131.45) | 460.75 (305.2,656.81) | -2.6 (-2.9,-2.31) | 525.13 (343.45,764.29) | 485.95 (319.66,706.02) | -0.63 (-0.94,-0.32) |
| Lebanon | 212.19 (138.41,306.1) | 276.39 (185.09,394.66) | 0.71 (0.61,0.82) | 281.22 (182.02,408.9) | 358.71 (238.45,517.75) | 0.65 (0.52,0.79) | 117.5 (75.86,172.5) | 177.5 (117.62,255.2) | 1.41 (1.33,1.49) |
| Lesotho | 121.47 (77.33,178.98) | 153.18 (100.86,219.88) | 0.77 (0.74,0.8) | 122.56 (77.43,182.5) | 156.22 (102.51,225.54) | 0.86 (0.81,0.9) | 107.84 (68.34,160.01) | 127.81 (82.8,184.3) | 0.39 (0.31,0.46) |
| Liberia | 207.72 (132.81,300.62) | 278.44 (184.16,401.29) | 0.91 (0.84,0.99) | 272.68 (175.17,400.53) | 358.71 (236.06,520.05) | 0.83 (0.75,0.9) | 153.13 (98.57,224.42) | 197.93 (128.81,288.02) | 0.8 (0.71,0.88) |
| Libya | 173.31 (110.57,253.9) | 229.37 (151.88,326.52) | 1.09 (0.95,1.24) | 219.26 (139.45,325.58) | 291.24 (191.13,418.84) | 1.17 (1.04,1.3) | 126.6 (80.42,185.49) | 164.46 (107.66,236.63) | 0.98 (0.82,1.13) |
| Lithuania | 484.71 (318.58,700.06) | 660.53 (441.58,934.14) | 0.96 (0.8,1.12) | 464.16 (301.76,673.68) | 638.13 (425.45,904.68) | 0.94 (0.85,1.02) | 503.73 (328.96,737.85) | 629.94 (413.38,918.42) | 0.75 (0.39,1.1) |
| Luxembourg | 1762.29 (1219.87,2410.83) | 2032 (1426.74,2724.66) | 0.61 (0.42,0.79) | 2128.94 (1463.79,2935) | 2534.02 (1757.33,3437.91) | 0.72 (0.56,0.88) | 1019.86 (702.62,1408.74) | 1277.49 (896.15,1734.3) | 0.87 (0.67,1.08) |
| Madagascar | 137.58 (88.41,201.29) | 164.64 (108.38,236.59) | 0.35 (0.27,0.42) | 177.19 (112.59,261.78) | 205.6 (134.13,297.63) | 0.26 (0.19,0.32) | 98.49 (62.73,144.74) | 113.56 (74.3,165.43) | 0.2 (0.1,0.3) |
| Malawi | 198.98 (128.72,286.98) | 279.16 (187.22,397.18) | 1.04 (0.98,1.09) | 245.72 (157.66,359.86) | 321.44 (213.36,460.02) | 0.81 (0.69,0.93) | 141.39 (91.52,206.55) | 197.4 (130.66,283.49) | 1.04 (0.98,1.09) |
| Malaysia | 276.97 (177.24,405.86) | 372.68 (246.93,537.8) | 1.14 (1.06,1.23) | 345.81 (222.02,508.23) | 468.74 (308.24,679.06) | 1.25 (1.16,1.33) | 195.04 (123.48,290.16) | 280.38 (184.81,406.02) | 1.21 (1.17,1.25) |
| Maldives | 214.49 (135.84,317.78) | 430.84 (287.89,615.11) | 2.32 (2.19,2.45) | 297.28 (187.01,444.68) | 564.31 (370.39,811) | 2.11 (1.98,2.25) | 178.4 (111.56,265.54) | 306.62 (201.95,444.78) | 1.82 (1.69,1.95) |
| Mali | 266.51 (169.12,392.09) | 329.25 (212.04,477.82) | 0.62 (0.59,0.64) | 374.18 (237.84,553.88) | 467.97 (299.14,683.95) | 0.6 (0.56,0.65) | 155.17 (97.47,231.7) | 200.1 (127.44,292.36) | 0.87 (0.83,0.91) |
| Malta | 1452.63 (1002.48,1992.42) | 1318.8 (923.61,1792.01) | -0.41 (-0.5,-0.32) | 1935.52 (1319.94,2686.46) | 1682.16 (1165.32,2305.96) | -0.59 (-0.68,-0.51) | 727.7 (501.95,1004.18) | 817.79 (570.94,1110.48) | 0.42 (0.31,0.52) |
| Marshall Islands | 212.27 (137.63,309.58) | 270.39 (179.8,384.03) | 0.77 (0.7,0.84) | 250.99 (161.33,370.27) | 380.36 (252.49,541.88) | 1.33 (1.2,1.47) | 134.1 (85.95,195.42) | 167.14 (108.92,243.23) | 0.82 (0.68,0.96) |
| Mauritania | 228.17 (148.12,334.86) | 288.08 (190.94,413.97) | 0.51 (0.42,0.59) | 280.55 (179.79,414.14) | 376.34 (247.09,547.64) | 0.69 (0.62,0.77) | 157.64 (100.9,231.96) | 203.8 (131.53,296.05) | 0.66 (0.58,0.74) |
| Mauritius | 110.63 (73.27,159.15) | 163.78 (109.46,232.71) | 1.97 (1.74,2.2) | 104.21 (67.02,151.04) | 164.7 (109.96,235.74) | 2.23 (2.01,2.46) | 107.95 (70.89,157.08) | 149.02 (98.1,214.59) | 1.64 (1.4,1.89) |
| Mexico | 633.83 (372.25,1007.14) | 501.88 (310.23,762.76) | -0.76 (-0.92,-0.6) | 833.83 (492.1,1320.4) | 610.71 (378.96,927.26) | -0.97 (-1.16,-0.78) | 451.59 (260.6,719.85) | 367.65 (223.78,560.77) | -0.71 (-0.84,-0.59) |
| Micronesia (Federated States of) | 216.28 (141.96,312.34) | 342.71 (229.23,484.56) | 1.63 (1.56,1.7) | 269.61 (174.92,392.19) | 423.32 (283.52,600.79) | 1.58 (1.52,1.64) | 124.9 (79.88,183.06) | 193.25 (126.23,280.13) | 1.49 (1.44,1.54) |
| Monaco | 937.77 (629.93,1314.58) | 1008.52 (695.26,1390.29) | 0.29 (0.18,0.39) | 1175.25 (783.21,1672.96) | 1306.64 (888.63,1817.49) | 0.42 (0.31,0.52) | 520.53 (348.29,729.77) | 603.52 (415.45,828.96) | 0.53 (0.4,0.67) |
| Mongolia | 127.59 (79.77,190.99) | 173.05 (111.78,255.48) | 1.2 (1.1,1.29) | 130.92 (80.98,198.38) | 173.75 (111.11,257.25) | 1.08 (0.98,1.18) | 119.43 (74.47,179.7) | 166.09 (106.1,245.07) | 1.34 (1.22,1.46) |
| Montenegro | 723.84 (472.27,1046.23) | 672.23 (449.13,957.81) | -0.02 (-0.13,0.09) | 720.98 (467.82,1043.55) | 622.22 (412.88,890.43) | -0.21 (-0.34,-0.09) | 703.62 (456.25,1028.62) | 718.14 (472.72,1031.53) | 0.29 (0.16,0.42) |
| Morocco | 239.14 (147.41,364.02) | 410.51 (266.14,602.63) | 1.73 (1.71,1.74) | 379.33 (229.75,590.64) | 629.37 (399.45,936.29) | 1.62 (1.57,1.68) | 96.64 (61.46,142.17) | 162.9 (106.34,236.07) | 1.67 (1.49,1.85) |
| Mozambique | 190.54 (121.66,279.19) | 273.77 (179.42,392.03) | 1.07 (1.01,1.12) | 228.32 (145.17,336.83) | 301.94 (197.04,433.98) | 0.73 (0.63,0.82) | 139.87 (88.07,205.24) | 207.35 (135.31,300.04) | 1.27 (1.21,1.33) |
| Myanmar | 261.85 (169.3,380.69) | 466.37 (311.61,662.87) | 1.99 (1.9,2.09) | 300.66 (194.61,442.92) | 537.38 (357.59,766.28) | 2.02 (1.93,2.11) | 199 (126.1,293.39) | 331.47 (217.09,480.51) | 1.74 (1.64,1.84) |
| Namibia | 117.24 (75.15,171.4) | 164.94 (108.5,235.38) | 0.96 (0.87,1.06) | 125.74 (80.24,185.57) | 171.94 (111.68,247.89) | 0.85 (0.74,0.96) | 97.75 (62.1,143.6) | 142.47 (92.96,206.76) | 1.12 (1.05,1.2) |
| Nauru | 222.94 (144.87,322.08) | 391.55 (257.59,559.49) | 1.8 (1.73,1.86) | 338 (220.4,485.79) | 493.97 (325.65,707.58) | 1.19 (1.12,1.26) | 165.99 (106.98,243.96) | 249.05 (161.57,362.12) | 1.24 (1.15,1.32) |
| Nepal | 391.06 (238.38,603.39) | 595.58 (375.29,889.11) | 1.28 (1.21,1.35) | 570.88 (336.61,905.92) | 804.56 (494.26,1244.2) | 1.01 (0.93,1.08) | 211.12 (132.19,316.18) | 358.68 (233.72,525.17) | 1.75 (1.72,1.78) |
| Netherlands | 1639.23 (1161.22,2197.97) | 2769.52 (1973.38,3676.54) | 2.56 (1.67,3.45) | 2053.35 (1440.12,2787.76) | 3608.82 (2551.9,4859.05) | 2.71 (1.78,3.65) | 824.63 (581.56,1119.24) | 1536.1 (1085.52,2069.23) | 2.93 (2.1,3.76) |
| New Zealand | 2206.35 (1335.53,3422.81) | 2051.05 (1331.11,2960.47) | 0.33 (0.15,0.52) | 2728.68 (1646.17,4250.19) | 2509.44 (1616.29,3647.28) | 0.35 (0.15,0.54) | 1223.26 (740.37,1887.64) | 1440.51 (933.22,2087.96) | 0.96 (0.73,1.19) |
| Nicaragua | 303.46 (199.52,437.75) | 566.3 (380.57,794) | 2.07 (1.89,2.26) | 341.42 (222.52,499.3) | 715.73 (477.15,1008.87) | 2.47 (2.22,2.71) | 237.3 (154.91,341.67) | 332.2 (222.4,470.12) | 1.21 (1.12,1.31) |
| Niger | 250.52 (157.76,373.07) | 319.09 (206.75,460.1) | 0.85 (0.78,0.93) | 334.38 (209.87,501.94) | 433.88 (279.28,630.64) | 0.83 (0.75,0.91) | 150.52 (92.91,226.63) | 196.42 (125.37,287.35) | 0.99 (0.9,1.09) |
| Nigeria | 255.37 (150.55,406.09) | 339.42 (209.14,516.97) | 0.99 (0.92,1.05) | 321.74 (189.31,511.55) | 458.78 (282.48,699.86) | 1.3 (1.22,1.38) | 160.64 (93.52,255.33) | 211.11 (128.98,323.13) | 0.99 (0.89,1.09) |
| Niue | 248.42 (161.65,355.51) | 340.39 (225.71,483.4) | 0.92 (0.83,1.01) | 280.11 (181.25,403.74) | 393.34 (259.6,561.2) | 1.05 (0.9,1.2) | 156.29 (100.47,227.33) | 228.46 (149.56,330.83) | 1.22 (1.18,1.26) |
| North Macedonia | 243.53 (155.27,356.72) | 680.89 (457.18,967.65) | 4.02 (3.42,4.62) | 278.78 (176.69,410.82) | 834.86 (558.11,1197.71) | 4.21 (3.58,4.86) | 195.94 (124.33,292.04) | 440.83 (290.14,633.15) | 3.2 (2.75,3.65) |
| Northern Mariana Islands | 385.41 (249.34,568.53) | 607.92 (408.21,866.84) | 1.53 (1.43,1.62) | 432.74 (271.99,654.4) | 750.93 (497.65,1079.05) | 1.95 (1.69,2.2) | 317.57 (204.45,461.04) | 406.7 (268.6,585.33) | 0.63 (0.56,0.7) |
| Norway | 2508.99 (1526.09,3856.07) | 2830.99 (1821.79,4133.03) | 0.41 (0.29,0.52) | 3053.17 (1853.65,4696.09) | 3530.51 (2260.8,5177.35) | 0.5 (0.42,0.58) | 1545.6 (940.79,2386.84) | 1835.45 (1181.69,2670.6) | 0.53 (0.38,0.68) |
| Oman | 458.9 (294.53,669.13) | 732.93 (491.26,1044) | 1.35 (1.26,1.44) | 524.81 (334.54,777.07) | 866.9 (573.04,1245.32) | 1.53 (1.46,1.6) | 317.76 (202.13,462.98) | 510.31 (333.37,738.01) | 1.3 (1.19,1.41) |
| Pakistan | 131.4 (76.77,209.36) | 150.75 (91.7,231.33) | 0.41 (0.27,0.55) | 150.4 (87.58,241) | 164.31 (99.64,252.51) | 0.26 (0.13,0.39) | 118.09 (68.13,187.27) | 138.3 (84.04,212.63) | 0.47 (0.32,0.61) |
| Palau | 644.73 (420.12,929.63) | 700.32 (472.19,985.6) | 0.24 (0.18,0.31) | 832.6 (540.6,1205.68) | 992.91 (668.2,1404.34) | 0.55 (0.53,0.58) | 386.17 (247.02,568.62) | 465.33 (304.53,669.02) | 0.57 (0.52,0.62) |
| Palestine | 239.61 (154.98,349.51) | 325.21 (213.9,465.37) | 1.06 (0.92,1.2) | 283.01 (182.46,414.77) | 372.69 (241.77,536.64) | 0.94 (0.78,1.1) | 179.59 (113.71,265.59) | 250.6 (163.99,359.07) | 1.14 (0.99,1.29) |
| Panama | 303.98 (204.19,430.17) | 211.41 (141.31,298.17) | -1.56 (-1.74,-1.38) | 391.57 (259.66,557.6) | 233.53 (154.25,334.81) | -2.08 (-2.29,-1.86) | 201.12 (133.07,288.85) | 179.51 (119.7,257.73) | -0.71 (-0.84,-0.59) |
| Papua New Guinea | 447.51 (278.28,682.22) | 638.73 (410.55,935.45) | 0.96 (0.86,1.06) | 853.51 (528.63,1305.94) | 1287.49 (824.45,1893.03) | 1.17 (1.08,1.25) | 41.43 (26.18,61.12) | 50.7 (32.84,73.57) | 0.55 (0.48,0.61) |
| Paraguay | 243.63 (159.9,351.53) | 325.47 (216.53,465.5) | 1.24 (1.13,1.35) | 274.58 (179.4,401.63) | 380.77 (250.94,548.65) | 1.44 (1.31,1.57) | 196.67 (126.28,286.98) | 240.38 (158.5,347.43) | 0.84 (0.76,0.92) |
| Peru | 128.97 (84,186.45) | 196.8 (129.76,282.85) | 1.27 (1.2,1.34) | 148.34 (95.52,216.59) | 221.62 (144.11,321.48) | 1.19 (1.1,1.28) | 105.88 (67.94,154.92) | 165.98 (107.32,242.13) | 1.42 (1.36,1.48) |
| Philippines | 230.48 (135.34,365.12) | 258.77 (158.04,393.98) | 0.37 (0.19,0.55) | 321.67 (189.3,507.9) | 324.18 (198.49,492.91) | 0.06 (-0.16,0.29) | 149.11 (85.61,237.32) | 161.63 (97.1,249.83) | 0.26 (0.13,0.38) |
| Poland | 1012.26 (598.64,1606.16) | 875.17 (542.19,1332.09) | -0.77 (-0.92,-0.62) | 1196.11 (708.62,1899.82) | 909 (565.22,1382.26) | -1.25 (-1.4,-1.1) | 594.49 (346.34,947.79) | 729.49 (444.67,1119.41) | 0.54 (0.36,0.72) |
| Portugal | 777.82 (542.02,1059.29) | 891.11 (620.75,1214.98) | 0.72 (0.59,0.86) | 953.4 (655.45,1313.75) | 1064.39 (729.65,1473.35) | 0.64 (0.5,0.78) | 465.28 (319.04,644.03) | 625.57 (436.13,854.81) | 1.25 (1.14,1.36) |
| Puerto Rico | 331.12 (221.96,462.19) | 461.77 (309.98,652.04) | 1.32 (1.23,1.41) | 390.16 (259.88,551.28) | 479.62 (317.57,684.21) | 0.86 (0.79,0.94) | 246.93 (164.34,353.67) | 426.26 (285.99,612.04) | 2.06 (1.93,2.19) |
| Qatar | 222.34 (142.07,324.22) | 445.72 (297.24,626.67) | 2.48 (2.35,2.61) | 218.96 (137.66,323.92) | 498.83 (329.25,708.23) | 3.01 (2.88,3.14) | 207.88 (133.26,304.72) | 381.95 (252.1,543.99) | 1.97 (1.85,2.09) |
| Republic of Korea | 751.61 (493.22,1079.97) | 1618.84 (1128.73,2215.99) | 2.24 (1.74,2.74) | 811.87 (527.71,1174.07) | 1876.44 (1293.74,2586.82) | 2.4 (1.87,2.92) | 582.09 (377.88,840.26) | 1064.79 (732.01,1468.82) | 1.91 (1.52,2.3) |
| Republic of Moldova | 348.08 (227.81,506.23) | 254.53 (168.62,369.95) | -0.93 (-1.07,-0.79) | 375.75 (243.41,549.24) | 220.82 (144.85,323.17) | -1.81 (-2.03,-1.58) | 294.68 (193.64,430.78) | 295.85 (193.33,432.42) | 0.3 (0.17,0.44) |
| Romania | 297.67 (197.04,431.1) | 362.77 (237.97,525.68) | 0.75 (0.68,0.82) | 273.55 (178.07,402.83) | 302.89 (195.67,443.76) | 0.42 (0.32,0.53) | 325.05 (212.72,476.99) | 436.76 (289.16,636.88) | 1.08 (1.02,1.14) |
| Russian Federation | 281.3 (162.75,449.55) | 365.01 (220.56,563.25) | 0.98 (0.79,1.16) | 275.98 (159.99,443.28) | 343.71 (206.7,530.33) | 0.82 (0.72,0.92) | 255.44 (146.04,410.46) | 370.33 (222.86,571.99) | 1.41 (1.04,1.78) |
| Rwanda | 185.18 (117.91,271.2) | 272.42 (180.2,385.28) | 1.24 (1.12,1.36) | 222.76 (141.22,331.57) | 319.92 (209.25,455.73) | 1.1 (0.99,1.22) | 129.84 (82.49,190.73) | 182.28 (120.49,263.46) | 1.1 (1,1.2) |
| Saint Kitts and Nevis | 233.06 (157.62,327.42) | 368.58 (249.27,512.67) | 1.5 (1.38,1.63) | 238.34 (159.37,338.06) | 365.34 (244.51,511.84) | 1.42 (1.29,1.55) | 219.1 (147.2,311.18) | 365.68 (246.86,513.26) | 1.64 (1.5,1.79) |
| Saint Lucia | 172.05 (116.84,242.62) | 218.88 (148.45,303.26) | 0.43 (0.28,0.58) | 174.45 (116.39,250.52) | 220.09 (146.78,309.78) | 0.38 (0.21,0.55) | 166.68 (112.52,236.36) | 214.03 (145.3,301.88) | 0.52 (0.4,0.64) |
| Saint Vincent and the Grenadines | 174.02 (117.08,247.99) | 267.55 (182.46,376.59) | 1.3 (1.23,1.36) | 130.98 (86.12,191.02) | 193.45 (128.35,276.63) | 1.34 (1.26,1.41) | 254.55 (171.28,362.87) | 358.95 (244.17,504.54) | 1.02 (0.95,1.08) |
| Samoa | 235.79 (155.34,339.09) | 334.83 (223.48,474.68) | 1.15 (1.12,1.19) | 274.82 (179.55,398.61) | 434.1 (288.16,616.88) | 1.48 (1.39,1.57) | 150.99 (96.78,219.47) | 199.9 (130.61,287.11) | 0.86 (0.82,0.89) |
| San Marino | 988.77 (671.46,1382.33) | 1121.75 (773.41,1550.17) | 0.62 (0.51,0.73) | 1185.73 (791.52,1683.6) | 1406.75 (951.9,1965.77) | 0.8 (0.66,0.93) | 693.45 (467.24,968.65) | 788.01 (542.31,1085.48) | 0.56 (0.51,0.62) |
| Sao Tome and Principe | 295.48 (191.64,428.55) | 511.64 (338.12,734.27) | 1.82 (1.74,1.89) | 267.16 (172.77,390.56) | 439.56 (288.81,631.83) | 1.59 (1.55,1.64) | 326.82 (209.58,478.01) | 589.16 (386.26,857.4) | 2.02 (1.9,2.14) |
| Saudi Arabia | 593.03 (378.41,873.97) | 839.14 (551.09,1212.09) | 0.97 (0.89,1.05) | 713.83 (450.38,1061.71) | 957.22 (619.45,1390.84) | 0.72 (0.65,0.79) | 471.28 (297.98,700.79) | 745.76 (485.73,1083.95) | 1.34 (1.19,1.5) |
| Senegal | 228.06 (147.35,331.17) | 315.27 (209.3,450.46) | 0.89 (0.84,0.94) | 298.81 (192.66,437.65) | 405.67 (268.11,584.74) | 0.83 (0.78,0.89) | 153.26 (98.71,223.12) | 206.07 (135.81,298.45) | 0.81 (0.74,0.87) |
| Serbia | 435.72 (283.16,632.33) | 594.42 (397.32,844.37) | 1.11 (1.03,1.19) | 499.11 (320.71,727.24) | 656.78 (431.79,930.1) | 1.04 (0.96,1.12) | 324.46 (209.18,473.51) | 476.92 (315.4,685.13) | 1.4 (1.26,1.54) |
| Seychelles | 180.49 (116.27,261.83) | 202.06 (134.1,288.75) | 0.46 (0.39,0.52) | 148.72 (96.02,217.35) | 174.25 (114.86,249.53) | 0.65 (0.57,0.74) | 215.51 (137.93,316.94) | 222.46 (146.31,320.02) | 0.12 (0.07,0.17) |
| Sierra Leone | 205.82 (133.18,299.5) | 251.93 (167.07,363.19) | 0.52 (0.47,0.56) | 256.4 (164.4,376.16) | 313.37 (204.64,454.59) | 0.46 (0.4,0.51) | 154.08 (99.18,226) | 182.37 (119.5,263.99) | 0.47 (0.42,0.53) |
| Singapore | 419.33 (286.27,587.22) | 547.25 (378.06,751.28) | 0.55 (0.14,0.96) | 434.4 (287.76,618.53) | 585.7 (391.01,818.64) | 0.52 (0.05,1) | 397.25 (271.84,554.88) | 497.87 (344.96,682.28) | 0.65 (0.36,0.93) |
| Slovakia | 1261.37 (831.12,1818.06) | 1146.99 (769.78,1615.43) | 0.11 (-0.27,0.49) | 1453.06 (950.23,2112.19) | 1196.16 (800.76,1694.02) | -0.12 (-0.63,0.39) | 875.61 (561.86,1279.33) | 958.41 (631.43,1379.63) | 0.44 (0.31,0.57) |
| Slovenia | 2086.18 (1399.26,2949.36) | 2124.47 (1448.64,2930.28) | 0.61 (0.39,0.83) | 2440.14 (1618.1,3460.45) | 2325.14 (1562.57,3239.78) | 0.41 (0.2,0.62) | 1366.06 (898.15,1960.05) | 1659.79 (1114.19,2340.73) | 1.1 (0.89,1.31) |
| Solomon Islands | 494.44 (304.01,750.12) | 769.53 (497.36,1127.23) | 1.42 (1.37,1.48) | 995.82 (607.03,1523.34) | 1368.85 (878.01,2020.59) | 0.95 (0.9,1) | 108.79 (68.82,160.18) | 169.61 (109.07,248.5) | 1.42 (1.39,1.45) |
| Somalia | 134.02 (85.1,197.18) | 157.34 (102.32,226.6) | 0.5 (0.48,0.53) | 159.35 (99.96,236.51) | 182.32 (116.98,263.89) | 0.4 (0.37,0.43) | 102.22 (64.65,150.26) | 107.67 (69.99,157.72) | 0.16 (0.14,0.19) |
| South Africa | 107.47 (63.16,171.31) | 105.45 (64.4,161.1) | -0.43 (-0.56,-0.31) | 117.58 (69.04,187.68) | 110.43 (67.29,169.4) | -0.61 (-0.74,-0.49) | 87.93 (50.83,140.13) | 91.27 (55.34,140.44) | -0.17 (-0.3,-0.04) |
| South Sudan | 146.04 (94.92,213.36) | 169.36 (111.21,242.62) | 0.4 (0.36,0.44) | 182.02 (116.8,269.14) | 218.09 (142.12,316.77) | 0.5 (0.46,0.54) | 110.3 (70.52,162.03) | 125.89 (82.07,182) | 0.38 (0.34,0.43) |
| Spain | 656.26 (449.84,914.33) | 1008.46 (708.28,1373.07) | 1.64 (1.48,1.8) | 803.74 (541.8,1124.28) | 1271.91 (873.93,1751.44) | 1.76 (1.6,1.92) | 413.07 (283.36,577.07) | 615.14 (425.61,843.58) | 1.48 (1.33,1.63) |
| Sri Lanka | 347.63 (228.76,497.3) | 696.24 (468.13,984.45) | 2.35 (2.2,2.5) | 454.56 (300.29,650.83) | 826.84 (554.74,1176.05) | 2.04 (1.93,2.15) | 243.44 (155.84,355.96) | 457.67 (301.73,658.77) | 2.13 (1.94,2.32) |
| Sudan | 121.63 (77.87,179.14) | 172.65 (114.5,247.31) | 1.04 (1,1.08) | 148.19 (93.82,221.31) | 221.31 (144.47,318.03) | 1.27 (1.22,1.32) | 94.02 (59.89,139) | 132.73 (86.71,192.3) | 0.99 (0.94,1.03) |
| Suriname | 185.02 (120.02,266.67) | 257.18 (172.33,362.12) | 1.07 (1,1.14) | 227.56 (146.26,331.95) | 310.01 (205.3,441.14) | 1 (0.94,1.06) | 130.59 (84.87,188) | 177.57 (117.67,252.88) | 0.93 (0.82,1.04) |
| Sweden | 1860.14 (1123.57,2868.65) | 2041.55 (1301.35,2990.3) | 0.47 (0.34,0.6) | 2248.12 (1348.08,3478.27) | 2440.01 (1557.52,3591.57) | 0.46 (0.34,0.58) | 1184.49 (702.62,1836.39) | 1495.35 (949.69,2212.42) | 0.87 (0.7,1.04) |
| Switzerland | 3185.27 (2239.46,4328.26) | 2681.72 (1899.32,3575.84) | -0.31 (-0.53,-0.1) | 3953.97 (2751.92,5385.99) | 3389.7 (2367.5,4584.19) | -0.25 (-0.49,0) | 1705.43 (1185.54,2333.13) | 1650.17 (1170.47,2217.77) | 0.09 (-0.04,0.21) |
| Syrian Arab Republic | 154.76 (100.81,224.38) | 228.81 (151.21,325.54) | 1.18 (1.12,1.25) | 237.19 (153.4,346.62) | 375.04 (247.82,530.09) | 1.43 (1.28,1.59) | 83.85 (53.95,123.57) | 137.9 (90.4,200.01) | 1.5 (1.33,1.67) |
| Taiwan (Province of China) | 348.87 (232.03,501.94) | 299.1 (198.77,425.31) | -0.63 (-0.7,-0.56) | 396.21 (258.29,572.8) | 299.9 (198.64,427.82) | -0.98 (-1.04,-0.93) | 292.56 (194.78,425.04) | 294.14 (194.81,421.58) | -0.16 (-0.26,-0.07) |
| Tajikistan | 93.45 (59.46,139.16) | 114.81 (75.47,166.32) | 0.57 (0.52,0.62) | 88.08 (54.79,133.25) | 114.54 (74.19,167.21) | 0.76 (0.7,0.82) | 99.13 (63.1,147.51) | 114.66 (75.02,167.21) | 0.45 (0.38,0.52) |
| Thailand | 274.08 (177.94,395.8) | 309.12 (201.55,447.77) | 0.16 (-0.03,0.34) | 335.79 (217.36,488.75) | 359.26 (232.19,524.17) | -0.06 (-0.3,0.19) | 175.03 (112.97,255.86) | 235.37 (152.04,345.73) | 0.83 (0.78,0.88) |
| Timor-Leste | 142.93 (91.71,209.71) | 262.5 (172.38,376.12) | 2.32 (2.19,2.45) | 187.2 (119.01,277.33) | 337.07 (222.03,483.12) | 2.2 (2.08,2.32) | 93.95 (59.21,140.04) | 183.67 (118.6,269.47) | 2.56 (2.42,2.7) |
| Togo | 239.52 (155.95,346.6) | 292.35 (194,415.02) | 0.53 (0.44,0.63) | 293 (191.03,424.71) | 329.7 (218.25,474.45) | 0.24 (0.12,0.36) | 173.46 (110.5,253.92) | 216.35 (141.86,312.57) | 0.61 (0.57,0.64) |
| Tokelau | 196.07 (127.63,282.83) | 322.07 (215.48,457.27) | 1.64 (1.62,1.67) | 268.39 (173.36,392.36) | 459.45 (306.5,655.65) | 1.85 (1.79,1.9) | 106.08 (67.79,154.52) | 160.23 (105.28,231.11) | 1.32 (1.28,1.37) |
| Tonga | 201.06 (131.95,289.01) | 249.47 (166.66,352.58) | 0.68 (0.63,0.72) | 302.24 (196.71,437.37) | 365.43 (241.3,518.33) | 0.6 (0.57,0.63) | 58.53 (37.45,85.73) | 74.38 (48.53,108.1) | 0.58 (0.51,0.65) |
| Trinidad and Tobago | 232.13 (158.4,326.17) | 162.69 (108.89,228.54) | -1.12 (-1.27,-0.96) | 280.49 (188.82,397.42) | 183.02 (120.81,260.98) | -1.38 (-1.55,-1.21) | 147.5 (98.11,210.83) | 129.52 (86.26,183.56) | -0.33 (-0.4,-0.26) |
| Tunisia | 175.46 (113.95,254.25) | 259.56 (170.86,370.84) | 1.31 (1.21,1.41) | 210.46 (134.05,308.31) | 303.99 (198.35,437.59) | 1.24 (1.14,1.33) | 134.11 (86.15,195.15) | 199.08 (131.08,287.85) | 1.27 (1.15,1.39) |
| Turkey | 186.62 (120.77,271.63) | 538.46 (358.12,761.57) | 4.4 (4.02,4.78) | 234.51 (151.39,342.74) | 707.03 (467.96,1004.36) | 4.6 (4.23,4.98) | 107.88 (68.53,159.46) | 296.71 (193.67,428.52) | 4.15 (3.8,4.5) |
| Turkmenistan | 83.3 (53.86,121.77) | 82.85 (54.28,119.77) | 0.03 (-0.12,0.17) | 89.47 (56.4,132.97) | 87.66 (56.04,128.5) | 0.02 (-0.15,0.19) | 70.31 (45.68,103.67) | 74.77 (49.12,107.49) | 0.21 (0.11,0.31) |
| Tuvalu | 198.57 (128.88,288.55) | 304.61 (203.53,433.23) | 1.26 (1.2,1.33) | 232.34 (150.46,340.04) | 388.29 (259.37,551.41) | 1.62 (1.5,1.73) | 120.11 (77.23,175.76) | 176.59 (114.57,257.52) | 1.14 (1.1,1.18) |
| Uganda | 189.9 (122.88,272.36) | 271.97 (180.29,386.53) | 1.19 (1.11,1.26) | 224.57 (144.34,327.87) | 316.9 (209.07,453.71) | 1.16 (1.06,1.26) | 143.43 (91.63,209.36) | 186.75 (123.51,269.08) | 0.87 (0.81,0.94) |
| Ukraine | 318.31 (181.08,515.76) | 256.93 (152.88,402.92) | -1.05 (-1.32,-0.78) | 297.76 (168.39,483.5) | 212.01 (125.53,333.9) | -1.42 (-1.6,-1.24) | 335.88 (190.25,544.87) | 315.84 (185.73,496.41) | -0.61 (-1,-0.22) |
| United Arab Emirates | 184.28 (117.13,272.67) | 145.45 (94.15,212.62) | -1.24 (-1.68,-0.81) | 235.39 (147.22,351.86) | 203.98 (130.84,296.09) | -0.79 (-1.28,-0.29) | 130.56 (81.75,194.8) | 139.02 (89.42,204.21) | -0.22 (-0.39,-0.06) |
| United Kingdom | 1082.85 (687.97,1614.49) | 1437.87 (943.66,2056.31) | 1.21 (1.07,1.34) | 1358.62 (861.03,2032.28) | 1846.41 (1208.11,2653.88) | 1.29 (1.15,1.43) | 547.47 (345.62,824.06) | 894.89 (590.36,1283.64) | 1.9 (1.73,2.07) |
| United Republic of Tanzania | 201.53 (130.8,291.62) | 277.54 (183.87,396.43) | 0.98 (0.94,1.02) | 252.89 (162.72,371.1) | 351.44 (230.96,507.51) | 1 (0.93,1.07) | 138 (88.84,200.31) | 180.78 (118.83,261.33) | 0.8 (0.76,0.84) |
| United States of America | 1010.26 (606.05,1566.68) | 1550.45 (968.59,2305.14) | 1.44 (1.17,1.71) | 1180.84 (705.79,1838.35) | 1864.7 (1162.33,2790.44) | 1.53 (1.27,1.8) | 714.6 (430.18,1109.3) | 1143.45 (722.29,1703.88) | 1.63 (1.38,1.88) |
| United States Virgin Islands | 271.74 (179.56,387.92) | 369.3 (245.95,518.68) | 1.06 (1,1.13) | 290.96 (190.7,419.75) | 360.52 (237.01,515.18) | 0.66 (0.56,0.75) | 235.34 (154.44,336.74) | 365.49 (244.65,521.72) | 1.61 (1.52,1.7) |
| Uruguay | 694.06 (477.99,956.5) | 841.74 (588.42,1144.42) | 0.69 (0.61,0.78) | 877.44 (597.5,1218.12) | 1068.7 (739.86,1467.46) | 0.73 (0.64,0.82) | 381.87 (259.18,537) | 427.51 (295.97,590.59) | 0.35 (0.29,0.42) |
| Uzbekistan | 88.54 (57.73,128.08) | 135.58 (90.49,193.66) | 1.67 (1.42,1.92) | 90.81 (57.46,133.82) | 138.04 (90.34,199.19) | 1.69 (1.48,1.9) | 83.07 (54.06,120.62) | 126.02 (83.82,180.42) | 1.55 (1.25,1.85) |
| Vanuatu | 150.92 (97.71,219.88) | 214.04 (141.72,306.68) | 1.14 (1.07,1.2) | 206.99 (133.95,304.11) | 278.01 (183.06,398) | 0.95 (0.86,1.05) | 99.76 (63.73,146.91) | 136.16 (88.39,199.83) | 1.01 (1,1.02) |
| Venezuela (Bolivarian Republic of) | 379.45 (254.51,533.12) | 325.09 (218.34,456.85) | -0.61 (-0.73,-0.49) | 501.41 (333.44,710.04) | 411.36 (274.08,583.93) | -0.71 (-0.86,-0.55) | 223.96 (149.34,319.47) | 194.13 (129.98,276.21) | -0.67 (-0.79,-0.55) |
| Viet Nam | 475.63 (297.86,722.09) | 916.44 (597.3,1324.72) | 2.25 (2.05,2.44) | 585.68 (364.49,895.89) | 1106.07 (718.91,1608.8) | 2.17 (1.95,2.39) | 235.61 (143.42,360.93) | 478.45 (301.78,714.75) | 2.43 (2.29,2.57) |
| Yemen | 128.08 (81.64,188.74) | 160.26 (105.32,231.5) | 0.84 (0.76,0.92) | 142.68 (90.1,212.71) | 193.43 (125.52,280.4) | 1.12 (1.01,1.23) | 94.95 (59.73,139.34) | 122.69 (79.53,178.68) | 1 (0.9,1.09) |
| Zambia | 211.93 (137.12,306.17) | 300.68 (199.73,428.95) | 1.09 (0.96,1.22) | 273.58 (174.6,397.59) | 358.77 (235.31,519.93) | 0.79 (0.63,0.94) | 157.51 (101.18,229.67) | 218.93 (143.71,314.26) | 1.04 (0.99,1.09) |
| Zimbabwe | 222.68 (143.49,322.24) | 226.57 (148.68,324.72) | -0.28 (-0.42,-0.14) | 261.43 (166.41,384.89) | 276.83 (180.43,400.62) | 0.03 (-0.06,0.11) | 149.45 (96.37,218.23) | 146.17 (95.39,212.02) | -0.6 (-0.81,-0.38) |

| **Table S4. The ASPR for 1990 and 2021, and the EAPC of ASPR from 1990 to 2021.** | | | | | | | | | |
| --- | --- | --- | --- | --- | --- | --- | --- | --- | --- |
|  | **Both** | | | **Female** | | | **Male** | | |
|  | **ASPR per 100,000 population, 1990** | **ASPR per 100,000 population, 2021** | **EAPC** | **ASPR per 100,000 population, 1990** | **ASPR per 100,000 population, 2021** | **EAPC** | **ASPR per 100,000 population, 1990** | **ASPR per 100,000 population, 2021** | **EAPC** |
| Global | 1606.23 (1274.92,2019.73) | 1834.14 (1470.71,2274.39) | 0.48 (0.43,0.52) | 2072.51 (1644.05,2604.8) | 2313.46 (1854.56,2870.17) | 0.4 (0.37,0.43) | 828.23 (647.59,1060.12) | 1141.48 (908.43,1426.3) | 1.13 (1.05,1.22) |
| High SDI | 2791.61 (2225.12,3489.53) | 3421.55 (2759.5,4210.12) | 0.77 (0.7,0.85) | 3443.72 (2748.24,4305.32) | 4247.6 (3419.95,5226.59) | 0.8 (0.74,0.85) | 1557.06 (1221.33,1980.81) | 2272.5 (1807.67,2817.1) | 1.39 (1.25,1.53) |
| High-middle SDI | 1352.48 (1062.63,1715.48) | 1477.82 (1180.6,1842.19) | 0.23 (0.18,0.28) | 1639.85 (1285.83,2083.6) | 1785.3 (1427.02,2223.27) | 0.22 (0.18,0.27) | 781.04 (608.63,1003.15) | 957.39 (757.34,1208.55) | 0.62 (0.56,0.68) |
| Middle SDI | 624.65 (474.18,822.57) | 1009.64 (779.57,1304.04) | 1.44 (1.26,1.61) | 820.09 (620.38,1084.12) | 1315.06 (1013.36,1703.52) | 1.44 (1.27,1.6) | 359.05 (273.3,472.19) | 584.18 (450,758.61) | 1.41 (1.22,1.59) |
| Low-middle SDI | 682.03 (505.62,920.24) | 961.67 (731.94,1255.97) | 1.14 (1.04,1.23) | 1005.04 (741.61,1363.32) | 1363.99 (1035.16,1789.32) | 1.02 (0.92,1.12) | 346.67 (259.83,463.39) | 468.36 (359.5,609.11) | 0.92 (0.81,1.03) |
| Low SDI | 459.15 (342.86,612.47) | 693.26 (526.32,905.06) | 1.36 (1.29,1.44) | 656.81 (487.75,880.54) | 984.09 (745.95,1291.49) | 1.34 (1.27,1.42) | 247.75 (185.3,328.72) | 361.55 (275.81,470.2) | 1.24 (1.16,1.31) |
| Andean Latin America | 281.85 (224.94,353.35) | 445.35 (352.94,558.08) | 1.43 (1.31,1.54) | 322.15 (254.4,405.92) | 509.21 (401.79,641.25) | 1.49 (1.36,1.62) | 233.3 (186.15,290.7) | 363.31 (289.12,453.54) | 1.32 (1.21,1.42) |
| Australasia | 3182.39 (2559.01,3910.96) | 4725.35 (3901.08,5685.83) | 1.71 (1.54,1.88) | 3991.67 (3203.16,4918.3) | 5797.13 (4768.78,6974.74) | 1.64 (1.49,1.79) | 1751.34 (1378.81,2193.84) | 3339.09 (2716.67,4061.08) | 2.52 (2.27,2.78) |
| Caribbean | 782.09 (623.54,976.41) | 1273.59 (1022.35,1560.2) | 1.5 (1.36,1.64) | 1115.02 (886.72,1399.76) | 1747.63 (1402.32,2143.05) | 1.36 (1.21,1.5) | 389.15 (308.69,482.99) | 639.19 (510.15,793.29) | 1.63 (1.56,1.7) |
| Central Asia | 247.15 (196.39,310.08) | 284.67 (225.65,357.52) | 1.02 (0.73,1.31) | 273.09 (215.12,344.66) | 324.34 (255.45,409.7) | 1.15 (0.85,1.45) | 191.98 (153.81,237.96) | 212.54 (168.33,265.94) | 0.82 (0.58,1.07) |
| Central Europe | 1718.37 (1343.29,2179.52) | 1478.32 (1166.93,1855.97) | -0.65 (-0.72,-0.58) | 2164.15 (1690.63,2748.9) | 1659.27 (1313.95,2084.47) | -1.05 (-1.12,-0.98) | 861.14 (668.28,1101.86) | 1083.96 (843.76,1378.9) | 0.69 (0.59,0.79) |
| Central Latin America | 857.08 (665.09,1106.21) | 718.23 (564.59,910.26) | -0.5 (-0.61,-0.39) | 1106.23 (855.71,1431.41) | 889.2 (696.57,1128.22) | -0.61 (-0.73,-0.5) | 579.16 (451.29,745.48) | 490.26 (384.57,622.38) | -0.48 (-0.57,-0.39) |
| Central Sub-Saharan Africa | 246.91 (188.45,319.12) | 360.53 (278.59,457.52) | 1.24 (1.1,1.39) | 322.96 (246.18,418.03) | 445.67 (343.02,564.73) | 1.06 (0.88,1.24) | 161.29 (121.68,210.28) | 214.74 (164.98,274.9) | 0.94 (0.8,1.08) |
| East Asia | 571.95 (429.16,763.1) | 1121.95 (860.59,1453.4) | 2.01 (1.56,2.47) | 720.27 (539.59,961.46) | 1392.07 (1070.48,1801.38) | 2.01 (1.57,2.45) | 342.78 (256.74,460.22) | 697.45 (531.69,911.7) | 2.1 (1.67,2.53) |
| Eastern Europe | 595.45 (456.91,779.52) | 689.41 (531.98,893.66) | 0.65 (0.52,0.79) | 627.67 (480.54,823.96) | 701.4 (542.9,912.51) | 0.48 (0.37,0.58) | 469.71 (358.71,614.52) | 618.41 (470.94,806.55) | 1.23 (0.98,1.48) |
| Eastern Sub-Saharan Africa | 263.79 (200.43,346.71) | 386.42 (297.76,498.08) | 1.31 (1.22,1.39) | 353.31 (268.15,465.61) | 505.56 (389.37,652.44) | 1.2 (1.11,1.3) | 161.61 (121.54,213.85) | 227.95 (175.42,294.06) | 1.22 (1.15,1.3) |
| High-income Asia Pacific | 1902.65 (1476.84,2438.37) | 2186.04 (1749.28,2707.11) | 0.33 (0.15,0.51) | 2087.69 (1624.22,2663.58) | 2331.82 (1872.26,2878.86) | 0.21 (0.04,0.38) | 1502.4 (1153.51,1960.75) | 1914.66 (1514.3,2396.57) | 0.75 (0.54,0.96) |
| High-income North America | 2504.15 (1931.91,3225.96) | 4093.01 (3216.69,5163.03) | 1.78 (1.62,1.93) | 3065.23 (2360.4,3952.55) | 5198.4 (4084.58,6568.88) | 1.9 (1.75,2.05) | 1544.35 (1181.68,2016.35) | 2663.69 (2070.01,3380.19) | 2.03 (1.87,2.18) |
| North Africa and Middle East | 300.99 (235.27,384.51) | 558.54 (441.82,700.33) | 2.2 (2.04,2.37) | 403.16 (312.7,520.03) | 778.42 (613.75,975.7) | 2.41 (2.23,2.58) | 185.47 (146.57,233.73) | 330.74 (262.36,414.77) | 1.91 (1.83,1.99) |
| Oceania | 476.2 (364.33,621.36) | 763.84 (596.1,962.09) | 1.4 (1.29,1.5) | 824.38 (627.86,1084.17) | 1377.9 (1070.69,1741.86) | 1.55 (1.43,1.66) | 89.45 (69.4,114) | 116.87 (91.9,147.48) | 0.85 (0.83,0.87) |
| South Asia | 956.01 (701.33,1301.7) | 1418.24 (1073.26,1866.94) | 1.28 (1.2,1.36) | 1491.88 (1090.26,2038.13) | 2082.6 (1570.56,2750.8) | 1.07 (1,1.14) | 445.53 (328.7,602.54) | 638.81 (484.34,839.13) | 1.1 (1,1.2) |
| Southeast Asia | 556.35 (426.16,729.28) | 775.4 (608.43,987.28) | 1 (0.94,1.06) | 769.33 (586.38,1014.18) | 1015.19 (793.45,1293.85) | 0.78 (0.7,0.86) | 255.57 (195.24,333.55) | 407.8 (317.37,523.76) | 1.51 (1.48,1.55) |
| Southern Latin America | 1358.92 (1089.15,1680.59) | 1675.07 (1361.15,2042.89) | 0.74 (0.67,0.81) | 1723.88 (1370.28,2145.96) | 2111.66 (1707,2585.96) | 0.74 (0.66,0.83) | 774.11 (624.92,955.1) | 962.08 (778.08,1180.63) | 0.69 (0.65,0.73) |
| Southern Sub-Saharan Africa | 209.26 (160.86,270.73) | 169.48 (129.11,222.01) | -1.01 (-1.2,-0.83) | 242.82 (186.36,315.66) | 190.18 (144.6,248.94) | -1.16 (-1.37,-0.95) | 147.77 (113.14,192.23) | 125.85 (95.43,165.73) | -0.72 (-0.86,-0.58) |
| Tropical Latin America | 771.62 (588.65,1012.45) | 994.63 (777.05,1270.49) | 0.85 (0.77,0.93) | 936.03 (711.48,1237.39) | 1216.57 (944.6,1556.6) | 0.91 (0.86,0.96) | 538.51 (412.68,703.19) | 653.56 (512.33,834.16) | 0.56 (0.4,0.72) |
| Western Europe | 3626.39 (2922.96,4477.03) | 3961.89 (3252.74,4788.81) | 0.4 (0.34,0.45) | 4566.35 (3677.47,5656.3) | 5070.78 (4156.51,6141.47) | 0.46 (0.4,0.51) | 1805.93 (1436.14,2254.71) | 2384.65 (1939.79,2910.65) | 1.04 (0.94,1.14) |
| Western Sub-Saharan Africa | 345.07 (260.96,454.2) | 471.06 (362.65,606.26) | 1.01 (0.93,1.08) | 459.84 (346.88,607.19) | 639.68 (491.38,823.7) | 1.09 (1.02,1.17) | 200.79 (151.83,265.02) | 276.36 (212.41,357.57) | 1.08 (1.02,1.15) |
| Afghanistan | 140.4 (107.43,181.24) | 197.24 (152.8,251) | 0.97 (0.85,1.1) | 193.6 (146.23,253.85) | 269.63 (205.97,347.84) | 0.93 (0.8,1.05) | 97.24 (74.58,124.47) | 131.54 (102.35,166.07) | 0.93 (0.8,1.06) |
| Albania | 378.8 (293.98,487.41) | 463.18 (361.99,588.69) | 0.72 (0.34,1.11) | 477.23 (368.95,619.64) | 582.58 (452.02,748.79) | 0.77 (0.39,1.16) | 216.54 (166.49,281.86) | 315.55 (244.07,399.13) | 1.32 (0.95,1.69) |
| Algeria | 274.92 (216.66,348.79) | 422.18 (338.11,523.06) | 1.33 (1.22,1.44) | 351.88 (272.71,453.54) | 603.06 (477.82,742.9) | 1.82 (1.74,1.9) | 219.29 (172.72,275.81) | 296.19 (236.01,371.51) | 0.92 (0.85,0.99) |
| American Samoa | 347.18 (270.35,437.82) | 467.65 (366.75,584.75) | 1.06 (1,1.12) | 386.01 (300.06,485.14) | 567.01 (440.77,707.62) | 1.36 (1.31,1.41) | 287.34 (221.3,372.2) | 347.83 (270.21,444.35) | 0.62 (0.42,0.82) |
| Andorra | 5597.5 (4505.7,6817.58) | 7136.9 (5919.09,8514.99) | 0.81 (0.75,0.88) | 8387.64 (6720.92,10264.64) | 10988.09 (9096,13132.92) | 0.85 (0.8,0.89) | 2433.78 (1931.71,3042.27) | 2737.86 (2210.71,3352.23) | 0.26 (0.15,0.38) |
| Angola | 208.83 (159.28,270.05) | 371.91 (286.58,476.69) | 2.01 (1.94,2.08) | 263.19 (199.29,342.59) | 458.66 (351.84,589.69) | 1.98 (1.9,2.06) | 142.96 (107.31,186.49) | 234.7 (179.45,303.01) | 1.7 (1.64,1.75) |
| Antigua and Barbuda | 257.58 (200.94,328.94) | 396.78 (312.01,495.65) | 1.54 (1.42,1.66) | 289.84 (223,374.37) | 486.06 (378.04,613.56) | 1.75 (1.59,1.91) | 200.75 (156.36,256.95) | 268.88 (210.34,338.75) | 1.16 (1.05,1.26) |
| Argentina | 1255.71 (1003.98,1562.28) | 1196.88 (962.41,1479.74) | -0.23 (-0.28,-0.18) | 1623.57 (1285.96,2034.39) | 1534.92 (1223.71,1906.32) | -0.23 (-0.29,-0.17) | 665.48 (533.66,823.69) | 652.19 (520.9,806.58) | -0.25 (-0.3,-0.19) |
| Armenia | 485.13 (382.36,614.43) | 325.34 (253.89,410.14) | -1.09 (-1.64,-0.53) | 580.24 (452.72,744.09) | 407.74 (313.65,517.07) | -0.77 (-1.45,-0.09) | 320.07 (253.68,400.07) | 193.77 (150.92,248.44) | -1.82 (-2.16,-1.48) |
| Australia | 2985.92 (2407.65,3646.47) | 4832.22 (3994.75,5799.61) | 1.95 (1.77,2.12) | 3733.05 (2998.12,4581.87) | 5907.8 (4865.11,7090.73) | 1.87 (1.71,2.02) | 1671.49 (1318.71,2084.78) | 3443.07 (2800.28,4178.97) | 2.75 (2.49,3.01) |
| Austria | 4248.32 (3455.57,5180.34) | 4401.43 (3602.4,5313.96) | 0.51 (0.36,0.65) | 5092.51 (4114.13,6237.22) | 5219.37 (4258.51,6335.12) | 0.52 (0.35,0.7) | 2316.67 (1853.14,2845.09) | 3136.2 (2531.07,3801.12) | 1.27 (1.1,1.44) |
| Azerbaijan | 173.87 (136.64,219.58) | 177.64 (137.04,228.18) | 0.33 (0.09,0.57) | 192.94 (149.93,246.44) | 201.21 (152.15,261.41) | 0.41 (0.16,0.65) | 129.87 (101.32,164.32) | 140.08 (108.34,179.97) | 0.54 (0.34,0.73) |
| Bahamas | 354.99 (279.92,444.02) | 484.98 (383.75,606.28) | 0.86 (0.65,1.07) | 432.54 (338.94,541.91) | 617.67 (484.35,777.25) | 1.04 (0.82,1.26) | 208.83 (162.12,267.9) | 281.4 (221.56,354) | 0.85 (0.74,0.97) |
| Bahrain | 205.33 (158.36,265.84) | 310.77 (243.91,389.71) | 1.43 (1.35,1.51) | 228.77 (174.1,297.55) | 378.01 (293.15,478.53) | 1.71 (1.61,1.8) | 166.85 (126.69,218.27) | 228.51 (178.43,289.22) | 1.1 (1.03,1.17) |
| Bangladesh | 93.76 (72.79,119) | 190.61 (151.15,237.85) | 2.46 (2.1,2.82) | 119.61 (91.47,153.47) | 229.07 (179.24,286.73) | 2.27 (1.8,2.73) | 74.03 (56.7,94.1) | 156 (123.65,197.21) | 2.56 (2.34,2.77) |
| Barbados | 301.51 (236.64,378.87) | 460.35 (361.52,579.19) | 1.37 (1.21,1.53) | 384.65 (298.39,490.37) | 644.89 (501.43,815.47) | 1.63 (1.48,1.78) | 154.88 (121.53,194.86) | 207.95 (163.31,263.14) | 1.04 (0.88,1.2) |
| Belarus | 499.26 (397.41,625.2) | 833.55 (655.04,1050.86) | 1.85 (1.52,2.18) | 530.17 (419.25,667.11) | 858.51 (675.74,1078.62) | 1.64 (1.34,1.93) | 414.25 (327.36,516.54) | 718.58 (553.53,919.73) | 2.25 (1.82,2.68) |
| Belgium | 3979.37 (3212.22,4876.87) | 6028.17 (4960.53,7249.87) | 1.53 (1.34,1.72) | 5231.43 (4205.66,6442.12) | 8133.95 (6651.29,9813.29) | 1.62 (1.45,1.8) | 1673.82 (1338.93,2072.74) | 3141.53 (2548.67,3836.29) | 2.28 (2.03,2.52) |
| Belize | 262.3 (207.87,328.34) | 391.96 (310.45,490.89) | 1.23 (1.08,1.39) | 336.14 (261.4,428.05) | 491.71 (386.21,622.4) | 1.19 (1.03,1.36) | 172.76 (137.91,214.29) | 288.4 (228.81,361.18) | 1.56 (1.39,1.73) |
| Benin | 302.54 (232.63,388.73) | 415.47 (325.24,524.83) | 0.89 (0.83,0.95) | 396.88 (303.58,513.06) | 516.84 (402.42,657.35) | 0.69 (0.62,0.75) | 199.51 (152.38,257.34) | 266.17 (206.33,342.07) | 0.81 (0.75,0.87) |
| Bermuda | 377.81 (296.75,474.56) | 608.56 (484.54,760.21) | 1.34 (1.09,1.59) | 411.53 (320.07,520.58) | 641.78 (506.16,806.83) | 1.21 (0.91,1.52) | 309.68 (240.17,390.91) | 545.57 (429.27,686.33) | 1.69 (1.53,1.84) |
| Bhutan | 433.24 (330.2,565.04) | 1216.49 (938.62,1560.38) | 3.46 (3.2,3.72) | 560.26 (424.45,740.41) | 1726.06 (1324.13,2218.62) | 3.76 (3.42,4.1) | 259.4 (198.7,333.09) | 688.83 (527.95,886.41) | 3.29 (3.08,3.5) |
| Bolivia (Plurinational State of) | 271.68 (210.89,349.91) | 448.88 (349.68,568.66) | 1.68 (1.59,1.76) | 302.97 (232.64,392.78) | 516.92 (398.83,662.06) | 1.82 (1.7,1.94) | 225.29 (174,290.22) | 357.64 (279.86,452.56) | 1.49 (1.45,1.52) |
| Bosnia and Herzegovina | 671.19 (515.65,869.79) | 730.13 (568.73,923.82) | 0.76 (0.37,1.14) | 723.64 (553.48,943.35) | 767.71 (593.48,977.34) | 0.65 (0.27,1.03) | 574.65 (438.34,745.56) | 657.22 (506.05,831.08) | 0.96 (0.6,1.33) |
| Botswana | 220.19 (167.65,283.41) | 259.23 (196.91,337.16) | 0.62 (0.47,0.77) | 254.11 (192.35,330.33) | 291.3 (219.81,382.43) | 0.45 (0.28,0.62) | 157.76 (117.34,205.57) | 187.01 (141.44,243.59) | 0.77 (0.55,0.99) |
| Brazil | 779.93 (594.45,1024.38) | 1003.15 (783.42,1282.21) | 0.84 (0.76,0.92) | 946.62 (718.91,1252.71) | 1226.63 (951.92,1570.65) | 0.9 (0.85,0.95) | 543.12 (415.78,710.04) | 659.27 (516.64,841.78) | 0.56 (0.39,0.72) |
| Brunei Darussalam | 1096.21 (859.85,1389.98) | 1636.36 (1310.84,2025.56) | 1.28 (1.16,1.4) | 1309.49 (1020.41,1669.42) | 1926.96 (1528.04,2391.57) | 1.29 (1.1,1.48) | 845.46 (653.39,1072.1) | 1230.98 (980.15,1525.05) | 1.17 (1.05,1.29) |
| Bulgaria | 488.8 (381.38,619.84) | 544.42 (427.27,690.22) | 0.66 (0.47,0.86) | 516.05 (396.87,663.72) | 592.99 (462.44,755.36) | 0.85 (0.64,1.07) | 447.37 (349.8,561.3) | 453.88 (352.32,579.55) | 0.22 (0.04,0.4) |
| Burkina Faso | 339.66 (259.74,436.58) | 425.99 (332.22,542.8) | 0.71 (0.66,0.77) | 465.81 (354.7,599.43) | 561.85 (438.09,718.55) | 0.58 (0.53,0.64) | 189.2 (143.43,246.89) | 245.54 (189,316.04) | 0.89 (0.83,0.95) |
| Burundi | 259.74 (196.21,341.29) | 313.6 (243.48,398.24) | 0.88 (0.72,1.03) | 335.54 (253.41,441.96) | 410.48 (317.2,526.06) | 0.87 (0.71,1.02) | 159.81 (118.46,211.56) | 203.84 (156.24,258.43) | 1.19 (1.02,1.36) |
| Cabo Verde | 344.44 (269.98,434.95) | 524.19 (410.2,660.71) | 1.56 (1.47,1.66) | 423.03 (329.6,533.95) | 582.9 (454.39,735.42) | 1.23 (1.14,1.32) | 233.95 (183,297.98) | 389.71 (300.7,497.73) | 1.91 (1.76,2.07) |
| Cambodia | 444.39 (340.25,574.59) | 994.35 (770.84,1272.1) | 2.88 (2.77,2.98) | 561.23 (425.87,730.13) | 1223.79 (942.69,1571.73) | 2.79 (2.69,2.88) | 254.03 (194.07,329.11) | 552.83 (426.61,711.63) | 2.84 (2.71,2.97) |
| Cameroon | 355.05 (273.39,458.81) | 456.65 (348.95,589.09) | 0.65 (0.49,0.81) | 466.21 (356.81,606.19) | 574.66 (434.96,745.66) | 0.5 (0.33,0.68) | 230.82 (176.39,298.76) | 297.19 (227.33,384.72) | 0.68 (0.52,0.84) |
| Canada | 3308.79 (2669.16,4049.68) | 4776.77 (3929.47,5756.88) | 1.51 (1.3,1.71) | 4063.8 (3257.01,4986.24) | 5991.48 (4904.6,7235.03) | 1.58 (1.38,1.78) | 2028.36 (1615.8,2523.1) | 3171.73 (2576.83,3874.35) | 1.83 (1.64,2.01) |
| Central African Republic | 202.67 (151.84,266.17) | 215.72 (162.06,282.53) | 0.18 (0.08,0.27) | 244.61 (182.19,321.51) | 254.96 (190.76,335.25) | 0.12 (0.03,0.21) | 133.14 (98.04,177.1) | 138.76 (103,182.15) | 0.11 (0.03,0.19) |
| Chad | 256.28 (196.63,330.03) | 298.07 (231.19,380.31) | 0.5 (0.4,0.59) | 333.63 (252.79,430.57) | 402.4 (310.13,518.18) | 0.58 (0.49,0.67) | 164.09 (124.01,212.6) | 203.76 (155.77,261.46) | 0.73 (0.63,0.83) |
| Chile | 1619.48 (1288.18,1995.68) | 2667.83 (2162.54,3242.8) | 1.95 (1.83,2.06) | 1946.77 (1522.78,2404.52) | 3354.63 (2701.39,4085.97) | 2.12 (2,2.25) | 1119.58 (894.9,1387) | 1609.51 (1297.28,1987.01) | 1.4 (1.3,1.49) |
| China | 577.38 (432.23,771.69) | 1152.4 (883.18,1493.55) | 2.07 (1.6,2.53) | 729.73 (545.64,976.11) | 1432.44 (1100.91,1854.18) | 2.05 (1.61,2.51) | 342.02 (255.25,460.41) | 711.93 (542.16,931.61) | 2.18 (1.73,2.62) |
| Colombia | 591.05 (468.22,744.58) | 489.98 (391.43,609.32) | -0.58 (-0.79,-0.38) | 761.2 (597.18,964.57) | 587.97 (463.34,736.6) | -0.86 (-1.1,-0.63) | 391.5 (311.6,495.54) | 352.63 (279.71,441.07) | -0.22 (-0.36,-0.08) |
| Comoros | 222.11 (169.82,288.51) | 315.61 (246.93,404) | 1.16 (1.03,1.28) | 285.34 (216.77,373.52) | 405.91 (314.98,521.34) | 1.11 (0.98,1.23) | 142.96 (107.69,186.67) | 199.75 (154.93,258.16) | 1.2 (1.06,1.33) |
| Congo | 261.06 (198.1,339.62) | 345.74 (265.52,443.01) | 0.92 (0.77,1.07) | 320.39 (241.07,419.46) | 435.54 (332.14,560.93) | 0.99 (0.83,1.14) | 172.66 (130.47,225.43) | 227.99 (174.23,294.31) | 0.97 (0.81,1.12) |
| Cook Islands | 281.28 (218.12,358.84) | 463.98 (363.57,578.54) | 1.63 (1.52,1.75) | 321.76 (246.61,410.81) | 516.2 (402.13,639.69) | 1.52 (1.34,1.71) | 228.92 (174.61,296.56) | 387.08 (298.47,493.43) | 1.79 (1.72,1.87) |
| Costa Rica | 1188.44 (949.14,1473.21) | 1149.82 (920.88,1415.54) | -0.03 (-0.11,0.06) | 1695.22 (1344.26,2112.76) | 1537.62 (1222.14,1896.06) | -0.25 (-0.34,-0.16) | 621.53 (494.86,778.73) | 626.25 (497.57,781.28) | 0.23 (0.12,0.34) |
| Croatia | 2110.97 (1642.03,2671.26) | 3347.7 (2676.02,4140.67) | 1.68 (1.5,1.87) | 2619.42 (2033.06,3321.18) | 4107.84 (3287.56,5083.12) | 1.7 (1.55,1.86) | 1012.49 (770.29,1306.89) | 1864.57 (1451.03,2388.66) | 1.99 (1.76,2.22) |
| Cuba | 1355.75 (1074.96,1700.13) | 2383.76 (1905.61,2922.61) | 1.67 (1.5,1.84) | 2078.36 (1641.92,2624.15) | 3392.45 (2709.4,4166.54) | 1.45 (1.3,1.61) | 592.45 (465.65,737.31) | 1049 (829.69,1310.73) | 1.8 (1.71,1.9) |
| Cyprus | 3059.71 (2425.69,3789.27) | 3939.73 (3215.4,4762.97) | 1.02 (0.8,1.24) | 4160.58 (3270.84,5176.34) | 5339.91 (4348.97,6463.05) | 0.87 (0.68,1.06) | 1509.62 (1203.68,1873.04) | 2049.25 (1646.47,2514.81) | 1.02 (0.79,1.26) |
| Czechia | 3536.49 (2764.89,4406.29) | 2134.92 (1694.4,2660.13) | -2.12 (-2.27,-1.98) | 4539.35 (3551.64,5667.77) | 2449.05 (1928.71,3055.05) | -2.51 (-2.66,-2.36) | 1338.76 (1030,1726.06) | 1516.19 (1187.53,1919.78) | -0.05 (-0.26,0.16) |
| C?te d'Ivoire | 318.43 (244.87,409.33) | 423.58 (329.29,538.65) | 0.89 (0.74,1.04) | 418.62 (320.45,536.94) | 540.13 (418.12,688.23) | 0.79 (0.61,0.98) | 212 (161.82,276.06) | 282.94 (216.15,367.75) | 0.94 (0.81,1.07) |
| Democratic People's Republic of Korea | 305.46 (235.99,389.88) | 231.93 (181.56,293.64) | -0.09 (-0.38,0.21) | 344.81 (265.06,442.39) | 275.16 (213.06,350.06) | 0.06 (-0.23,0.35) | 206.96 (158.78,267.88) | 126.5 (98.7,160.73) | -0.88 (-1.17,-0.58) |
| Democratic Republic of the Congo | 250.91 (190.74,325.57) | 359.86 (277.77,456.27) | 1.17 (1.01,1.32) | 336.87 (255.76,437.67) | 447.25 (342.64,566.66) | 0.92 (0.71,1.14) | 163.05 (122.22,213.2) | 205.46 (157.25,264.45) | 0.73 (0.57,0.89) |
| Denmark | 5143.56 (4201.55,6229.23) | 3510.64 (2900.88,4207.51) | -1.63 (-1.77,-1.49) | 6798.56 (5530.67,8263.01) | 4682.62 (3848.78,5617.96) | -1.6 (-1.74,-1.46) | 2313.61 (1842.28,2848.59) | 1879.29 (1522.8,2290.8) | -1.02 (-1.17,-0.88) |
| Djibouti | 253.92 (194.47,329.51) | 342.13 (265.99,434.99) | 0.92 (0.82,1.02) | 327.01 (249.3,423.83) | 447.68 (344.04,571.14) | 0.93 (0.84,1.03) | 154.58 (115.6,204.85) | 219.2 (168.08,286.45) | 1.2 (1.08,1.31) |
| Dominica | 208.74 (160.63,267.48) | 278.96 (218.87,350.65) | 0.87 (0.69,1.06) | 212.11 (160.54,274.08) | 283.61 (219.92,358.86) | 0.91 (0.68,1.14) | 202.73 (156.09,260.07) | 265.48 (207.29,337.46) | 0.75 (0.61,0.89) |
| Dominican Republic | 258.76 (204.62,325.49) | 369.52 (291.77,463.83) | 0.9 (0.71,1.09) | 358.34 (279.62,457.81) | 483.88 (380.67,610.56) | 0.72 (0.52,0.91) | 155.59 (122.98,194.02) | 227.26 (177.11,288.75) | 0.94 (0.8,1.07) |
| Ecuador | 361 (289.03,446.59) | 531.39 (424.27,662.03) | 1.04 (0.88,1.2) | 389.21 (307.45,487.83) | 588.8 (467.01,741.59) | 1.19 (1.04,1.33) | 325.25 (259.36,403.01) | 444.99 (353.15,553.82) | 0.73 (0.56,0.91) |
| Egypt | 142.66 (109.7,184.79) | 205.68 (158.21,265.1) | 0.92 (0.78,1.06) | 227.21 (171.03,298.17) | 374.91 (284.3,489.09) | 1.34 (1.22,1.46) | 74.06 (57.15,94.59) | 109.71 (85.02,139.8) | 1.09 (0.96,1.23) |
| El Salvador | 545.09 (433.91,678.94) | 760.41 (599.41,953.98) | 1.13 (0.99,1.26) | 678.42 (533.73,849.89) | 945.95 (744.02,1195.36) | 1.14 (1.02,1.26) | 378.43 (299.49,475.09) | 489.45 (382.62,618.54) | 0.88 (0.7,1.05) |
| Equatorial Guinea | 200.39 (151.2,263.43) | 431.58 (333.56,550.1) | 2.76 (2.61,2.92) | 244.17 (182.94,321.32) | 527.46 (407.39,676.29) | 2.78 (2.64,2.92) | 134.9 (100.24,180.59) | 282.97 (217.03,363.97) | 2.82 (2.64,3) |
| Eritrea | 174.69 (129.59,232.06) | 321.96 (247,414.73) | 1.5 (1.3,1.7) | 209.92 (155.46,279.95) | 390.72 (298.69,503.9) | 1.52 (1.33,1.71) | 97.58 (70.87,130.15) | 176.08 (133.21,228.69) | 1.53 (1.34,1.72) |
| Estonia | 1032.51 (814.2,1298.14) | 831.9 (655.69,1041.03) | -0.66 (-0.73,-0.59) | 1140.32 (897.5,1436.19) | 793.8 (626.06,1003.15) | -1.26 (-1.33,-1.18) | 695.07 (540.11,888.01) | 823.45 (638.78,1039.86) | 0.88 (0.67,1.09) |
| Eswatini | 177.7 (136.37,229.6) | 184.91 (139.6,236.78) | -0.13 (-0.42,0.16) | 202.21 (153.56,262.69) | 201.41 (151.25,259.72) | -0.23 (-0.49,0.03) | 121.45 (91.74,158.58) | 134.18 (100.58,174.39) | 0.02 (-0.3,0.35) |
| Ethiopia | 264.17 (191.82,362.52) | 399.71 (303.08,522.71) | 1.46 (1.33,1.59) | 361.44 (262.06,500.52) | 567.57 (427.23,747.65) | 1.54 (1.45,1.64) | 155.13 (110.84,213.74) | 236.37 (179.22,308.49) | 1.52 (1.43,1.62) |
| Fiji | 207.75 (160.36,266.3) | 271.11 (211.31,343.07) | 0.95 (0.85,1.06) | 259.54 (199.74,334.53) | 324.18 (250.89,410.17) | 0.84 (0.74,0.95) | 123.6 (93.5,159.48) | 155.17 (118,201.72) | 0.79 (0.67,0.91) |
| Finland | 4691.15 (3796.93,5707.38) | 5542.51 (4561.04,6644.76) | 0.45 (0.08,0.83) | 5487.43 (4426.69,6688.4) | 6446.94 (5283.18,7731.89) | 0.44 (0.08,0.81) | 2814.41 (2236.73,3471.98) | 4153.63 (3367.32,5024.16) | 1.19 (0.78,1.6) |
| France | 5022.58 (4095.55,6063.76) | 5378.76 (4445.89,6429.61) | 0.32 (0.23,0.42) | 6313.36 (5129.49,7648.94) | 6837.6 (5622.1,8219.46) | 0.33 (0.24,0.42) | 2440.33 (1960.4,2999.25) | 3208.56 (2622.41,3868.12) | 1.12 (0.97,1.27) |
| Gabon | 368.31 (280.31,479.23) | 465.57 (360.75,595.2) | 0.71 (0.57,0.86) | 449.42 (340.61,586.14) | 542.3 (417.2,690.32) | 0.51 (0.36,0.67) | 239.11 (180.74,314.04) | 321.41 (246.94,414.55) | 0.93 (0.84,1.02) |
| Gambia | 344.13 (265.75,441.53) | 490.16 (378.13,631.47) | 1.07 (1.04,1.1) | 449.29 (344.82,580.63) | 639.36 (490.43,828.23) | 1.03 (1,1.06) | 198.34 (151.33,257.68) | 281.71 (216.34,364.37) | 1.1 (1.08,1.11) |
| Georgia | 325.14 (253.25,415.12) | 605.49 (476.59,761.65) | 2.73 (2.35,3.1) | 347.89 (268.19,447.42) | 682.28 (535.46,861.64) | 2.89 (2.55,3.22) | 270.19 (211.28,344.43) | 449.58 (348.64,573.95) | 2.36 (1.93,2.8) |
| Germany | 3333.89 (2678.51,4095.73) | 4053.55 (3305.41,4869.55) | 0.74 (0.63,0.84) | 4124.11 (3296.82,5094.37) | 5235.75 (4249.24,6318.55) | 0.91 (0.79,1.04) | 1563.21 (1250.46,1932.11) | 2374.33 (1923.3,2893.31) | 1.46 (1.4,1.53) |
| Ghana | 354.7 (270.18,455.88) | 480.05 (373.14,614.3) | 0.75 (0.69,0.81) | 482.83 (365.73,624.73) | 596.41 (460.76,761.54) | 0.4 (0.32,0.47) | 201.39 (151.79,262.84) | 288.82 (221.94,374.73) | 1.05 (1,1.1) |
| Greece | 2326.68 (1854.51,2882.52) | 1650.59 (1337.72,2009.89) | -0.85 (-1.02,-0.69) | 3124.3 (2477.04,3882.78) | 2062.62 (1651.09,2525.96) | -1.07 (-1.28,-0.86) | 1292.03 (1033.59,1605.91) | 1124.4 (903.61,1375.53) | -0.28 (-0.38,-0.18) |
| Greenland | 4152.08 (3258.87,5230.72) | 4822.04 (3886.3,5932.49) | 0.68 (0.59,0.78) | 5342.29 (4158,6758.68) | 6361.27 (5112.63,7865.52) | 0.82 (0.71,0.94) | 2284.01 (1773.25,2892.13) | 3057.74 (2421.3,3825.28) | 1.08 (1,1.16) |
| Grenada | 254.46 (198.45,325.31) | 455.67 (359.17,572.86) | 1.77 (1.57,1.96) | 259.19 (200.25,335.53) | 462.64 (361.82,586.38) | 1.77 (1.58,1.96) | 241.08 (187.3,306.54) | 367 (284.53,467.51) | 1 (0.75,1.26) |
| Guam | 350.34 (274.09,446.16) | 391.19 (307.01,491.94) | 0.46 (0.35,0.57) | 430.78 (333.86,547.84) | 473.56 (370.02,595.39) | 0.37 (0.2,0.53) | 257.22 (197.46,335.03) | 263.99 (204.14,338.3) | 0.31 (0.16,0.46) |
| Guatemala | 479.17 (375.12,604.6) | 676.76 (537.57,848.03) | 0.82 (0.66,0.99) | 562.12 (436.9,713.45) | 774.67 (608.56,979.3) | 0.74 (0.59,0.89) | 381.18 (295.42,486.54) | 554.23 (438.18,694.24) | 0.97 (0.77,1.17) |
| Guinea | 255.12 (194.73,330.61) | 325.87 (252.47,415.13) | 0.6 (0.53,0.67) | 347.56 (261.64,455.43) | 430.07 (329.33,549.59) | 0.42 (0.34,0.51) | 166.06 (126.78,214.07) | 216.64 (166.58,279.7) | 0.75 (0.68,0.83) |
| Guinea-Bissau | 259.91 (195.6,339.8) | 354.25 (269.83,454.9) | 0.92 (0.87,0.96) | 330.92 (248.32,432.89) | 434.51 (328.14,559.94) | 0.75 (0.7,0.8) | 175.17 (130.02,232.63) | 228.97 (173.13,299.88) | 0.82 (0.77,0.87) |
| Guyana | 423.38 (325.28,542.24) | 568.12 (445.59,711.96) | 0.63 (0.5,0.77) | 522.79 (398.64,670.76) | 697.83 (541.97,880.49) | 0.66 (0.52,0.81) | 302.33 (231.65,391.08) | 388.82 (302.44,492.47) | 0.44 (0.31,0.57) |
| Haiti | 216.89 (165.38,281.83) | 241.49 (187.37,309.29) | -0.03 (-0.51,0.44) | 276.34 (208.89,362.29) | 311.61 (238.42,400.26) | 0.04 (-0.39,0.48) | 128.8 (98.22,166.78) | 168.41 (130.14,216.04) | 0.51 (-0.02,1.03) |
| Honduras | 279.24 (217.26,354.13) | 330.95 (258.12,419.67) | 0.42 (0.32,0.51) | 349.4 (267.69,449.07) | 400.57 (310.75,513.4) | 0.45 (0.34,0.55) | 190.12 (149.92,239.38) | 248.09 (191.87,314.64) | 0.57 (0.43,0.71) |
| Hungary | 3572.39 (2799.58,4488.31) | 2180.08 (1702.97,2764.1) | -2.15 (-2.35,-1.96) | 4489.72 (3507.06,5643.92) | 2481.87 (1942.66,3150.87) | -2.49 (-2.69,-2.29) | 1708.07 (1324.7,2182.91) | 1490.69 (1143.38,1921.9) | -0.94 (-1.14,-0.74) |
| Iceland | 3122.69 (2516.42,3834.86) | 3302.11 (2710.8,3973.19) | 0.25 (0.2,0.3) | 4143.65 (3311.29,5108.22) | 4090.98 (3340.26,4941.48) | -0.05 (-0.09,-0.01) | 1720.87 (1380.03,2115.19) | 2281.47 (1853.96,2768.1) | 1.14 (1.03,1.25) |
| India | 1196.57 (876.57,1631.45) | 1681.63 (1270.19,2217.63) | 1.09 (1.06,1.12) | 1815.86 (1325.37,2483.36) | 2427.44 (1827.87,3211.99) | 0.9 (0.87,0.93) | 556 (408.88,753.74) | 758.07 (573.23,998.83) | 0.93 (0.88,0.99) |
| Indonesia | 628.51 (469.73,842.9) | 679.19 (516.43,889.66) | -0.12 (-0.24,0.01) | 957.17 (712.35,1290.1) | 959.78 (726.01,1260.05) | -0.41 (-0.55,-0.27) | 226.96 (170.03,301.73) | 300.59 (226.54,397.15) | 0.67 (0.6,0.75) |
| Iran (Islamic Republic of) | 388.31 (294.37,513.2) | 472.73 (367.87,609.25) | 0.54 (0.5,0.58) | 483.44 (363.67,647.36) | 609.31 (470.75,786.93) | 0.76 (0.71,0.81) | 271.08 (207.57,355.05) | 348.4 (271.1,446.5) | 0.6 (0.51,0.69) |
| Iraq | 223.55 (174.79,284.23) | 248.94 (193.88,317.59) | 0.25 (0.17,0.33) | 289.34 (224.18,373.22) | 301.31 (232.92,386.17) | -0.01 (-0.1,0.09) | 146.2 (113.89,185.36) | 180.21 (139.91,229.75) | 0.66 (0.59,0.72) |
| Ireland | 2633.23 (2120.47,3237.66) | 3048.17 (2496.61,3657.82) | 0.47 (0.26,0.69) | 3594.85 (2882.33,4429.62) | 4027.32 (3285.66,4837.2) | 0.35 (0.18,0.51) | 1108.95 (881.34,1383.44) | 1782.92 (1442.75,2176.66) | 1.66 (1.31,2.02) |
| Israel | 1754.87 (1406.6,2141.27) | 1998.01 (1636.24,2398.31) | 0.47 (0.3,0.65) | 2545.87 (2033.31,3114.46) | 2596.29 (2118.37,3130.04) | 0.08 (-0.08,0.25) | 805.95 (644.53,1003.02) | 1197.47 (970.09,1454.35) | 1.49 (1.29,1.68) |
| Italy | 4913.68 (3810.58,6275.78) | 3676.84 (2936.99,4556.49) | -1.06 (-1.14,-0.98) | 6213.93 (4818.79,7945.26) | 4507.95 (3600.23,5596.54) | -1.18 (-1.24,-1.12) | 2522.53 (1926.7,3284.48) | 2433.43 (1930.09,3040.58) | -0.16 (-0.28,-0.03) |
| Jamaica | 211.7 (167.67,265.39) | 340.56 (269.47,428.15) | 1.52 (1.3,1.74) | 273.64 (212.8,348.05) | 445.39 (346.97,566.5) | 1.48 (1.24,1.72) | 127.84 (100.99,159.19) | 197.41 (155.73,249.06) | 1.53 (1.33,1.73) |
| Japan | 1898.06 (1462.87,2445.86) | 1829.37 (1443.4,2289.58) | -0.25 (-0.44,-0.06) | 2071.51 (1603.14,2653.77) | 1895.18 (1501.96,2362.79) | -0.46 (-0.63,-0.28) | 1517.85 (1153.56,1995.12) | 1692.64 (1321.88,2145.41) | 0.31 (0.08,0.55) |
| Jordan | 255.11 (199.23,325.9) | 341.43 (269.41,431.05) | 1.18 (1.04,1.33) | 301.71 (231.51,390.81) | 430.97 (336.17,551.62) | 1.37 (1.18,1.57) | 209.21 (163.66,264.52) | 263.92 (207.75,330.62) | 1.05 (0.91,1.2) |
| Kazakhstan | 259.66 (205.05,327.01) | 387.29 (305.59,494.04) | 2.37 (1.89,2.86) | 286.96 (223.99,364.25) | 422.87 (329.8,543.05) | 2.28 (1.81,2.76) | 191.55 (151.13,239.97) | 299.43 (236.28,380.84) | 2.66 (2.17,3.16) |
| Kenya | 349.21 (261.05,468.75) | 467.33 (353.53,614.66) | 0.88 (0.84,0.92) | 468.62 (350.12,629.09) | 578.55 (438.3,762.33) | 0.58 (0.48,0.68) | 223.08 (165.37,300.28) | 276.64 (208.49,365.02) | 0.63 (0.6,0.66) |
| Kiribati | 128.28 (97.76,166.17) | 163.4 (125.44,209.41) | 0.74 (0.61,0.88) | 176.06 (133.44,229.27) | 222.99 (169.73,287.59) | 0.72 (0.58,0.86) | 44.92 (33.37,59.06) | 52.13 (39.2,67.16) | 0.37 (0.3,0.43) |
| Kuwait | 287.24 (228.06,357.36) | 383.08 (304.83,476.38) | 0.78 (0.59,0.97) | 334.54 (259.92,424.98) | 452.43 (352.33,569.92) | 0.74 (0.42,1.07) | 215.79 (172.14,269.67) | 332.71 (265.54,413.44) | 1.35 (1.22,1.49) |
| Kyrgyzstan | 245.31 (193.69,307.39) | 229.19 (181.58,286.19) | -0.17 (-0.31,-0.03) | 255.38 (199.02,321.67) | 247.54 (194.37,311.64) | 0 (-0.15,0.14) | 213.7 (170.09,269.63) | 186.93 (146.97,233.39) | -0.48 (-0.63,-0.33) |
| Lao People's Democratic Republic | 199.4 (151.67,261.3) | 384.39 (300.92,486.21) | 2.36 (2.27,2.46) | 246.96 (186.56,326.6) | 496.86 (386.31,630.17) | 2.5 (2.42,2.59) | 124.83 (92.94,165.39) | 231.33 (179.02,296.83) | 2.19 (2.1,2.28) |
| Latvia | 1293.97 (1023.64,1631.7) | 901.86 (710.27,1129.1) | -1.73 (-2,-1.46) | 1480.68 (1165.17,1872.49) | 921.66 (725.59,1154.59) | -2.24 (-2.53,-1.94) | 803.82 (632.49,1014.38) | 768.46 (592.5,983.04) | -0.22 (-0.54,0.11) |
| Lebanon | 289.02 (224.34,367.14) | 446.55 (353.54,559.05) | 1.2 (1.05,1.35) | 394.23 (303.22,504.9) | 593.9 (468.88,745.34) | 1.18 (1.01,1.35) | 148.98 (115.43,191.14) | 268.57 (209.1,339.72) | 1.74 (1.59,1.88) |
| Lesotho | 185.81 (142.24,238.94) | 186.09 (139.38,243.44) | -0.2 (-0.44,0.04) | 204.47 (155.76,263.53) | 202.8 (151.08,268.31) | -0.22 (-0.48,0.05) | 126.48 (94.71,166.41) | 126.68 (93.92,166.83) | -0.23 (-0.43,-0.02) |
| Liberia | 266.36 (204.06,345.81) | 407.3 (316.27,520.64) | 1.35 (1.25,1.45) | 368.04 (278.44,483.23) | 551.86 (424.48,707.08) | 1.24 (1.14,1.34) | 180.48 (137.69,234.6) | 263.66 (204.49,337.76) | 1.22 (1.12,1.33) |
| Libya | 317.3 (249.08,401.1) | 405.5 (318.57,508.35) | 0.88 (0.66,1.09) | 408.61 (317.37,522.55) | 527.73 (408.82,664.19) | 0.94 (0.75,1.13) | 224.61 (176.24,284.16) | 277.47 (218.57,348.86) | 0.78 (0.54,1.02) |
| Lithuania | 981.28 (780.16,1226.1) | 1260.67 (1000.01,1559.95) | 0.77 (0.58,0.95) | 1046.56 (823.3,1310.52) | 1332.54 (1052.73,1641.84) | 0.69 (0.56,0.82) | 837.4 (662.68,1046.42) | 1023.9 (800.59,1301.86) | 0.71 (0.36,1.06) |
| Luxembourg | 3706.74 (2974.04,4556.87) | 4536.89 (3756.85,5427.66) | 0.85 (0.67,1.03) | 4617.18 (3689.65,5688.9) | 5779.83 (4756.4,6959.06) | 0.94 (0.79,1.09) | 1944.2 (1547.27,2433.17) | 2701.29 (2190.64,3275.92) | 1.24 (1.01,1.47) |
| Madagascar | 193.7 (149.53,249.17) | 238.44 (182.73,307.22) | 0.54 (0.5,0.59) | 267.22 (203.45,345.54) | 311.99 (236.8,404.25) | 0.39 (0.34,0.45) | 121.28 (92.79,157.02) | 147.32 (112.9,191.27) | 0.47 (0.4,0.54) |
| Malawi | 257.92 (197.63,332.33) | 396.95 (308.04,511.2) | 1.59 (1.4,1.78) | 340.62 (259.03,441.26) | 485.57 (376.34,626.51) | 1.33 (1.09,1.57) | 157.01 (118.5,204.55) | 231.96 (176.62,302.5) | 1.5 (1.36,1.63) |
| Malaysia | 428.91 (331.51,554.32) | 585.42 (453.89,747.55) | 1.19 (1.12,1.26) | 557.55 (429.1,726.24) | 756.04 (582.7,974.03) | 1.14 (1.09,1.19) | 277.72 (213.7,357.3) | 416.75 (321.11,535.89) | 1.56 (1.49,1.64) |
| Maldives | 313.44 (239.85,404.87) | 671 (522.99,846.31) | 2.64 (2.5,2.78) | 443.14 (332.6,579.14) | 908.65 (702.6,1144.48) | 2.46 (2.31,2.6) | 254.47 (195.82,325.45) | 450.76 (350.16,578.45) | 2.03 (1.89,2.18) |
| Mali | 354.47 (270,459.26) | 485.07 (373.51,618.52) | 0.93 (0.89,0.97) | 511.15 (387.16,664.35) | 715.69 (544.39,918.67) | 0.98 (0.93,1.03) | 192 (145.16,253.05) | 270.55 (207.72,346.91) | 1.08 (1.04,1.13) |
| Malta | 3168.7 (2542.76,3901.48) | 3216.19 (2643.47,3888.23) | 0.04 (-0.05,0.14) | 4347.09 (3472.48,5354.75) | 4204.79 (3437.53,5123.18) | -0.13 (-0.21,-0.04) | 1421.3 (1122.72,1774.31) | 1853.68 (1501.05,2259.27) | 0.92 (0.82,1.01) |
| Marshall Islands | 259.77 (197.57,336.62) | 342.65 (264.01,439.37) | 0.84 (0.79,0.89) | 325.33 (246.42,423.4) | 481.85 (368.62,619.68) | 1.28 (1.19,1.37) | 143.23 (106.97,188.25) | 211.46 (160.44,275.66) | 1.14 (1.03,1.25) |
| Mauritania | 314.41 (240.21,405.95) | 428.91 (333.83,547.74) | 0.82 (0.76,0.87) | 399.94 (300.79,517.85) | 580.81 (448.01,742.87) | 1.01 (0.95,1.07) | 199.84 (152.55,259) | 284.04 (220.27,366.39) | 1.04 (1.01,1.08) |
| Mauritius | 182.91 (142.8,232.71) | 299.52 (235.9,376.69) | 2.32 (2.09,2.55) | 189.92 (146.5,245.96) | 313.98 (244.64,400.47) | 2.33 (2.11,2.55) | 156.72 (121.16,199.55) | 259.32 (203.65,326.84) | 2.36 (2.1,2.63) |
| Mexico | 1085.14 (823.4,1434.74) | 865.28 (669.13,1117.74) | -0.6 (-0.75,-0.44) | 1412.43 (1064.27,1875.22) | 1077.84 (832.22,1392.78) | -0.7 (-0.87,-0.52) | 763.99 (585.71,1003.42) | 602.41 (464.85,781.42) | -0.7 (-0.82,-0.58) |
| Micronesia (Federated States of) | 259.59 (196.12,335.46) | 423.47 (327.84,535.32) | 1.72 (1.65,1.8) | 332.78 (251.39,429.73) | 532 (410.99,672.72) | 1.64 (1.58,1.7) | 142.11 (105.62,186.24) | 231.36 (175.01,298.18) | 1.66 (1.61,1.72) |
| Monaco | 2153.12 (1706.9,2680.23) | 2299.56 (1856.4,2806.92) | 0.22 (0.13,0.32) | 2779.87 (2187.83,3472.67) | 3047.43 (2436.33,3751.95) | 0.32 (0.22,0.42) | 1087.14 (851.9,1363.23) | 1299.35 (1050.88,1591.48) | 0.63 (0.5,0.75) |
| Mongolia | 217.63 (167.51,281.3) | 328.63 (256.38,418.68) | 1.72 (1.51,1.93) | 255.93 (196.68,330.81) | 373.02 (291.73,473.31) | 1.58 (1.37,1.8) | 160.43 (121.01,211.69) | 253.7 (194.72,325.63) | 1.95 (1.75,2.16) |
| Montenegro | 1203.4 (943.44,1535.35) | 1038.86 (809.42,1334.99) | -0.39 (-0.52,-0.26) | 1286.47 (1004.87,1641.08) | 1066.9 (828.06,1379.56) | -0.5 (-0.66,-0.34) | 1046.15 (814.84,1336.7) | 956.68 (736.95,1227.69) | -0.14 (-0.27,0) |
| Morocco | 377.08 (289.31,485.54) | 613.8 (473.11,785.32) | 1.47 (1.44,1.5) | 598.27 (453.54,782.11) | 949.38 (726.88,1222.03) | 1.38 (1.34,1.41) | 153.08 (120.06,194.3) | 236.29 (183.5,301.03) | 1.37 (1.34,1.4) |
| Mozambique | 256.26 (195.23,332.93) | 349.08 (265.22,450.04) | 0.81 (0.75,0.88) | 331.94 (251.34,434.04) | 413.59 (311.4,534.71) | 0.47 (0.39,0.56) | 158.26 (118.88,208.43) | 219 (164.96,287.06) | 0.94 (0.9,0.99) |
| Myanmar | 357.39 (273.55,462.83) | 767.21 (605.93,960.45) | 2.72 (2.59,2.85) | 431.29 (328.86,560.86) | 898.45 (706.29,1126.61) | 2.63 (2.49,2.77) | 245.39 (185.65,318.03) | 522.64 (408.79,659.53) | 2.68 (2.57,2.78) |
| Namibia | 171.04 (130.75,221.39) | 242.77 (186.95,311.26) | 1.18 (0.94,1.42) | 199.44 (151.03,259.46) | 271.38 (207.52,348.68) | 1.06 (0.81,1.3) | 118.19 (89.21,155.38) | 175.27 (133.42,229.05) | 1.29 (1.09,1.49) |
| Nauru | 260.59 (199.04,336.32) | 466.83 (358.17,597.88) | 1.89 (1.86,1.92) | 412.07 (314.51,530.76) | 602.38 (462.26,770.09) | 1.19 (1.15,1.23) | 181.26 (135.06,238.62) | 280.28 (210.85,366.44) | 1.39 (1.22,1.56) |
| Nepal | 584.34 (437.98,773.3) | 968.68 (748.69,1254.8) | 1.49 (1.39,1.59) | 900.67 (667.84,1200.44) | 1407.62 (1082.61,1830.44) | 1.3 (1.19,1.41) | 268.94 (204.18,353.69) | 471.34 (361.59,611.24) | 1.77 (1.67,1.88) |
| Netherlands | 3121.06 (2521.94,3806.18) | 5280.9 (4352.84,6324.31) | 2.58 (1.75,3.42) | 4049.25 (3251.6,4956.26) | 7105.35 (5810.11,8528.21) | 2.72 (1.84,3.6) | 1366.35 (1085.81,1685.86) | 2747.38 (2215.38,3331.56) | 3.25 (2.46,4.05) |
| New Zealand | 4143.96 (3207.77,5278.83) | 4125.7 (3311.26,5089.58) | 0.54 (0.36,0.73) | 5254.68 (4063.73,6699.31) | 5179.51 (4164.29,6405.9) | 0.54 (0.35,0.74) | 2142.38 (1625.59,2791.57) | 2751.69 (2163.99,3446.41) | 1.3 (1.05,1.56) |
| Nicaragua | 588.26 (466.43,730.64) | 938.8 (747.67,1163.68) | 1.4 (1.21,1.58) | 709.15 (557.92,888.26) | 1222.63 (969.56,1520.78) | 1.68 (1.46,1.9) | 380.8 (300.33,475.62) | 503.16 (397.63,629.57) | 1 (0.93,1.08) |
| Niger | 342.5 (258.96,449.69) | 471.84 (364.15,605.46) | 1.13 (1.05,1.22) | 476.17 (357.73,630.37) | 669.62 (516.21,859.77) | 1.14 (1.05,1.23) | 182.71 (137.06,240.28) | 261.06 (199.35,338.99) | 1.24 (1.14,1.33) |
| Nigeria | 368.49 (273.45,496.54) | 509.85 (384.67,669.38) | 1.13 (1.04,1.23) | 483.01 (356.79,652.82) | 719.78 (542.29,946.18) | 1.46 (1.36,1.57) | 207.89 (153.21,281.81) | 284.53 (215.31,374.91) | 1.15 (1.06,1.25) |
| Niue | 343.1 (265.24,435.83) | 477.7 (372.02,603.55) | 1.03 (0.94,1.12) | 404.86 (311.73,516.82) | 580.08 (451.6,729.1) | 1.18 (1.03,1.32) | 195.8 (149.13,252.29) | 285.53 (217.78,368.44) | 1.24 (1.21,1.27) |
| North Macedonia | 369.13 (284.88,472.76) | 867.72 (669.58,1099.2) | 3.39 (2.9,3.87) | 452.09 (348.63,580.13) | 1095.94 (839.79,1399.18) | 3.43 (2.93,3.93) | 265.6 (203.57,344.59) | 529.75 (408.79,678.98) | 2.84 (2.45,3.23) |
| Northern Mariana Islands | 547.22 (420.19,699.04) | 901.63 (710.69,1115.38) | 1.62 (1.53,1.72) | 635.63 (484.18,814.61) | 1151.63 (908.97,1416.94) | 2.07 (1.83,2.32) | 438.05 (336.16,562.55) | 559.47 (433.1,712.02) | 0.52 (0.43,0.62) |
| Norway | 4860.7 (3772.55,6212.3) | 5438.9 (4389.27,6685.73) | 0.38 (0.26,0.5) | 6057.86 (4710.6,7756.91) | 6893.37 (5577.73,8465.31) | 0.44 (0.35,0.54) | 2745.07 (2094.84,3574.27) | 3432.85 (2728.01,4295.67) | 0.73 (0.57,0.9) |
| Oman | 595.49 (451.94,773.13) | 876.08 (677.88,1120.83) | 1.02 (0.85,1.2) | 705.44 (527.46,928.97) | 1081.03 (832.05,1387.76) | 1.23 (1.05,1.41) | 379.98 (289.76,491.34) | 562.93 (433.3,731.52) | 0.96 (0.7,1.22) |
| Pakistan | 190.14 (141.58,256.31) | 218.22 (164.23,287.48) | 0.48 (0.37,0.6) | 238.39 (177.06,322.86) | 255.05 (191.42,337.58) | 0.29 (0.16,0.41) | 155.6 (114.82,210.4) | 184.6 (138.77,244.89) | 0.56 (0.44,0.67) |
| Palau | 801.1 (616.34,1025.95) | 946.5 (742.22,1203.93) | 0.52 (0.43,0.61) | 1056.45 (810.32,1350.99) | 1374.21 (1066.31,1743.91) | 0.83 (0.78,0.88) | 457.68 (346.58,598.51) | 604.62 (467.44,777.51) | 0.92 (0.86,0.98) |
| Palestine | 340.13 (260.74,432.82) | 481.07 (375.1,611.28) | 1.16 (0.94,1.38) | 423.31 (321.84,543.58) | 580.16 (447.89,742.05) | 1.01 (0.8,1.22) | 225.83 (173.93,290.66) | 320.93 (250.76,410.1) | 1.19 (0.87,1.5) |
| Panama | 569.94 (451.68,710.48) | 420.93 (337.22,516.83) | -1.3 (-1.45,-1.16) | 755.8 (592.59,949.51) | 490.46 (389.28,607.44) | -1.76 (-1.92,-1.6) | 352.86 (279.61,439.82) | 329.81 (263.13,409.34) | -0.4 (-0.52,-0.28) |
| Papua New Guinea | 604.68 (458.05,796.2) | 940.02 (729.7,1187.1) | 1.26 (1.18,1.34) | 1162.75 (878,1539.35) | 1900.1 (1469.88,2407.42) | 1.45 (1.38,1.52) | 52.2 (39.64,67.82) | 70.36 (54.47,89.58) | 0.85 (0.76,0.94) |
| Paraguay | 473.85 (377.26,589.53) | 605.75 (479.45,758.16) | 1.06 (0.96,1.16) | 547.34 (432.19,686.48) | 736.72 (578.32,926.78) | 1.32 (1.2,1.43) | 371.04 (295.9,462.54) | 409.74 (324.22,512.42) | 0.41 (0.34,0.47) |
| Peru | 249.23 (197.36,315.07) | 404.17 (318.35,506.9) | 1.61 (1.5,1.71) | 296.95 (233.02,377.72) | 469.37 (366.22,594.34) | 1.58 (1.46,1.7) | 193.84 (152.27,245.12) | 324.91 (256.8,406.5) | 1.66 (1.57,1.75) |
| Philippines | 372.36 (281.43,496.5) | 419.25 (322.21,546.99) | 0.31 (0.15,0.47) | 512.12 (385.76,682.77) | 534.95 (410.88,699.13) | 0.1 (-0.08,0.29) | 237.48 (179.6,313.2) | 245.02 (186.37,321.76) | -0.03 (-0.15,0.1) |
| Poland | 1556.07 (1173.39,2066.99) | 1498.99 (1162.39,1933.35) | -0.28 (-0.41,-0.14) | 1903.88 (1433.67,2534.34) | 1624.15 (1260.18,2093.59) | -0.72 (-0.85,-0.6) | 790.23 (590.42,1057.36) | 1147.51 (875.43,1490.33) | 1.22 (1.01,1.43) |
| Portugal | 1825.55 (1464.22,2242.67) | 2326.13 (1880.11,2839.11) | 1.09 (0.96,1.22) | 2242.74 (1780.94,2782.11) | 2815.87 (2260.44,3472.82) | 1.05 (0.91,1.2) | 1106.5 (888.89,1356.06) | 1576.46 (1273.56,1924.29) | 1.41 (1.32,1.51) |
| Puerto Rico | 536.86 (425.23,672.96) | 912.44 (727.08,1142.29) | 2.06 (1.92,2.2) | 658.2 (515.81,830.08) | 1013.33 (801.19,1276.82) | 1.71 (1.59,1.83) | 372.75 (294.85,469.25) | 755.6 (598.53,951.36) | 2.66 (2.49,2.84) |
| Qatar | 305.91 (236.45,392.66) | 579.57 (454.75,728.91) | 2.19 (2.08,2.3) | 302.43 (227.75,396.6) | 596.28 (458.58,761.72) | 2.24 (2.09,2.39) | 284.67 (220.2,363.59) | 543.17 (427.37,688.89) | 2.22 (2.12,2.33) |
| Republic of Korea | 1856.34 (1465.75,2331.9) | 3901.2 (3177.78,4744.07) | 2.31 (1.91,2.72) | 2080.15 (1628.01,2615.86) | 4445.13 (3609.75,5426.13) | 2.32 (1.9,2.74) | 1331.25 (1042.09,1692.41) | 2743.42 (2207.06,3375.04) | 2.44 (2.11,2.76) |
| Republic of Moldova | 659.9 (519.08,831.04) | 499.71 (392.95,630.67) | -0.72 (-0.91,-0.52) | 775.16 (607.86,983.25) | 501.37 (392.72,633.44) | -1.32 (-1.55,-1.09) | 459.84 (360.29,579.27) | 481.27 (371.01,616.02) | 0.55 (0.34,0.76) |
| Romania | 599.11 (475.17,743.76) | 728.1 (568.4,924.63) | 0.84 (0.76,0.93) | 621.46 (485.21,778.54) | 720.31 (560.31,926.21) | 0.62 (0.54,0.71) | 561.61 (444.32,697.44) | 725.9 (562,924.49) | 1.13 (1.03,1.23) |
| Russian Federation | 539.53 (411,711.44) | 710.58 (546.61,927.75) | 1.23 (1.06,1.39) | 570.59 (434.81,754.85) | 735.08 (566.29,963.88) | 1.08 (0.95,1.21) | 403.26 (303.73,532.87) | 614.61 (465.09,806.82) | 1.92 (1.63,2.21) |
| Rwanda | 228.76 (172.26,298.22) | 403.55 (314.45,512.44) | 2.32 (2.14,2.51) | 293.31 (219.84,382.03) | 495.6 (384.22,632.74) | 2.09 (1.94,2.25) | 135.92 (100.41,180.52) | 231.82 (178.41,295.47) | 2.28 (2.08,2.47) |
| Saint Kitts and Nevis | 279.27 (215.95,352.7) | 520.37 (407.95,651.13) | 1.87 (1.67,2.07) | 302.01 (231.22,385.46) | 569.52 (443.1,713.77) | 1.9 (1.67,2.13) | 229.52 (178.28,291.97) | 417.81 (325.43,524.85) | 1.89 (1.71,2.06) |
| Saint Lucia | 228.32 (178.02,291.17) | 347.76 (278.4,431.27) | 1.27 (1.06,1.48) | 255.3 (195.39,328.41) | 388.11 (307.91,483.61) | 1.27 (1.03,1.52) | 180.89 (141.17,230.78) | 285.68 (226.93,357.71) | 1.48 (1.35,1.61) |
| Saint Vincent and the Grenadines | 249.52 (195.61,316) | 395.16 (312.91,489.52) | 1.44 (1.39,1.5) | 211.78 (162.95,271.45) | 335.42 (260.57,421.35) | 1.53 (1.44,1.61) | 311.13 (241.79,394.96) | 459.35 (362.3,575.86) | 1.16 (1.1,1.22) |
| Samoa | 332 (258.46,422.16) | 528.25 (415.32,662.31) | 1.62 (1.56,1.69) | 406.44 (315.1,515.93) | 705.7 (553.62,884.97) | 1.91 (1.81,2.02) | 191.85 (145.77,250.74) | 292.13 (227.12,373.52) | 1.36 (1.32,1.41) |
| San Marino | 2306.42 (1850.3,2818.33) | 2685.9 (2169.92,3275.33) | 0.69 (0.55,0.82) | 2865.83 (2286.17,3497.56) | 3438.77 (2756.78,4238.66) | 0.78 (0.63,0.93) | 1483.96 (1180.93,1839.4) | 1808.35 (1457.02,2188.71) | 0.88 (0.75,1.01) |
| Sao Tome and Principe | 434.09 (335.05,556.11) | 731.95 (571.25,933.13) | 1.72 (1.64,1.81) | 414.79 (316.1,536.71) | 675.17 (520.44,866.03) | 1.59 (1.53,1.64) | 424.48 (323.91,549.26) | 781.7 (606.46,1008.67) | 1.99 (1.9,2.08) |
| Saudi Arabia | 964.37 (749.94,1242.7) | 1417.57 (1109.83,1788.86) | 1.15 (1.09,1.21) | 1218.26 (935.18,1590.41) | 1683.37 (1301.89,2154.63) | 0.87 (0.81,0.93) | 719.05 (560.09,922.04) | 1215.7 (947.87,1535.4) | 1.6 (1.51,1.7) |
| Senegal | 318.12 (244.1,409.77) | 470.45 (365.65,600.61) | 1.19 (1.13,1.24) | 443.18 (338.84,574.88) | 635.02 (489.24,815.29) | 1.08 (1.03,1.14) | 186.37 (142.23,241.56) | 271.62 (210.71,350.53) | 1.18 (1.12,1.23) |
| Serbia | 679.16 (524.8,868.14) | 868.36 (674.14,1104.2) | 1 (0.91,1.09) | 809.37 (619.82,1040.83) | 999.02 (776.09,1275.08) | 0.94 (0.83,1.06) | 469.23 (363.31,603.02) | 659.82 (509.14,850.04) | 1.31 (1.21,1.42) |
| Seychelles | 291.12 (227.13,371.06) | 326.18 (255.51,412.74) | 0.49 (0.42,0.56) | 279.75 (216.18,357.52) | 312.17 (242.57,396.92) | 0.46 (0.38,0.55) | 288.32 (221.86,373.79) | 320.07 (248.42,409.34) | 0.49 (0.41,0.57) |
| Sierra Leone | 277.25 (211.16,356.74) | 363.56 (283.02,459.2) | 0.75 (0.66,0.85) | 372.6 (282.56,480.85) | 474.5 (366.77,601.53) | 0.61 (0.51,0.71) | 180.08 (136.05,236.11) | 238.08 (182.94,303.1) | 0.85 (0.74,0.97) |
| Singapore | 1086.82 (869.4,1355.57) | 1642.94 (1336.2,2004.34) | 1.15 (0.72,1.57) | 1205.2 (954.27,1517.24) | 1874.92 (1502.25,2297.14) | 1.14 (0.67,1.6) | 911.32 (723.51,1135.12) | 1349.64 (1097.02,1657.69) | 1.3 (0.98,1.62) |
| Slovakia | 1919.32 (1486.62,2456.99) | 1898.58 (1500.72,2371.64) | 0.33 (0,0.66) | 2332.17 (1798.18,2996.82) | 2089.72 (1652.69,2623.41) | 0.07 (-0.36,0.5) | 1146.72 (878.73,1477.24) | 1426.46 (1106.13,1805.47) | 0.81 (0.71,0.91) |
| Slovenia | 3138.4 (2454.06,3942.57) | 3469.64 (2789.72,4251.36) | 0.9 (0.66,1.14) | 3839.15 (2996.11,4811.71) | 3991.61 (3211.49,4882.82) | 0.71 (0.49,0.92) | 1746.67 (1342.67,2241.38) | 2381.43 (1877.73,2990.25) | 1.51 (1.24,1.77) |
| Solomon Islands | 644.87 (482.6,850.36) | 1063.52 (813.75,1369.47) | 1.64 (1.58,1.71) | 1321.97 (982.3,1761.38) | 1908.15 (1455.44,2455.5) | 1.14 (1.08,1.2) | 127.29 (95.05,167.55) | 218.42 (164.69,284.41) | 1.73 (1.69,1.77) |
| Somalia | 166.9 (125.65,219.39) | 212.01 (161.55,272.86) | 0.8 (0.77,0.83) | 216.23 (161.48,285.85) | 258.98 (196.43,334.55) | 0.59 (0.55,0.63) | 106.13 (78.48,141.14) | 121.91 (91.65,158.74) | 0.49 (0.46,0.51) |
| South Africa | 195.59 (149.79,254.73) | 154.03 (116.68,202.87) | -1.13 (-1.29,-0.96) | 225.01 (171.75,294.3) | 170.58 (129.01,224.95) | -1.29 (-1.49,-1.1) | 142.53 (108.83,185.58) | 118.4 (89.07,156.57) | -0.82 (-0.95,-0.69) |
| South Sudan | 193.66 (148.01,251.6) | 232.51 (178.5,298.49) | 0.52 (0.49,0.56) | 265.82 (203.27,345.43) | 327.32 (249.74,422.64) | 0.59 (0.55,0.64) | 125.17 (93.6,165.02) | 148.26 (112.25,191.47) | 0.55 (0.47,0.63) |
| Spain | 1726.7 (1381.94,2127.82) | 2699.13 (2202.09,3277.44) | 1.65 (1.45,1.86) | 2124.26 (1676.83,2648.03) | 3481.68 (2818.03,4254.81) | 1.85 (1.64,2.06) | 1060.61 (849.44,1295.94) | 1561.92 (1267.07,1902.3) | 1.35 (1.17,1.53) |
| Sri Lanka | 493.02 (383.92,624.61) | 1129.96 (887.87,1419.88) | 2.6 (2.45,2.74) | 646 (499.92,819) | 1364.23 (1067.92,1714.05) | 2.32 (2.19,2.45) | 342.75 (264.7,442.83) | 711.64 (555.33,921.02) | 2.26 (2.13,2.4) |
| Sudan | 191.5 (148.02,243.11) | 279.12 (219.42,352.26) | 1.14 (1.08,1.19) | 242.96 (184.28,314.15) | 368.85 (286.18,468.15) | 1.31 (1.24,1.37) | 139.06 (107.63,175.92) | 205.77 (161.37,260.97) | 1.19 (1.14,1.25) |
| Suriname | 299.71 (234.74,379.42) | 432.11 (341.3,541.6) | 1.13 (1.05,1.21) | 390.72 (304.35,497.97) | 545.86 (428.33,688.03) | 1 (0.91,1.09) | 181.58 (141.09,233.68) | 260.19 (205.33,329.3) | 1.07 (0.98,1.16) |
| Sweden | 3911.06 (3011.85,5085.17) | 4265.52 (3395.22,5287.48) | 0.4 (0.25,0.55) | 4835.53 (3704.19,6308.35) | 5204.55 (4133.91,6451.94) | 0.36 (0.22,0.5) | 2329.95 (1774.96,3081.79) | 3016.46 (2361.84,3784.76) | 0.96 (0.77,1.14) |
| Switzerland | 6525.26 (5378.97,7865.07) | 5741.25 (4784.48,6839.16) | -0.27 (-0.41,-0.13) | 8227.08 (6766.79,9928.66) | 7345.58 (6089.94,8753.29) | -0.23 (-0.39,-0.07) | 3346.83 (2689.15,4088.19) | 3494.25 (2860.44,4225.1) | 0.26 (0.18,0.34) |
| Syrian Arab Republic | 236.93 (184.66,300.11) | 352.2 (276.87,444.01) | 0.83 (0.49,1.18) | 358.58 (276.29,461.86) | 546.17 (426.68,687.26) | 0.83 (0.44,1.22) | 131.08 (102.44,165.91) | 217.1 (167.49,277.31) | 1.16 (0.75,1.56) |
| Taiwan (Province of China) | 640.75 (505.35,811.2) | 592.61 (465.99,743.76) | -0.37 (-0.43,-0.31) | 781.14 (608.6,992.75) | 654.97 (510.72,827.88) | -0.66 (-0.7,-0.61) | 483.13 (380.89,614.64) | 508.1 (398.11,644.4) | -0.03 (-0.14,0.07) |
| Tajikistan | 211.65 (168.75,263.64) | 183.51 (143.55,232.76) | -0.74 (-0.86,-0.63) | 221.51 (173.4,279.65) | 200.39 (154.76,258.79) | -0.52 (-0.61,-0.43) | 192.25 (153.07,238.8) | 164.26 (127.74,208.04) | -0.83 (-0.97,-0.68) |
| Thailand | 467.32 (365.73,595.49) | 639.2 (505.41,807.79) | 0.99 (0.83,1.15) | 593.38 (461.15,762.2) | 769.81 (605.49,981.93) | 0.77 (0.58,0.96) | 268.88 (209.87,346.44) | 450.94 (354.36,568.27) | 1.76 (1.65,1.88) |
| Timor-Leste | 219.21 (169.89,282.93) | 397.27 (308.92,503.76) | 2.3 (2.18,2.43) | 286.29 (220.42,369.01) | 525.16 (406.88,669.27) | 2.31 (2.17,2.45) | 145.15 (110.87,188.86) | 261.93 (201.07,334.03) | 2.27 (2.14,2.4) |
| Togo | 335.08 (256.82,433.56) | 434.87 (339.48,552.32) | 0.69 (0.55,0.84) | 435.78 (330.67,567.81) | 520.56 (404.39,665.62) | 0.37 (0.21,0.52) | 211.34 (161.46,274.46) | 268.59 (207.13,343.02) | 0.66 (0.54,0.79) |
| Tokelau | 264.99 (205.19,335.77) | 450.23 (352.75,563.95) | 1.75 (1.72,1.78) | 364.57 (278.82,465.02) | 640.36 (497.68,802.9) | 1.92 (1.87,1.98) | 142.73 (109.86,182.02) | 230.58 (178.78,293.38) | 1.56 (1.52,1.6) |
| Tonga | 318.88 (251.71,399.16) | 397.56 (311.78,494.3) | 0.61 (0.54,0.69) | 488.99 (383.96,615.04) | 594.64 (465.04,740.04) | 0.52 (0.47,0.57) | 79.51 (61.06,102.21) | 102.79 (79.29,131.9) | 0.66 (0.56,0.75) |
| Trinidad and Tobago | 320.2 (252.08,402.26) | 288.2 (229.11,357.58) | -0.27 (-0.33,-0.21) | 396.09 (308.73,501.73) | 337.2 (265.04,423.25) | -0.48 (-0.53,-0.43) | 192.94 (152.16,243.07) | 211.15 (167.08,261.89) | 0.48 (0.39,0.56) |
| Tunisia | 303.83 (238.18,385.51) | 452.56 (356.38,570.86) | 1.37 (1.29,1.45) | 380.59 (293.84,486.04) | 557.14 (436.41,704.27) | 1.31 (1.23,1.39) | 214.6 (168.98,272.26) | 312.05 (243.55,394.81) | 1.22 (1.13,1.3) |
| Turkey | 293.76 (229.64,375.12) | 873.75 (691.35,1093.79) | 4.24 (3.88,4.61) | 380.43 (295.58,488.9) | 1170.29 (919.8,1476.49) | 4.41 (4.08,4.75) | 157.76 (121.64,202.73) | 450.35 (353.59,570.27) | 4 (3.73,4.28) |
| Turkmenistan | 163.84 (128.47,207.03) | 152.79 (119,194.28) | -0.01 (-0.2,0.18) | 187.94 (143.72,243.49) | 176.49 (134.45,228.24) | 0.06 (-0.11,0.23) | 116.94 (93.36,146.04) | 115.7 (90.31,146.22) | 0.2 (-0.04,0.45) |
| Tuvalu | 250.17 (191.74,319.81) | 419.22 (326.73,528.26) | 1.68 (1.58,1.78) | 303.62 (230.67,388.54) | 541.8 (419.93,683.79) | 1.96 (1.84,2.09) | 138.49 (104.13,179.78) | 235.95 (180.62,303.48) | 1.73 (1.63,1.83) |
| Uganda | 242.67 (185.22,314.25) | 397.41 (308.36,505.41) | 1.86 (1.72,2.01) | 315.96 (239.95,409.66) | 490.95 (378.29,626.52) | 1.7 (1.55,1.86) | 148.96 (110.21,196.37) | 225.48 (172.94,290.36) | 1.6 (1.46,1.75) |
| Ukraine | 673.93 (509.81,893.67) | 547.75 (421.08,715.42) | -0.85 (-1.03,-0.66) | 695.69 (523.73,923.47) | 510.58 (389.95,672.78) | -1.21 (-1.39,-1.04) | 584.29 (441.72,776.45) | 579.38 (442.11,760.09) | -0.13 (-0.37,0.11) |
| United Arab Emirates | 307.54 (241.12,388.43) | 282.58 (226.57,349.98) | -0.77 (-1.14,-0.39) | 383.42 (294.03,490.35) | 383.07 (297.07,482.24) | -0.35 (-0.81,0.11) | 228.52 (180.81,288.74) | 274.77 (220.06,341) | 0.18 (0.07,0.3) |
| United Kingdom | 2358.51 (1835.59,3013.18) | 3260.89 (2602.73,4047.96) | 1.34 (1.15,1.53) | 3038.4 (2358.03,3893.98) | 4321.48 (3445.86,5372.44) | 1.43 (1.25,1.61) | 1093.41 (843.44,1414.76) | 1894.71 (1507.4,2366.36) | 2.11 (1.86,2.35) |
| United Republic of Tanzania | 281.66 (216.85,363.42) | 405.09 (314.13,518.92) | 1.33 (1.19,1.47) | 378.38 (289.71,487.98) | 539.28 (414.74,696.06) | 1.26 (1.1,1.43) | 163.11 (123.1,215.24) | 230.23 (177.66,295.09) | 1.31 (1.2,1.42) |
| United States of America | 2431.1 (1863.83,3157.14) | 3998.89 (3110.98,5088.49) | 1.77 (1.62,1.93) | 2977.33 (2281.57,3872.95) | 5090.08 (3961.29,6486.28) | 1.9 (1.75,2.05) | 1496.95 (1135.63,1972.58) | 2593.1 (1998.17,3313.73) | 2.02 (1.86,2.18) |
| United States Virgin Islands | 400.36 (312.97,512.88) | 537.64 (424.4,672.9) | 0.96 (0.88,1.05) | 446.29 (344.08,576.79) | 579.35 (455.25,727.41) | 0.82 (0.68,0.95) | 310.56 (241.58,395.25) | 442.17 (343.57,560.11) | 1.13 (1.08,1.18) |
| Uruguay | 1536.8 (1230.27,1896.9) | 1903.43 (1540.43,2323.79) | 0.75 (0.68,0.82) | 1976.53 (1577.63,2448.61) | 2441.36 (1962.25,2974.88) | 0.75 (0.67,0.82) | 804.16 (634.12,1001.86) | 948.08 (759.51,1175.79) | 0.55 (0.49,0.6) |
| Uzbekistan | 192.11 (151.56,241.37) | 175.88 (136.39,225.09) | -0.06 (-0.2,0.07) | 211.93 (164.5,269.47) | 192.15 (146.37,250.26) | -0.08 (-0.21,0.06) | 154.44 (121.56,192.62) | 148.99 (115.19,189.32) | 0.13 (-0.02,0.27) |
| Vanuatu | 188.81 (144.54,241.89) | 279.7 (216.34,353.59) | 1.27 (1.21,1.33) | 271.8 (207.01,349.11) | 373.67 (287.39,472.25) | 1.01 (0.94,1.09) | 114.55 (85.84,150.61) | 167.54 (126.74,216.98) | 1.24 (1.22,1.26) |
| Venezuela (Bolivarian Republic of) | 644.35 (510.3,805.87) | 574.36 (451.3,717.69) | -0.5 (-0.59,-0.4) | 878.37 (692.29,1100.64) | 761.89 (595.78,956.48) | -0.6 (-0.68,-0.51) | 346.61 (273.53,438.37) | 292.96 (228.16,371.1) | -0.57 (-0.71,-0.43) |
| Viet Nam | 712.14 (543.13,933.1) | 1381.82 (1076.84,1760.54) | 2.25 (2.06,2.44) | 905.67 (688.31,1192.08) | 1729.26 (1343.62,2200.77) | 2.19 (1.97,2.41) | 302.08 (227.07,401.57) | 608.35 (462.66,802.39) | 2.36 (2.24,2.48) |
| Yemen | 197.87 (151.93,255.37) | 254.79 (198.29,321.93) | 0.87 (0.72,1.01) | 228.66 (172.67,299.67) | 316.63 (244.35,404.27) | 1.12 (0.95,1.28) | 134.35 (102.82,173.26) | 185.5 (144.75,235.39) | 1.16 (1,1.32) |
| Zambia | 273.59 (209.07,353.76) | 408.13 (314.39,523.12) | 1.56 (1.33,1.79) | 383.29 (291.5,496.96) | 522.35 (399.51,670.26) | 1.18 (0.97,1.39) | 176.41 (132.64,231.4) | 251.06 (192.63,326.82) | 1.46 (1.24,1.68) |
| Zimbabwe | 320.42 (246.45,412.59) | 281.54 (212.49,362.36) | -0.63 (-0.94,-0.32) | 393.75 (299.92,509.59) | 356.84 (266.29,461.69) | -0.45 (-0.79,-0.11) | 183.58 (139.66,239.28) | 162.18 (122.09,213.5) | -0.56 (-0.77,-0.35) |

| **Table S5. The ASYR for 1990 and 2021, and the EAPC of ASYR from 1990 to 2021.** | | | | | | | | | |
| --- | --- | --- | --- | --- | --- | --- | --- | --- | --- |
|  | **Both** | | | **Female** | | | **Male** | | |
|  | **ASYR per 100,000 population, 1990** | **ASYR per 100,000 population, 2021** | **EAPC** | **ASYR per 100,000 population, 1990** | **ASYR per 100,000 population, 2021** | **EAPC** | **ASYR per 100,000 population, 1990** | **ASYR per 100,000 population, 2021** | **EAPC** |
| Global | 177.19 (118.04,251.3) | 164.5 (109.93,233.22) | -0.18 (-0.22,-0.14) | 225.96 (150.65,320.58) | 207.9 (139.02,294.47) | -0.19 (-0.23,-0.16) | 98.06 (64.81,139.81) | 103.08 (68.38,147.34) | 0.2 (0.14,0.26) |
| High SDI | 231.56 (154.31,327.92) | 236.3 (154.2,339.97) | 0.33 (0.25,0.4) | 284.73 (189.64,402.71) | 290.34 (189.1,417.21) | 0.33 (0.25,0.42) | 130.98 (86.1,187.12) | 160.8 (104.46,232.36) | 0.93 (0.83,1.03) |
| High-middle SDI | 151.15 (100.87,214.93) | 116.07 (75.91,167.87) | -1.04 (-1.15,-0.93) | 180.81 (120.75,256.84) | 137.94 (90.41,199.08) | -1.05 (-1.16,-0.95) | 92.99 (61.67,132.76) | 79.04 (51.19,115.3) | -0.71 (-0.83,-0.59) |
| Middle SDI | 128.18 (83.35,184.67) | 123.85 (81.74,176.74) | -0.29 (-0.41,-0.17) | 167.69 (109.08,241.2) | 160.91 (106.44,229.19) | -0.29 (-0.4,-0.18) | 74.87 (48.56,109.13) | 72.87 (47.62,105.15) | -0.32 (-0.45,-0.19) |
| Low-middle SDI | 161.39 (102.95,236.53) | 174.59 (113.49,248.28) | 0.24 (0.1,0.37) | 236.81 (150.89,349.18) | 246.49 (160.13,351.29) | 0.12 (-0.01,0.26) | 83.32 (53.24,122.57) | 86.81 (56.47,124.3) | 0.03 (-0.11,0.18) |
| Low SDI | 115.4 (73.5,168.51) | 140.55 (91.62,200.8) | 0.64 (0.55,0.73) | 164.31 (104.76,239.55) | 198.01 (128.99,283.13) | 0.61 (0.52,0.7) | 63.28 (40.24,93.37) | 75.11 (49.13,108.53) | 0.54 (0.42,0.65) |
| Andean Latin America | 62.83 (41.63,88.82) | 61.46 (41.15,85.91) | -0.18 (-0.28,-0.07) | 71.31 (46.66,102.16) | 69.72 (46.5,98.18) | -0.12 (-0.24,0) | 52.58 (34.47,75.16) | 50.82 (34.07,71.53) | -0.28 (-0.37,-0.18) |
| Australasia | 230.58 (151.67,331.43) | 338.62 (225.18,484.8) | 1.68 (1.51,1.84) | 286.57 (187.96,412.6) | 411.66 (272.09,587.86) | 1.61 (1.46,1.76) | 131.53 (84.68,190.06) | 244.08 (161.23,348.53) | 2.42 (2.17,2.66) |
| Caribbean | 121.3 (80.45,170.22) | 134.4 (88.86,188.7) | 0.27 (0.18,0.37) | 171.18 (112.54,240.76) | 182.22 (120.02,256.57) | 0.13 (0.03,0.23) | 62.51 (41.22,88.35) | 70.65 (46.24,99.96) | 0.41 (0.35,0.47) |
| Central Asia | 44.37 (29.83,62.19) | 39.62 (26.51,55.42) | 0.06 (-0.12,0.24) | 48.59 (32.48,68.48) | 44.22 (29.54,62.1) | 0.15 (-0.04,0.34) | 35.26 (23.59,49.92) | 31.06 (20.48,44.2) | -0.03 (-0.19,0.12) |
| Central Europe | 237.83 (157.99,335.07) | 117.66 (75.58,171.63) | -2.44 (-2.55,-2.33) | 296.36 (196.25,417.06) | 129.61 (83.41,188.8) | -2.86 (-2.99,-2.72) | 125.28 (82.82,177.64) | 90.19 (57.32,132.54) | -1.17 (-1.24,-1.1) |
| Central Latin America | 169.59 (112.34,240.35) | 99.08 (66.2,138.93) | -1.62 (-1.76,-1.48) | 217.05 (143.31,307.91) | 121.47 (81.34,170.26) | -1.74 (-1.89,-1.59) | 116.57 (77.35,165.83) | 69.23 (46.03,97.79) | -1.59 (-1.72,-1.46) |
| Central Sub-Saharan Africa | 63.64 (41.02,92.53) | 80.02 (52.07,114.37) | 0.77 (0.69,0.85) | 82.84 (52.78,121.27) | 98.7 (63.63,141.84) | 0.6 (0.49,0.71) | 42.06 (26.08,63.03) | 48.22 (31.01,69.91) | 0.47 (0.4,0.55) |
| East Asia | 108.25 (69.94,157.36) | 96.32 (61.41,142.44) | -0.99 (-1.54,-0.44) | 135.3 (87.83,195.73) | 116.23 (74.46,170.98) | -1.08 (-1.62,-0.54) | 66.86 (42.82,98.72) | 65.13 (40.91,98.41) | -0.67 (-1.19,-0.14) |
| Eastern Europe | 82.27 (54.9,117.57) | 61.49 (40.04,88.53) | -1.06 (-1.39,-0.74) | 86.02 (57.42,123.16) | 60.96 (39.89,87.69) | -1.3 (-1.6,-1) | 66.43 (43.72,95.95) | 57.84 (36.8,84.46) | -0.4 (-0.82,0.01) |
| Eastern Sub-Saharan Africa | 66.33 (42.84,96.65) | 82.24 (53.92,117.42) | 0.78 (0.72,0.85) | 88.4 (56.95,129.32) | 106.89 (70,152.41) | 0.68 (0.61,0.74) | 41.3 (26.3,60.85) | 49.59 (32.31,71.48) | 0.71 (0.64,0.78) |
| High-income Asia Pacific | 151.87 (99.41,218.55) | 147.59 (95.03,213.37) | -0.18 (-0.26,-0.1) | 167.94 (110.24,241.75) | 156.32 (100.75,225.46) | -0.35 (-0.41,-0.29) | 118.46 (77.15,170.82) | 130.6 (83.88,189.49) | 0.33 (0.2,0.46) |
| High-income North America | 182.37 (117.32,267.14) | 275.09 (176.07,398.59) | 1.56 (1.4,1.71) | 221.05 (141.87,325.14) | 344.94 (220.28,499.6) | 1.68 (1.53,1.83) | 116.41 (74.53,170.25) | 184.89 (118.78,268.8) | 1.78 (1.62,1.94) |
| North Africa and Middle East | 59.78 (39.39,84.2) | 63.14 (41.85,88.47) | 0.24 (0.09,0.4) | 79.79 (52.43,113.12) | 87.43 (57.65,122.35) | 0.42 (0.27,0.58) | 37.23 (24.71,52.41) | 37.94 (25.1,53.98) | 0.01 (-0.09,0.11) |
| Oceania | 107.64 (69.48,155.74) | 159.23 (103.25,225.15) | 1.18 (1.07,1.29) | 187.58 (120.33,273.73) | 289.45 (187.7,411.63) | 1.33 (1.21,1.45) | 19.68 (12.64,28.68) | 22.96 (14.73,32.9) | 0.52 (0.5,0.55) |
| South Asia | 225.39 (142.89,332.69) | 244.51 (158.88,349.19) | 0.2 (0.06,0.33) | 349.55 (221.23,517.03) | 356.76 (231.67,509.08) | -0.01 (-0.14,0.11) | 107.24 (67.88,158.73) | 113.54 (73.52,162.89) | 0.05 (-0.11,0.21) |
| Southeast Asia | 118.32 (77.08,169.99) | 114.54 (75.65,161.29) | -0.25 (-0.29,-0.2) | 163.32 (106.4,234.76) | 149.42 (98.66,210.51) | -0.47 (-0.54,-0.4) | 55.39 (35.75,80.48) | 61.57 (40.58,87.73) | 0.27 (0.25,0.3) |
| Southern Latin America | 226.51 (152.2,315.01) | 164.92 (111.62,227.93) | -0.99 (-1.09,-0.9) | 286.81 (192.43,401) | 208.9 (140.68,290.36) | -0.97 (-1.08,-0.85) | 130.17 (87.4,181.72) | 93.5 (63.09,130.57) | -1.13 (-1.19,-1.06) |
| Southern Sub-Saharan Africa | 43.61 (28.56,62.7) | 30.88 (20.17,44.3) | -1.46 (-1.56,-1.35) | 50.3 (32.91,72.54) | 34.51 (22.45,49.85) | -1.6 (-1.73,-1.47) | 31.35 (20.38,45.51) | 23.22 (14.98,33.9) | -1.19 (-1.25,-1.14) |
| Tropical Latin America | 152.98 (100.53,219.88) | 138.59 (92.92,195.75) | -0.27 (-0.35,-0.2) | 184.31 (120.94,266.18) | 168.3 (112.48,238.55) | -0.21 (-0.26,-0.17) | 108.58 (71.44,156.23) | 93.03 (62.24,131.58) | -0.55 (-0.7,-0.4) |
| Western Europe | 297.32 (198.86,419.64) | 275.02 (181.78,393.31) | 0.08 (-0.04,0.2) | 373.12 (248.94,526.42) | 349.46 (230.26,499.89) | 0.13 (-0.01,0.26) | 150.57 (99.36,214.42) | 168.87 (111.28,243.61) | 0.72 (0.61,0.83) |
| Western Sub-Saharan Africa | 81.92 (52.89,119.33) | 94.44 (61.78,135.16) | 0.45 (0.41,0.49) | 108.47 (69.83,157.9) | 126.82 (82.95,181.15) | 0.52 (0.48,0.56) | 48.7 (31.23,71.77) | 57.12 (37.07,82.37) | 0.55 (0.5,0.61) |
| Afghanistan | 36.99 (23.36,54.21) | 43.35 (27.79,62.33) | 0.33 (0.2,0.46) | 50.5 (31.14,75.39) | 58.87 (37.03,86.19) | 0.3 (0.15,0.44) | 26.07 (16.1,39.31) | 29.22 (18.59,42.74) | 0.26 (0.15,0.37) |
| Albania | 65.06 (42.51,93.45) | 41.56 (26.57,59.86) | -1.65 (-1.87,-1.42) | 81.61 (52.56,117.77) | 51.29 (32.93,74.61) | -1.64 (-1.86,-1.42) | 37.94 (24.24,56.02) | 29.39 (18.61,42.81) | -1.02 (-1.24,-0.8) |
| Algeria | 54.14 (35.57,77.84) | 51.95 (34.29,73.63) | -0.23 (-0.35,-0.11) | 68.96 (44.31,100.64) | 73.45 (48.29,104.01) | 0.24 (0.15,0.32) | 43.48 (27.88,62.96) | 36.81 (23.81,53.35) | -0.63 (-0.71,-0.55) |
| American Samoa | 66.31 (43.52,94.57) | 78.48 (51.31,111.4) | 0.71 (0.62,0.8) | 72.96 (46.85,105.6) | 94.36 (60.86,134.76) | 1.01 (0.95,1.08) | 55.83 (35.08,82.83) | 59.28 (37.97,85.9) | 0.26 (0.06,0.47) |
| Andorra | 395.07 (255.24,568.07) | 504.25 (336.69,720.91) | 0.84 (0.78,0.89) | 587.76 (379.11,844.08) | 774.15 (513.46,1108.34) | 0.89 (0.85,0.93) | 176.07 (112.51,256.84) | 194.86 (127.32,281.35) | 0.23 (0.13,0.33) |
| Angola | 56.23 (35.06,83.38) | 81.14 (52.59,116.39) | 1.32 (1.27,1.37) | 70.68 (43.47,105.69) | 99.61 (63.83,144.95) | 1.28 (1.22,1.34) | 38.81 (23.17,60.38) | 52.02 (32.08,77.21) | 1.03 (1,1.06) |
| Antigua and Barbuda | 43.75 (28.46,63.07) | 51.23 (33.39,72.88) | 0.66 (0.57,0.74) | 48.82 (31.36,71.52) | 61.99 (39.93,88.4) | 0.86 (0.74,0.97) | 34.84 (21.91,51.35) | 35.88 (22.65,52.94) | 0.32 (0.24,0.4) |
| Argentina | 215.05 (143.13,300.56) | 143.25 (97.25,198.48) | -1.35 (-1.46,-1.24) | 277.37 (183.7,389.73) | 183.08 (122.89,255.74) | -1.35 (-1.47,-1.23) | 115.42 (76.31,162.23) | 79.08 (52.92,110.7) | -1.36 (-1.45,-1.28) |
| Armenia | 79.2 (52.9,111.63) | 36.48 (23.88,52.51) | -2.44 (-2.94,-1.94) | 94.12 (62.28,134.48) | 45.32 (29.33,66.13) | -2.14 (-2.76,-1.52) | 53.31 (34.68,76.2) | 22.34 (14.15,32.74) | -3.12 (-3.43,-2.82) |
| Australia | 214.61 (141.8,306.69) | 345.94 (231.17,492.65) | 1.93 (1.76,2.11) | 265.77 (174.48,380.61) | 419.07 (276.52,596.59) | 1.86 (1.71,2.02) | 124.57 (80.35,179.3) | 251.4 (166.06,358.12) | 2.67 (2.42,2.92) |
| Austria | 335.37 (222.68,472.49) | 304.25 (199.62,435.04) | 0.28 (0.07,0.49) | 401.44 (265.9,567.76) | 359.22 (235.61,514.04) | 0.29 (0.04,0.54) | 184.39 (121.05,262.1) | 219.1 (143.36,314.77) | 1.05 (0.87,1.23) |
| Azerbaijan | 34 (22.04,49.9) | 26.13 (16.79,38.2) | -0.71 (-0.83,-0.59) | 37.5 (23.49,56.55) | 29.21 (18.24,44.09) | -0.66 (-0.78,-0.53) | 25.88 (16.12,38.95) | 21.17 (12.96,31.73) | -0.48 (-0.6,-0.37) |
| Bahamas | 65.61 (43.12,92.97) | 71.67 (47.15,101.48) | 0.14 (0,0.27) | 79.6 (51.89,113.96) | 90.71 (59.35,129.38) | 0.3 (0.16,0.45) | 39.39 (24.54,57.86) | 42.57 (26.93,61.97) | 0.14 (0.08,0.19) |
| Bahrain | 36.41 (23.3,53.14) | 29.55 (18.84,42.89) | -0.75 (-0.82,-0.67) | 40.04 (24.73,60.01) | 35.47 (22.33,52.17) | -0.47 (-0.56,-0.38) | 30.26 (18.47,45.34) | 22.17 (13.89,33) | -1.09 (-1.16,-1.03) |
| Bangladesh | 23.66 (14.51,35.44) | 32.55 (21.02,46.89) | 1.11 (0.72,1.5) | 29.83 (17.26,46.6) | 38.59 (24.15,56.7) | 0.9 (0.41,1.4) | 18.93 (10.51,29.91) | 27.12 (16.61,40.61) | 1.23 (0.97,1.49) |
| Barbados | 50.42 (32.56,73.18) | 57.44 (37.52,82.05) | 0.5 (0.39,0.6) | 64.09 (40.84,94.32) | 79.94 (51.79,114.61) | 0.76 (0.66,0.86) | 26.36 (16.47,39.19) | 26.69 (16.71,39.25) | 0.18 (0.07,0.29) |
| Belarus | 67.89 (45.44,95.57) | 64.84 (41.2,94.89) | -0.25 (-0.8,0.31) | 71.4 (47.29,101.76) | 64.35 (40.97,93.81) | -0.57 (-1.09,-0.04) | 57.7 (37.82,82.5) | 59.95 (36.91,89.79) | 0.31 (-0.32,0.95) |
| Belgium | 301.92 (200.03,426.36) | 413.85 (274.33,587.5) | 1.33 (1.16,1.49) | 393.89 (260.24,558.23) | 553.53 (364.74,786.93) | 1.42 (1.26,1.58) | 132 (86.95,189.83) | 221.81 (146.03,320.36) | 2.02 (1.81,2.24) |
| Belize | 51.38 (33.21,73.68) | 62.69 (41.27,89.26) | 0.49 (0.36,0.63) | 65.57 (41.13,95.61) | 78.1 (50.99,112.48) | 0.45 (0.3,0.6) | 34.23 (21.49,50.26) | 46.72 (30.4,66.94) | 0.82 (0.69,0.95) |
| Benin | 75.76 (48.29,111.09) | 89.76 (58.91,130.07) | 0.41 (0.36,0.46) | 98.71 (62.23,145.79) | 110.92 (71.6,161.1) | 0.21 (0.16,0.26) | 50.77 (31.26,77.28) | 58.65 (36.6,87.16) | 0.34 (0.29,0.39) |
| Bermuda | 53.4 (34.62,77.54) | 46.47 (29.43,68.23) | -0.74 (-0.85,-0.64) | 57.27 (36.77,83.96) | 47.17 (29.64,69.72) | -0.97 (-1.06,-0.87) | 45.39 (28.72,66.59) | 44.61 (28.02,66.98) | -0.24 (-0.41,-0.07) |
| Bhutan | 108.37 (68.94,158.94) | 216.16 (140.74,308.95) | 2.26 (1.97,2.56) | 139.51 (87.89,206.99) | 304.92 (196.37,437.65) | 2.55 (2.18,2.92) | 65.93 (39.88,98.57) | 124.38 (79.45,180.88) | 2.1 (1.86,2.34) |
| Bolivia (Plurinational State of) | 69.85 (44.83,102.26) | 84.4 (55.51,120.82) | 0.62 (0.54,0.7) | 77.35 (48.39,115.02) | 96.62 (62.65,139.84) | 0.77 (0.65,0.89) | 58.68 (36.12,88.11) | 67.97 (43.38,99.05) | 0.43 (0.39,0.46) |
| Bosnia and Herzegovina | 104.13 (68.57,149.01) | 66.43 (42.9,95.38) | -1.42 (-1.62,-1.22) | 111.38 (72.95,160.77) | 68.39 (43.91,98.28) | -1.57 (-1.76,-1.38) | 90.48 (58.76,131.17) | 61.76 (39.39,90.08) | -1.16 (-1.36,-0.95) |
| Botswana | 51.52 (32.31,76.18) | 50.81 (32.59,74.8) | -0.01 (-0.09,0.06) | 59.19 (36.12,88.71) | 56.72 (35.63,84.92) | -0.19 (-0.27,-0.1) | 37.42 (22.47,57.81) | 37.45 (22.76,57.01) | 0.16 (0.02,0.3) |
| Brazil | 154.71 (101.56,222.56) | 139.67 (93.61,197.34) | -0.29 (-0.36,-0.22) | 186.5 (122.28,269.47) | 169.57 (113.23,240.45) | -0.23 (-0.28,-0.18) | 109.57 (72.02,157.67) | 93.78 (62.71,132.7) | -0.56 (-0.71,-0.4) |
| Brunei Darussalam | 195.77 (130.36,278.69) | 208.32 (140.13,291.57) | 0.22 (0.17,0.27) | 232.17 (153.18,331.27) | 244.31 (163.02,342.72) | 0.22 (0.11,0.34) | 153.04 (100.38,223.39) | 158.03 (105.75,222.98) | 0.11 (0.06,0.16) |
| Bulgaria | 70.87 (46.98,100.96) | 57.44 (37.61,81.93) | -0.46 (-0.58,-0.34) | 74.1 (48.45,107.31) | 61.15 (39.54,87.51) | -0.31 (-0.44,-0.17) | 65.77 (42.91,94.87) | 49.85 (31.73,72.79) | -0.82 (-0.94,-0.69) |
| Burkina Faso | 84.39 (53.68,122.33) | 97.07 (62.59,139.69) | 0.4 (0.33,0.47) | 115.45 (72.37,169.29) | 127.53 (81.72,185.2) | 0.26 (0.19,0.33) | 47.46 (28.98,72.21) | 56.78 (35.14,84.36) | 0.59 (0.53,0.65) |
| Burundi | 69.74 (43.56,103.7) | 75.54 (48.44,109.19) | 0.49 (0.35,0.63) | 89.61 (55.11,135.98) | 98.35 (62.05,142.79) | 0.48 (0.34,0.61) | 43.63 (25.66,67.81) | 49.82 (30.62,74.14) | 0.8 (0.65,0.96) |
| Cabo Verde | 70.89 (46.16,101.67) | 80.96 (53.19,115.39) | 0.43 (0.4,0.46) | 86.44 (55.12,125.32) | 88.87 (57.39,127.51) | 0.09 (0.05,0.13) | 49.09 (30.67,73) | 62.18 (39.58,91.24) | 0.8 (0.71,0.89) |
| Cambodia | 113.54 (72.11,164.22) | 191.78 (124.15,275.11) | 1.86 (1.8,1.92) | 142.56 (89.69,208.66) | 234.61 (151.15,338.74) | 1.77 (1.71,1.82) | 66.48 (40.6,99.56) | 109.51 (69.23,158.04) | 1.83 (1.75,1.9) |
| Cameroon | 86.03 (55.4,125.06) | 95.89 (62.05,138.97) | 0.2 (0.1,0.29) | 112.2 (71.77,164.73) | 119.76 (76.15,175.2) | 0.05 (-0.06,0.15) | 56.84 (34.58,84.93) | 63.7 (39.12,95.23) | 0.24 (0.14,0.34) |
| Canada | 237.59 (155.41,342.27) | 336.47 (223.45,479.26) | 1.42 (1.22,1.61) | 288.03 (187.63,416.23) | 417.78 (277.03,595.48) | 1.51 (1.31,1.71) | 151.63 (98.48,220.28) | 228.81 (150.98,330.07) | 1.65 (1.48,1.83) |
| Central African Republic | 56.16 (34.98,84.1) | 57.16 (35.47,84.67) | 0.05 (-0.04,0.13) | 67.79 (41.57,102.75) | 67.41 (41.26,102.01) | -0.02 (-0.09,0.06) | 37.14 (21.61,57.82) | 37.16 (21.47,57.57) | -0.01 (-0.08,0.06) |
| Chad | 66.31 (41.92,97.81) | 71.55 (46.19,103.54) | 0.23 (0.18,0.29) | 85.82 (53.36,127.01) | 95.93 (61.06,140.2) | 0.31 (0.26,0.36) | 43.15 (26.02,67.1) | 49.58 (30.57,74.27) | 0.46 (0.4,0.53) |
| Chile | 261.42 (176.83,364.31) | 206.06 (136.34,292.24) | -0.6 (-0.68,-0.51) | 313.47 (211.56,438.63) | 260.07 (170.58,367.66) | -0.4 (-0.5,-0.3) | 181.88 (120.65,257.95) | 122.8 (81.09,175.31) | -1.2 (-1.27,-1.13) |
| China | 110.09 (71.06,160.23) | 98.9 (63.03,146.37) | -0.97 (-1.53,-0.41) | 137.94 (89.52,199.94) | 119.48 (76.48,175.85) | -1.07 (-1.61,-0.52) | 67.49 (43.17,99.95) | 66.7 (41.85,100.91) | -0.63 (-1.16,-0.1) |
| Colombia | 114.75 (76.07,162.03) | 55.96 (36.98,78.85) | -2.3 (-2.5,-2.09) | 147.23 (96.21,209.84) | 66.63 (43.26,94.68) | -2.58 (-2.82,-2.35) | 76.74 (49.91,110.12) | 41 (26.65,58.72) | -1.91 (-2.06,-1.77) |
| Comoros | 55.76 (35.54,81.68) | 68.02 (44.17,98.61) | 0.61 (0.54,0.68) | 71.33 (44.62,105.81) | 87.01 (55.64,127.25) | 0.56 (0.49,0.64) | 36.39 (21.9,55.15) | 43.68 (27.06,64.95) | 0.65 (0.56,0.74) |
| Congo | 67.38 (42.32,99.03) | 72.66 (46.52,106.53) | 0.22 (0.12,0.32) | 82.39 (51.05,123.15) | 91.06 (57.37,134.92) | 0.28 (0.17,0.39) | 45.09 (26.77,69.07) | 48.58 (30.31,72.59) | 0.28 (0.17,0.38) |
| Cook Islands | 48.18 (31.37,69.37) | 49.53 (32.44,70.79) | 0.12 (0.06,0.18) | 54.32 (34.44,79.26) | 53.92 (34.66,77.29) | -0.01 (-0.14,0.11) | 40.04 (25.37,59.82) | 42.64 (27.23,62.24) | 0.31 (0.23,0.39) |
| Costa Rica | 175.81 (116.89,247.15) | 117.18 (78.27,163.85) | -1.32 (-1.41,-1.24) | 249.2 (164.64,351.58) | 155.37 (103.48,218.77) | -1.55 (-1.64,-1.45) | 93.71 (61.57,134.25) | 65.75 (42.78,93.04) | -1.04 (-1.14,-0.94) |
| Croatia | 233.61 (152.12,331.78) | 267.13 (169.42,392.17) | 0.56 (0.49,0.64) | 287.51 (186.55,408.6) | 324.04 (205.49,472.7) | 0.58 (0.46,0.69) | 117.02 (75.1,171.36) | 155.08 (96.71,232.86) | 0.85 (0.76,0.93) |
| Cuba | 198.87 (130.43,280.89) | 237.78 (155.14,337.08) | 0.5 (0.37,0.62) | 302.97 (197.59,429.49) | 335.87 (219.27,476.51) | 0.27 (0.16,0.39) | 88.93 (57.23,127.86) | 108.35 (69.16,155.88) | 0.66 (0.58,0.74) |
| Cyprus | 347.9 (230.85,488.47) | 292.97 (192.41,422.77) | -0.3 (-0.45,-0.15) | 471.78 (311.86,667.62) | 393.79 (258.42,570.76) | -0.47 (-0.61,-0.34) | 171.2 (113.29,242.72) | 154.22 (100.62,222.88) | -0.21 (-0.31,-0.11) |
| Czechia | 440.43 (290.21,617.32) | 163.8 (104.82,240.61) | -3.36 (-3.6,-3.12) | 562.18 (371.13,786.57) | 184.79 (118.01,270.01) | -3.78 (-4.05,-3.52) | 175.29 (112.97,253.76) | 121.42 (76.98,180.77) | -1.32 (-1.44,-1.19) |
| C?te d'Ivoire | 76.3 (49.56,110.26) | 88.05 (57.61,127.2) | 0.39 (0.3,0.47) | 99.32 (62.82,145.31) | 111.43 (71.66,161.99) | 0.29 (0.17,0.41) | 51.89 (31.81,78.08) | 59.92 (37.53,89.43) | 0.44 (0.37,0.51) |
| Democratic People's Republic of Korea | 60.36 (38.71,87.51) | 34.89 (22.76,50.37) | -1.07 (-1.33,-0.81) | 67.71 (42.69,99.24) | 41.09 (26.38,59.84) | -0.93 (-1.18,-0.68) | 41.95 (25.76,62.87) | 19.79 (12.34,29.71) | -1.81 (-2.06,-1.57) |
| Democratic Republic of the Congo | 64.17 (40.96,94.52) | 80.97 (52.23,116.2) | 0.77 (0.68,0.86) | 85.67 (53.25,127.22) | 100.4 (63.45,146.12) | 0.54 (0.4,0.68) | 42.18 (25.49,64.77) | 46.84 (29.01,69.42) | 0.35 (0.26,0.45) |
| Denmark | 453.41 (304.62,635.94) | 253.47 (168.96,362.59) | -2.09 (-2.21,-1.97) | 595.26 (400.24,841.53) | 335.1 (222.11,479.25) | -2.06 (-2.2,-1.93) | 211.85 (137.91,300.53) | 139.41 (91.41,201.02) | -1.55 (-1.64,-1.46) |
| Djibouti | 62.17 (39.15,91.96) | 72.09 (46.82,104.84) | 0.41 (0.37,0.44) | 79.68 (49.21,119.25) | 93.65 (59.57,135.74) | 0.4 (0.37,0.44) | 38.55 (23.17,59.89) | 47.13 (29.75,70.8) | 0.68 (0.64,0.73) |
| Dominica | 41.22 (26.33,60.15) | 47.84 (30.55,68.43) | 0.45 (0.33,0.57) | 41.59 (25.75,61.95) | 48.17 (30.27,70.03) | 0.47 (0.31,0.64) | 40.58 (25.2,60.46) | 46.37 (29.11,67.97) | 0.34 (0.26,0.42) |
| Dominican Republic | 56.02 (36.7,81.06) | 62.61 (41.13,88.47) | 0.21 (0.13,0.29) | 77.31 (49.66,114.47) | 81.65 (53.2,116.56) | 0.02 (-0.06,0.1) | 33.97 (21.59,49.91) | 38.93 (24.69,56.5) | 0.27 (0.18,0.36) |
| Ecuador | 78.88 (52.11,111.43) | 77.64 (51.87,109.22) | -0.2 (-0.29,-0.11) | 84.53 (54.58,121.93) | 85.6 (56.43,122.6) | -0.05 (-0.13,0.03) | 71.61 (46.37,103.66) | 65.51 (43.01,93.12) | -0.5 (-0.61,-0.39) |
| Egypt | 31.45 (19.87,45.82) | 30.95 (20.04,44.58) | -0.21 (-0.32,-0.09) | 49.7 (30.56,73.93) | 56.62 (36.36,83.05) | 0.25 (0.17,0.33) | 16.59 (10.12,24.81) | 16.51 (10.26,24.39) | -0.08 (-0.21,0.05) |
| El Salvador | 117.49 (77.65,165.63) | 103.47 (68.49,145.38) | -0.39 (-0.43,-0.35) | 145.86 (95.31,209.01) | 128.02 (84.24,181.02) | -0.39 (-0.43,-0.34) | 82.12 (53.42,117.7) | 67.68 (44.24,96.69) | -0.61 (-0.69,-0.54) |
| Equatorial Guinea | 53.84 (33.74,79.84) | 77.05 (49.93,110.57) | 1.21 (1.1,1.33) | 65.44 (40.47,99.39) | 93.43 (59.95,135.23) | 1.21 (1.12,1.31) | 36.57 (21.48,57.03) | 51.7 (31.96,76.16) | 1.31 (1.18,1.44) |
| Eritrea | 46.86 (28.61,70.57) | 75.94 (48.91,110.31) | 1.1 (0.9,1.31) | 56.24 (34.03,85.75) | 91.95 (58.32,133.43) | 1.12 (0.93,1.31) | 26.5 (15.54,41.22) | 42.15 (26.14,63.35) | 1.13 (0.93,1.34) |
| Estonia | 137.09 (91.29,192.53) | 60.5 (38.6,88.75) | -3.05 (-3.29,-2.82) | 150.05 (99.63,211) | 55.78 (35.4,81.65) | -3.74 (-4.01,-3.47) | 94.98 (61.98,135.73) | 62.93 (39.44,93.59) | -1.46 (-1.72,-1.21) |
| Eswatini | 41.64 (25.9,61.43) | 39.06 (24.5,57.52) | -0.44 (-0.64,-0.24) | 47.17 (28.58,71.02) | 42.4 (25.83,63.64) | -0.54 (-0.71,-0.37) | 28.91 (17.34,44.06) | 28.71 (17.35,43.43) | -0.28 (-0.52,-0.04) |
| Ethiopia | 72.24 (45.45,108.79) | 83.32 (53.52,120.35) | 0.52 (0.44,0.61) | 98.47 (61.62,150.24) | 117.21 (75.12,169.22) | 0.59 (0.52,0.66) | 43.14 (26.51,66.47) | 50.39 (32.03,73.34) | 0.6 (0.51,0.69) |
| Fiji | 43.95 (27.65,64.54) | 51.56 (33.14,74.25) | 0.64 (0.54,0.74) | 54.58 (33.88,81.26) | 61.41 (38.92,89.08) | 0.54 (0.44,0.64) | 26.86 (15.8,41.61) | 30.23 (18.03,46.03) | 0.48 (0.37,0.58) |
| Finland | 413.78 (279.18,576.4) | 380.36 (250.49,541.68) | 0.01 (-0.26,0.29) | 482.2 (324.85,674.64) | 440.6 (289.24,628.84) | 0 (-0.27,0.27) | 252.82 (168.08,355.2) | 287.47 (188.6,410.95) | 0.71 (0.41,1.01) |
| France | 434.21 (289.82,604.79) | 372.88 (246.52,530.75) | -0.05 (-0.2,0.1) | 542.92 (360.66,756.5) | 470.67 (310.38,668.86) | -0.05 (-0.19,0.1) | 215.9 (142.85,305.35) | 226.34 (148.31,325.32) | 0.71 (0.53,0.88) |
| Gabon | 88.48 (56.68,129.53) | 88 (57.54,125.98) | -0.08 (-0.16,0.01) | 107.59 (67.81,158.52) | 101.86 (65.77,147.54) | -0.28 (-0.38,-0.18) | 58.33 (35.7,88.37) | 62.01 (39.04,91.05) | 0.16 (0.12,0.21) |
| Gambia | 80.94 (51.72,118.17) | 101.81 (66.23,146.63) | 0.62 (0.6,0.65) | 104.99 (66.03,154.62) | 131.64 (84.93,189.92) | 0.58 (0.54,0.63) | 47.73 (29.11,73.26) | 60.23 (37.31,91.73) | 0.66 (0.63,0.69) |
| Georgia | 50.36 (32.92,72.13) | 79.49 (52.73,112.14) | 2.39 (1.97,2.81) | 53.4 (34.35,77.9) | 88.57 (58.29,126.11) | 2.54 (2.15,2.92) | 42.79 (27.58,62.93) | 60.83 (39.26,88.72) | 2.05 (1.58,2.53) |
| Germany | 294.74 (198.19,411.21) | 282.84 (187.04,404.15) | 0.4 (0.15,0.65) | 363.16 (243.24,508.63) | 362.24 (237.91,518.39) | 0.56 (0.29,0.83) | 141.62 (92.98,201.11) | 170.08 (111.43,244.71) | 1.13 (0.94,1.32) |
| Ghana | 84.54 (53.99,123.82) | 97.45 (62.23,141.12) | 0.28 (0.24,0.32) | 114.65 (72.03,170.53) | 120.5 (76.23,174.87) | -0.08 (-0.14,-0.02) | 48.59 (29.39,73.34) | 59.63 (36.37,89.26) | 0.61 (0.58,0.63) |
| Greece | 160.56 (104.37,233.72) | 110.2 (71.5,156.76) | -0.95 (-1.14,-0.77) | 214.67 (139.23,312.86) | 137.11 (87.87,195.66) | -1.17 (-1.4,-0.94) | 90.28 (58.46,132.16) | 75.71 (49.13,109.1) | -0.39 (-0.48,-0.3) |
| Greenland | 709.01 (471.47,994.81) | 545.42 (368.68,760.05) | -0.77 (-0.83,-0.71) | 909.94 (604.16,1284.68) | 713.83 (479.47,995.66) | -0.66 (-0.74,-0.57) | 395.17 (261.74,561.11) | 352.96 (235.43,495.37) | -0.34 (-0.39,-0.29) |
| Grenada | 52.09 (33.45,75.63) | 71.77 (46.89,102.37) | 1.04 (0.93,1.15) | 52.76 (32.9,78.22) | 72.25 (46.31,104.58) | 1.05 (0.94,1.15) | 49.86 (31.26,73.59) | 59.88 (38.55,87.8) | 0.37 (0.18,0.56) |
| Guam | 53.83 (34.87,77.56) | 51.64 (33.76,73.47) | 0 (-0.09,0.09) | 65.71 (42.01,95.69) | 61.82 (39.82,88.48) | -0.1 (-0.2,0.01) | 40.01 (24.98,59.5) | 35.75 (22.5,53.39) | -0.12 (-0.31,0.06) |
| Guatemala | 117.97 (77.2,168.36) | 120.07 (79.81,169.03) | -0.24 (-0.34,-0.14) | 137.79 (87.46,200.08) | 136.54 (89.71,194.4) | -0.32 (-0.42,-0.23) | 94.63 (60.05,138.17) | 99.5 (65,141.53) | -0.09 (-0.21,0.03) |
| Guinea | 66.53 (41.78,98.08) | 75.61 (48.78,108.56) | 0.26 (0.21,0.32) | 89.96 (55.57,133.65) | 98.94 (63.07,143.55) | 0.09 (0.03,0.15) | 43.97 (26.37,68.23) | 51.21 (31.44,76.44) | 0.42 (0.36,0.47) |
| Guinea-Bissau | 70.86 (44.36,105.77) | 85.78 (54.94,126.06) | 0.52 (0.47,0.56) | 89.81 (55.21,134.77) | 104.64 (66.27,155.38) | 0.35 (0.29,0.4) | 48.37 (29.11,74.28) | 56.4 (34.24,85.97) | 0.44 (0.4,0.47) |
| Guyana | 99.54 (63.95,143.74) | 112.23 (73.13,159.87) | 0.17 (0.07,0.28) | 122.33 (77.92,177.62) | 137.34 (87.76,196.43) | 0.2 (0.09,0.31) | 71.73 (44.47,107.18) | 77.51 (49.42,113.45) | -0.02 (-0.12,0.07) |
| Haiti | 60.13 (37.69,88.85) | 58.72 (37.62,85.44) | -0.46 (-0.93,0.02) | 76.46 (46.76,114.58) | 75.33 (47.55,111.2) | -0.39 (-0.81,0.04) | 36.07 (21.69,55.17) | 41.4 (25.56,61.53) | 0.08 (-0.44,0.6) |
| Honduras | 66.17 (42.43,96.44) | 63.66 (41.82,91.77) | -0.25 (-0.33,-0.18) | 82.47 (51.35,122.04) | 76.53 (49.22,112.13) | -0.23 (-0.32,-0.14) | 45.68 (28.5,68.05) | 48.34 (30.95,71.33) | -0.11 (-0.23,0.01) |
| Hungary | 497.39 (329.18,699.34) | 168.7 (106.79,247.25) | -4.04 (-4.25,-3.82) | 621.03 (411.04,874.54) | 188.31 (118.8,276.44) | -4.41 (-4.63,-4.19) | 246.54 (159.74,355.17) | 121.97 (76.37,182.37) | -2.77 (-2.97,-2.58) |
| Iceland | 218.21 (142.83,312.93) | 228.99 (152.48,325.09) | 0.21 (0.16,0.25) | 287.59 (187.27,410.94) | 281.4 (185.21,401.04) | -0.09 (-0.12,-0.06) | 123.04 (80.29,178.4) | 160.91 (105.9,231.65) | 1.07 (0.97,1.18) |
| India | 281.1 (178.1,415.52) | 289.05 (187.67,413.13) | 0.01 (-0.08,0.1) | 424.43 (268.43,627.99) | 415.09 (269.22,593.2) | -0.18 (-0.26,-0.09) | 133.23 (84.23,197.79) | 134.04 (86.55,192.5) | -0.12 (-0.23,-0.01) |
| Indonesia | 143.55 (92.27,209.53) | 122.84 (79.81,176.15) | -0.87 (-1,-0.75) | 218.04 (139.61,319.33) | 172.72 (112.03,248.51) | -1.17 (-1.31,-1.03) | 52.89 (33.51,78.46) | 55.88 (35.96,81.15) | -0.06 (-0.15,0.02) |
| Iran (Islamic Republic of) | 68.14 (44.48,98.74) | 45.9 (29.82,66.28) | -1.32 (-1.39,-1.25) | 83.83 (54.34,122.35) | 58.7 (38.14,85.09) | -1.08 (-1.16,-1) | 48.71 (31.95,70.19) | 34.24 (22.29,49.49) | -1.3 (-1.43,-1.17) |
| Iraq | 43.42 (28.68,62.92) | 31.36 (20.69,45.41) | -1.31 (-1.44,-1.19) | 55.84 (35.9,82.01) | 37.52 (24.17,54.81) | -1.58 (-1.73,-1.43) | 28.83 (18.37,41.8) | 23.25 (15.05,33.89) | -0.88 (-0.97,-0.79) |
| Ireland | 223.67 (148.31,315.49) | 210.31 (138.27,299.73) | 0.03 (-0.14,0.19) | 303.59 (200.5,430.57) | 276.63 (181.11,393.88) | -0.09 (-0.22,0.04) | 96.56 (63.47,137.98) | 124.35 (81.41,178.59) | 1.15 (0.87,1.43) |
| Israel | 176.11 (117.42,246.84) | 138.08 (90.92,197.56) | -0.59 (-0.72,-0.46) | 255.05 (170.23,358.56) | 178.41 (116.02,256.28) | -0.99 (-1.13,-0.86) | 81.43 (53.85,114.74) | 83.82 (54.46,121.33) | 0.45 (0.29,0.61) |
| Italy | 369.07 (236.99,543.67) | 252.41 (163.2,365.01) | -1.27 (-1.35,-1.19) | 464.94 (298.44,684.08) | 307.86 (198.77,445.87) | -1.39 (-1.46,-1.33) | 193.12 (122.64,285.62) | 170.07 (109.44,246.73) | -0.36 (-0.48,-0.23) |
| Jamaica | 36.23 (23.4,52) | 47.5 (31.2,67.44) | 0.84 (0.71,0.96) | 46.63 (29.38,68.91) | 61.81 (40.12,88.97) | 0.79 (0.64,0.94) | 22.18 (13.81,33.33) | 27.99 (17.84,40.75) | 0.86 (0.75,0.97) |
| Japan | 133.31 (84.54,196.29) | 122.84 (78.61,178.9) | -0.34 (-0.5,-0.18) | 145.36 (92.23,214.07) | 125.53 (80.48,182.58) | -0.59 (-0.72,-0.45) | 106.5 (67.2,157.4) | 115.77 (74,169.11) | 0.28 (0.06,0.5) |
| Jordan | 45.62 (29.71,65.8) | 35.34 (22.98,50.55) | -0.96 (-1.04,-0.88) | 53.69 (34.1,78.71) | 44.36 (28.43,64.04) | -0.78 (-0.9,-0.66) | 37.68 (24.19,55.32) | 27.62 (17.63,40.16) | -1.07 (-1.18,-0.97) |
| Kazakhstan | 45.9 (30.09,66.6) | 47.54 (31.35,67.22) | 0.86 (0.61,1.12) | 50.38 (32.46,74.32) | 51.12 (33.25,73.49) | 0.74 (0.51,0.98) | 34.61 (21.77,50.8) | 38.17 (24.21,55.82) | 1.2 (0.9,1.5) |
| Kenya | 77.07 (49.33,112.85) | 92.64 (60.06,133.31) | 0.7 (0.65,0.75) | 102.57 (65.52,149.73) | 114 (73.88,164.34) | 0.4 (0.36,0.45) | 50.2 (31.93,74.55) | 56.29 (36.05,82.05) | 0.46 (0.4,0.53) |
| Kiribati | 33.55 (20.34,50.32) | 38.64 (24.13,56.61) | 0.44 (0.31,0.57) | 45.98 (27.31,70.28) | 52.65 (32.01,77.8) | 0.41 (0.28,0.55) | 12.06 (6.47,19.76) | 12.63 (6.97,20.26) | 0.07 (0,0.14) |
| Kuwait | 34.36 (22.58,49.01) | 27.94 (17.77,41.08) | -1.27 (-1.47,-1.06) | 39.92 (25.64,57.9) | 33.46 (21.03,49.72) | -1.23 (-1.53,-0.93) | 25.85 (16.68,37.54) | 23.89 (15.12,35.37) | -0.78 (-0.96,-0.6) |
| Kyrgyzstan | 46.92 (30.77,67.72) | 32.53 (21.45,46.41) | -1.21 (-1.3,-1.12) | 48.51 (30.89,71.43) | 34.72 (22.29,50.6) | -1.06 (-1.13,-0.98) | 41.56 (25.99,61.72) | 27.23 (17.33,39.69) | -1.48 (-1.61,-1.36) |
| Lao People's Democratic Republic | 54.6 (33.83,80.66) | 81.48 (52.37,115.97) | 1.43 (1.37,1.49) | 67.26 (40.52,101.07) | 104.55 (66.19,150.07) | 1.57 (1.5,1.63) | 34.9 (20.42,54.28) | 50.16 (30.89,74.96) | 1.27 (1.22,1.32) |
| Latvia | 174.89 (117.67,245.13) | 79.01 (51.32,114.41) | -3.09 (-3.43,-2.75) | 198.92 (132.66,279.96) | 79.03 (51.28,114.23) | -3.64 (-3.98,-3.31) | 111.07 (73.71,159.47) | 70.12 (44.44,103.19) | -1.56 (-1.98,-1.14) |
| Lebanon | 48.68 (32.21,69.45) | 38.1 (24.44,55.3) | -1.13 (-1.29,-0.97) | 66.07 (43.32,94.56) | 50.27 (32.24,73.2) | -1.17 (-1.36,-0.98) | 25.57 (16.5,37.37) | 23.44 (14.77,34.52) | -0.57 (-0.72,-0.42) |
| Lesotho | 44.89 (28.09,65.78) | 43.26 (26.96,64.65) | -0.26 (-0.46,-0.07) | 49.23 (30.17,73.47) | 47 (28.38,71.4) | -0.29 (-0.5,-0.07) | 31.12 (18.57,47.9) | 29.92 (17.85,45.88) | -0.29 (-0.45,-0.14) |
| Liberia | 67.42 (42.96,99.61) | 83.6 (54.3,119.71) | 0.6 (0.52,0.68) | 92.19 (58.23,137.02) | 112.29 (71.99,162.67) | 0.49 (0.41,0.57) | 46.56 (27.99,70.96) | 55.09 (34.84,80.35) | 0.48 (0.4,0.56) |
| Libya | 55.86 (36.76,79.55) | 49.15 (32.64,69.26) | -0.41 (-0.52,-0.29) | 71.49 (46.2,102.64) | 63.51 (41.68,90.4) | -0.34 (-0.44,-0.24) | 40 (25.6,57.97) | 34.13 (22.44,49.05) | -0.5 (-0.64,-0.36) |
| Lithuania | 125.59 (83.82,175.18) | 113.62 (73.98,161.24) | -0.24 (-0.41,-0.08) | 132.35 (88.24,186.36) | 117.68 (77.08,167.46) | -0.35 (-0.44,-0.26) | 109.91 (71.97,156.96) | 96.16 (61.59,138.33) | -0.25 (-0.6,0.09) |
| Luxembourg | 331.38 (221.51,462.24) | 317.66 (210.77,454.54) | 0.45 (0.25,0.65) | 410.06 (272.89,573.35) | 401.92 (267.19,578.87) | 0.54 (0.34,0.74) | 178.8 (118.29,254.66) | 193.54 (126.73,277.91) | 0.82 (0.6,1.03) |
| Madagascar | 49.38 (31.33,72.83) | 54.64 (34.84,80.6) | 0.21 (0.17,0.25) | 67.73 (41.8,101.76) | 71.13 (44.29,106.73) | 0.05 (0.01,0.1) | 31.34 (18.42,48.3) | 34.25 (20.54,51.8) | 0.13 (0.07,0.2) |
| Malawi | 66.14 (42.16,98.22) | 87.89 (56.77,127.96) | 1.08 (0.93,1.24) | 86.82 (53.7,129.57) | 106.9 (68.32,156.76) | 0.83 (0.62,1.03) | 40.94 (24.69,63.74) | 52.66 (31.89,79.55) | 1.02 (0.91,1.14) |
| Malaysia | 83.99 (54.61,120.74) | 78.94 (51.48,112.81) | -0.1 (-0.14,-0.05) | 108.2 (69.93,157.27) | 100.84 (65.62,145.51) | -0.14 (-0.18,-0.1) | 55.56 (34.64,81.93) | 57.31 (36.48,83.16) | 0.24 (0.18,0.3) |
| Maldives | 70.19 (44.23,103.09) | 79.45 (51.44,113.27) | 0.35 (0.3,0.4) | 97.72 (61.11,143.7) | 106.33 (68.71,151.99) | 0.18 (0.1,0.26) | 57.61 (35.29,86.7) | 54.52 (34.17,78.84) | -0.2 (-0.25,-0.15) |
| Mali | 90.01 (57.09,132.69) | 107.48 (69.62,155.76) | 0.49 (0.45,0.52) | 129.21 (80.55,192.79) | 157.55 (101.02,232.25) | 0.53 (0.49,0.57) | 49.46 (29.91,76.08) | 60.9 (37.75,91.12) | 0.65 (0.61,0.69) |
| Malta | 283.58 (187.89,397.2) | 221.14 (145.7,317.28) | -0.51 (-0.64,-0.38) | 386.47 (255.51,543.07) | 287.02 (188.33,411.87) | -0.68 (-0.82,-0.54) | 131.36 (86.63,186.88) | 130.79 (85.84,188.67) | 0.35 (0.21,0.49) |
| Marshall Islands | 62.01 (39.15,90.61) | 72.65 (46.64,104.49) | 0.46 (0.39,0.53) | 77.24 (47.86,113.48) | 101.25 (64.06,146.23) | 0.88 (0.76,1) | 34.99 (20.89,54.2) | 45.72 (27.99,69.59) | 0.74 (0.67,0.82) |
| Mauritania | 78.07 (49.49,114.26) | 79.76 (51.69,114.39) | -0.09 (-0.15,-0.04) | 98.81 (61.71,146.02) | 107.08 (68.6,154.91) | 0.09 (0.02,0.16) | 50.34 (30.79,75.91) | 53.7 (33.32,78.87) | 0.13 (0.07,0.18) |
| Mauritius | 32.15 (20.44,46.83) | 39.34 (25.75,56.76) | 1.49 (1.22,1.77) | 32.95 (20.21,49.16) | 40.74 (26.07,59.12) | 1.51 (1.24,1.78) | 28.22 (17.02,42.34) | 34.73 (22.15,51.29) | 1.51 (1.22,1.8) |
| Mexico | 216.81 (142.5,311.42) | 123.51 (81.72,175.24) | -1.61 (-1.81,-1.42) | 280.07 (183,402.53) | 152.61 (101.15,215.92) | -1.72 (-1.93,-1.5) | 154.6 (101.68,222.07) | 87.51 (57.76,124.84) | -1.7 (-1.87,-1.53) |
| Micronesia (Federated States of) | 62.68 (39.75,91.13) | 85.2 (55.23,122.18) | 1.09 (1.04,1.15) | 79.89 (49.81,117.74) | 106.27 (68.55,152.87) | 1.01 (0.96,1.05) | 35.12 (21.12,53.76) | 47.7 (29.42,71.37) | 1.04 (1,1.08) |
| Monaco | 150.97 (97.6,219.33) | 161.18 (105.34,231.6) | 0.23 (0.14,0.33) | 193.33 (124.57,280.33) | 212.06 (137.54,305.79) | 0.33 (0.23,0.43) | 78.76 (49.9,116.1) | 93.11 (60.25,135.18) | 0.59 (0.47,0.72) |
| Mongolia | 52.49 (32.84,77.39) | 55.25 (36.01,79.79) | 0.34 (0.24,0.44) | 61.55 (37.58,92.54) | 62.15 (40.12,90.76) | 0.18 (0.07,0.3) | 39.02 (23.66,59.85) | 43.55 (27.18,63.94) | 0.6 (0.51,0.7) |
| Montenegro | 120.85 (78.86,172.27) | 81.28 (51.05,120.7) | -1.61 (-1.76,-1.47) | 127.13 (82.08,181.58) | 80.46 (50.53,120.19) | -1.79 (-1.94,-1.64) | 107.99 (69.96,156.97) | 79.21 (48.96,117.73) | -1.28 (-1.41,-1.15) |
| Morocco | 81.88 (52.63,117.92) | 95.74 (62.69,136.26) | 0.4 (0.34,0.46) | 129.32 (81.82,187.85) | 147.43 (95.63,210.81) | 0.31 (0.24,0.39) | 33.93 (21.35,49.79) | 37.59 (24.32,55.05) | 0.29 (0.26,0.32) |
| Mozambique | 66.49 (41.82,98.98) | 81.76 (52.43,119.42) | 0.55 (0.52,0.58) | 85.71 (53.39,128.75) | 96.41 (61.26,141.87) | 0.21 (0.16,0.26) | 41.71 (25.22,63.64) | 52.3 (32.06,78.78) | 0.69 (0.66,0.72) |
| Myanmar | 90.91 (58.05,131.84) | 147.63 (97.96,207.69) | 1.71 (1.65,1.77) | 109.08 (68.92,160.51) | 171.75 (112.39,243.54) | 1.62 (1.55,1.68) | 63.56 (38.88,95.31) | 102.66 (66.15,147.46) | 1.67 (1.62,1.72) |
| Namibia | 40.6 (25.52,59.98) | 46.04 (29.45,66.47) | 0.44 (0.28,0.61) | 47.16 (28.8,71.19) | 51.06 (32.04,74.37) | 0.31 (0.14,0.48) | 28.45 (16.99,43.67) | 34.06 (20.98,50.88) | 0.57 (0.44,0.71) |
| Nauru | 59.47 (37.5,86.77) | 92.57 (59.98,133.38) | 1.47 (1.35,1.59) | 92.7 (57.75,136.66) | 118.43 (75.83,172.21) | 0.79 (0.67,0.91) | 42.06 (25.1,63.84) | 56.92 (34.93,85.83) | 1 (0.96,1.05) |
| Nepal | 146.49 (92.27,214.35) | 182.07 (117.89,259.53) | 0.53 (0.44,0.62) | 224.99 (140.89,334.01) | 262.68 (169.11,378.89) | 0.32 (0.23,0.42) | 68.39 (41.69,103.53) | 90.81 (57.91,131.81) | 0.85 (0.75,0.94) |
| Netherlands | 228.31 (149.97,328.06) | 384.22 (257.13,548.35) | 2.55 (1.71,3.4) | 293.14 (191.8,420.72) | 512 (339.98,733.22) | 2.69 (1.81,3.58) | 105.25 (68.3,152.73) | 205.58 (136.21,297.1) | 3.12 (2.32,3.93) |
| New Zealand | 308.78 (196.14,457.28) | 297.52 (191.32,432.12) | 0.44 (0.25,0.62) | 388.18 (246.5,576.49) | 370.28 (237.96,542.11) | 0.44 (0.25,0.63) | 165.68 (103.63,247.76) | 202.63 (129.06,296.08) | 1.12 (0.88,1.37) |
| Nicaragua | 119.08 (78.15,166.26) | 137.33 (91.56,194.51) | 0.24 (0.05,0.43) | 143.12 (93.01,202.57) | 178.15 (117.97,253.89) | 0.51 (0.29,0.74) | 78.36 (50.71,112.03) | 74.88 (49.26,106.82) | -0.16 (-0.24,-0.08) |
| Niger | 90.04 (56.17,132.46) | 110.51 (70.77,160.3) | 0.68 (0.63,0.74) | 124.74 (76.14,185.14) | 155.77 (98.74,226.99) | 0.68 (0.62,0.75) | 48.91 (29.04,75.02) | 62.28 (37.97,93.47) | 0.79 (0.74,0.85) |
| Nigeria | 84.57 (53.8,124.85) | 95.37 (61.54,137.18) | 0.46 (0.41,0.52) | 110.31 (70.03,163.09) | 132.74 (85.8,190.41) | 0.76 (0.69,0.83) | 48.66 (30.65,72.55) | 55.31 (35.51,80.13) | 0.53 (0.44,0.63) |
| Niue | 67.53 (43.44,96.41) | 75.36 (49.16,107.06) | 0.31 (0.22,0.4) | 79.26 (50.21,113.93) | 90.71 (58.4,129.7) | 0.44 (0.3,0.59) | 39.47 (24.29,59.09) | 46.29 (28.76,67.73) | 0.54 (0.5,0.57) |
| North Macedonia | 59.34 (38.58,85.66) | 89.49 (57.75,131.01) | 1.62 (1.26,1.98) | 72.1 (46.2,104.66) | 111.8 (71.53,163.05) | 1.66 (1.28,2.03) | 43.4 (27.72,63.45) | 56.04 (35.75,83.28) | 1.09 (0.83,1.36) |
| Northern Mariana Islands | 87.32 (56.37,125.28) | 121.58 (80.18,169.76) | 1.33 (1.26,1.41) | 100.22 (63.68,144.85) | 153.59 (101.01,215.05) | 1.78 (1.58,1.98) | 70.93 (45.52,102.76) | 77.57 (49.77,112.18) | 0.29 (0.16,0.41) |
| Norway | 382.27 (245.39,560.62) | 393.78 (255.13,573.64) | 0.26 (0.16,0.36) | 473.76 (304.34,695.25) | 496.37 (321.87,723.59) | 0.33 (0.25,0.42) | 221.41 (140.35,327.63) | 252.08 (161.29,368.48) | 0.56 (0.42,0.69) |
| Oman | 105.63 (68.7,151.46) | 90.22 (57.81,132.12) | -0.65 (-0.82,-0.49) | 124.61 (79.47,180.3) | 109.85 (69.96,160.46) | -0.48 (-0.66,-0.3) | 68.53 (43.92,100.19) | 59.69 (37.53,89.22) | -0.67 (-0.91,-0.43) |
| Pakistan | 45.85 (28.97,68.29) | 44.9 (28.68,65.86) | -0.07 (-0.22,0.08) | 56.92 (35.61,85.37) | 51.89 (33.25,76.35) | -0.27 (-0.42,-0.12) | 37.92 (23.26,57.68) | 38.5 (24.15,57.35) | 0.01 (-0.13,0.16) |
| Palau | 153.12 (99.9,217.59) | 149.27 (98.11,212.34) | -0.02 (-0.12,0.09) | 200.23 (129.55,285) | 214 (139.66,304.13) | 0.27 (0.21,0.34) | 89.71 (56.38,132.4) | 97.36 (62.48,141.49) | 0.37 (0.29,0.45) |
| Palestine | 64 (41.79,92.67) | 61.23 (39.65,87.54) | -0.07 (-0.23,0.08) | 79.08 (50.79,116.4) | 73.15 (47.09,104.92) | -0.23 (-0.37,-0.08) | 43.36 (27.25,64.61) | 42.1 (27.01,61.81) | -0.01 (-0.25,0.23) |
| Panama | 99.03 (65.57,139.93) | 49.5 (33,69.26) | -2.43 (-2.54,-2.32) | 130.54 (84.9,185.65) | 57.12 (37.84,81.12) | -2.89 (-3.01,-2.77) | 62.36 (40.98,88.95) | 39.44 (25.48,56.12) | -1.55 (-1.63,-1.47) |
| Papua New Guinea | 139.18 (88.85,203.01) | 202.35 (130.62,287.45) | 1.1 (1.01,1.18) | 267.81 (170.11,392.28) | 409.16 (264.23,583.83) | 1.29 (1.22,1.35) | 12.34 (7.25,19.23) | 15.6 (9.28,23.5) | 0.7 (0.6,0.8) |
| Paraguay | 91.01 (60.11,129.6) | 89.15 (59.1,126.29) | 0.2 (0.1,0.3) | 104.25 (67.65,150.48) | 107.73 (70.93,153.07) | 0.46 (0.34,0.58) | 72.4 (46.89,104.37) | 61.35 (39.27,88.22) | -0.44 (-0.49,-0.38) |
| Peru | 54.01 (35.34,77.74) | 48.58 (32.02,68.66) | -0.44 (-0.57,-0.32) | 63.89 (40.77,93.87) | 55.92 (36.18,79.76) | -0.47 (-0.61,-0.33) | 42.53 (26.68,62.5) | 39.64 (25.77,56.75) | -0.38 (-0.5,-0.27) |
| Philippines | 75.77 (49.37,110.17) | 75.84 (49.93,108.33) | 0 (-0.11,0.1) | 103.09 (66.98,149.37) | 95.94 (63.11,137.28) | -0.2 (-0.34,-0.07) | 49.34 (31.95,71.79) | 45.52 (29.63,65.69) | -0.32 (-0.39,-0.25) |
| Poland | 234.25 (152.47,339.71) | 116.79 (73.59,173) | -2.53 (-2.64,-2.42) | 285.51 (186.16,414.94) | 124.79 (78.79,184.44) | -3 (-3.12,-2.88) | 121.68 (78.36,177.28) | 92.2 (57.38,137.95) | -1.04 (-1.2,-0.89) |
| Portugal | 208.39 (142.29,289) | 157.39 (104.28,225.32) | -0.41 (-0.83,0.01) | 255.61 (172.65,356.93) | 189.57 (124.81,273.96) | -0.46 (-0.9,-0.02) | 127.13 (86,178.49) | 108.28 (70.82,155.48) | -0.05 (-0.42,0.32) |
| Puerto Rico | 76.87 (50.81,109.68) | 72.08 (46.46,104.81) | -0.16 (-0.19,-0.13) | 93.64 (61.51,134.63) | 78.69 (50.64,115.42) | -0.54 (-0.55,-0.53) | 54.14 (35.1,78.46) | 61.52 (38.55,90.22) | 0.5 (0.42,0.57) |
| Qatar | 48.66 (31.38,70.85) | 47.8 (30.34,71.42) | -0.22 (-0.34,-0.1) | 47.36 (30,70.08) | 50.64 (31.59,75.97) | 0.04 (-0.06,0.14) | 45.81 (29.12,67.58) | 43.46 (27.45,65.52) | -0.39 (-0.56,-0.23) |
| Republic of Korea | 274.38 (185.95,385.49) | 267.69 (175.53,383.94) | -0.14 (-0.24,-0.04) | 306.79 (205.5,433.07) | 305.92 (200.63,439.54) | -0.11 (-0.21,-0.01) | 198.61 (132.18,282.4) | 186.5 (120.61,268.05) | -0.09 (-0.25,0.07) |
| Republic of Moldova | 106.48 (70.97,150.9) | 54.32 (35.53,77.63) | -1.94 (-2.09,-1.79) | 124.17 (82.01,178.29) | 53.13 (34.29,76.59) | -2.6 (-2.73,-2.48) | 75.73 (49.53,107.99) | 54.35 (34.86,78.76) | -0.63 (-0.91,-0.36) |
| Romania | 99.26 (66.54,139.01) | 62.84 (40.78,90.47) | -1.43 (-1.52,-1.35) | 102.26 (67.12,146.05) | 59.99 (38.67,87.72) | -1.74 (-1.83,-1.66) | 93.98 (62.13,133.03) | 65.55 (41.4,95.4) | -1.03 (-1.13,-0.94) |
| Russian Federation | 76.38 (50.67,109.68) | 60.33 (39,87.97) | -0.8 (-1.16,-0.43) | 80.12 (53.2,115.29) | 61.11 (39.68,88.76) | -0.99 (-1.33,-0.65) | 58.69 (38.62,84.84) | 54.28 (34.27,80.29) | -0.08 (-0.55,0.4) |
| Rwanda | 62.69 (38.98,92.99) | 87.03 (56.91,124.57) | 1.4 (1.26,1.54) | 80.11 (49,120.23) | 106.41 (68.75,153.75) | 1.17 (1.06,1.29) | 37.76 (21.89,58.36) | 51 (32.14,75.02) | 1.38 (1.22,1.53) |
| Saint Kitts and Nevis | 61.39 (39.14,88.55) | 78.95 (51.75,111.6) | 0.67 (0.57,0.76) | 66.14 (41.42,97.88) | 85.51 (55.46,122.89) | 0.67 (0.54,0.79) | 50.97 (31.75,75.56) | 65.06 (41.84,94.64) | 0.73 (0.66,0.8) |
| Saint Lucia | 45.73 (29.23,65.79) | 51.02 (33.43,72.28) | 0.27 (0.16,0.39) | 50.81 (31.83,74.33) | 56.33 (36.46,80.55) | 0.26 (0.12,0.4) | 36.83 (23.01,54.38) | 42.81 (27.16,62.02) | 0.48 (0.38,0.57) |
| Saint Vincent and the Grenadines | 49.67 (31.69,72.67) | 65.03 (42.37,91.98) | 0.81 (0.76,0.85) | 41.9 (25.68,62.81) | 54.43 (34.86,78.21) | 0.87 (0.82,0.93) | 62.37 (39.34,91.46) | 76.58 (49.18,109.86) | 0.54 (0.48,0.61) |
| Samoa | 69.12 (44.52,99.87) | 90.84 (59.37,127.91) | 1.05 (0.98,1.11) | 84.2 (53.05,123.21) | 120.46 (78,170.25) | 1.32 (1.23,1.42) | 40.8 (24.74,62.12) | 51.42 (32.25,74.71) | 0.8 (0.77,0.83) |
| San Marino | 160.31 (104.51,231.35) | 185.14 (121,264.14) | 0.66 (0.53,0.79) | 197.3 (127.4,285.29) | 235.39 (152.18,336.33) | 0.77 (0.63,0.91) | 105.88 (68.89,154.35) | 126.56 (82.83,181.63) | 0.79 (0.68,0.89) |
| Sao Tome and Principe | 100.82 (64.4,146.4) | 134.22 (87.99,192.15) | 0.93 (0.9,0.97) | 95.24 (59.1,139.87) | 121.77 (78,175.85) | 0.78 (0.75,0.8) | 100.33 (62.89,149.18) | 145.91 (93.61,211.98) | 1.21 (1.16,1.26) |
| Saudi Arabia | 190.03 (124.16,267.53) | 147.53 (96.38,209.17) | -1.06 (-1.21,-0.91) | 237.95 (154.41,340.48) | 173.33 (112.89,246.17) | -1.34 (-1.47,-1.2) | 143.83 (92.58,207.56) | 127.88 (83.27,182.47) | -0.62 (-0.8,-0.43) |
| Senegal | 77.43 (49.63,113) | 97.99 (63.72,140.85) | 0.73 (0.7,0.77) | 107.26 (67.63,156.44) | 131.39 (84.27,189.93) | 0.63 (0.59,0.66) | 46.11 (27.89,70.46) | 57.71 (35.44,86.64) | 0.73 (0.69,0.77) |
| Serbia | 101.1 (66.35,143.28) | 71.28 (44.96,106.36) | -1.21 (-1.27,-1.14) | 119.83 (77.36,171.43) | 80.79 (50.82,120.98) | -1.3 (-1.36,-1.24) | 70.74 (45.73,103.08) | 55.37 (34.41,82.56) | -0.87 (-0.98,-0.75) |
| Seychelles | 56.46 (35.97,81.46) | 46.47 (30.45,66.46) | -0.49 (-0.57,-0.41) | 53.58 (33.35,79.15) | 43.67 (28.08,63.49) | -0.54 (-0.64,-0.44) | 57 (35.47,84.46) | 46.68 (29.65,68.51) | -0.49 (-0.57,-0.42) |
| Sierra Leone | 68.94 (43.38,101.78) | 79.86 (51.73,114.68) | 0.38 (0.32,0.44) | 92.1 (57.41,137.03) | 103.44 (66.24,150.19) | 0.23 (0.17,0.29) | 45.43 (27.12,69.77) | 53.22 (32.89,79) | 0.49 (0.4,0.57) |
| Singapore | 124.59 (84.19,174.51) | 108.74 (71.28,156.45) | -0.46 (-0.56,-0.37) | 137.16 (91.88,193.78) | 122.46 (78.37,176.13) | -0.49 (-0.62,-0.37) | 106.05 (70.89,150.43) | 91.46 (60.14,132.42) | -0.29 (-0.37,-0.22) |
| Slovakia | 258.75 (170.95,367.19) | 144.7 (92.19,209.88) | -1.6 (-1.92,-1.27) | 311.72 (204.78,443.56) | 156.34 (99.42,226.57) | -1.88 (-2.3,-1.46) | 159.23 (103.53,230.21) | 113.19 (70.63,168.73) | -1.08 (-1.18,-0.98) |
| Slovenia | 324.72 (209.8,458.04) | 263.41 (169.45,382.78) | -0.03 (-0.28,0.22) | 392.86 (253.18,555.2) | 297.89 (191.18,431.24) | -0.24 (-0.52,0.04) | 188.86 (120.65,274.04) | 190.17 (120.25,281.04) | 0.58 (0.36,0.8) |
| Solomon Islands | 160.44 (100.33,236.52) | 233.28 (150.31,336.33) | 1.27 (1.23,1.31) | 328.15 (204.46,486.12) | 417.09 (267.29,602.25) | 0.77 (0.74,0.8) | 32.52 (19.08,50.25) | 49.37 (29.86,74.58) | 1.38 (1.34,1.41) |
| Somalia | 45.53 (27.93,68.47) | 54.85 (34.73,81.73) | 0.65 (0.62,0.68) | 58.66 (35.04,90.15) | 66.75 (41.42,99.84) | 0.44 (0.41,0.48) | 29.4 (17.38,45.64) | 32.11 (19.22,49.85) | 0.34 (0.31,0.38) |
| South Africa | 39.62 (25.91,57.34) | 26.39 (17.14,38.09) | -1.74 (-1.84,-1.63) | 45.27 (29.56,65.4) | 29 (18.81,41.93) | -1.9 (-2.03,-1.78) | 29.43 (19.11,43.02) | 20.68 (13.24,30.32) | -1.44 (-1.52,-1.37) |
| South Sudan | 47.86 (30.29,70.83) | 51.22 (32.48,74.47) | 0.16 (0.13,0.19) | 65.07 (40.37,97.07) | 71.17 (45.06,104.41) | 0.22 (0.18,0.26) | 31.67 (18.65,49.25) | 33.5 (20.22,50.96) | 0.18 (0.09,0.27) |
| Spain | 131.35 (86.82,185.19) | 181.17 (119.11,257.86) | 1.48 (1.29,1.66) | 161.11 (105.73,228.4) | 232.13 (153.13,331.14) | 1.66 (1.46,1.85) | 81.79 (53.82,116.12) | 107.01 (69.48,154.89) | 1.21 (1.05,1.37) |
| Sri Lanka | 94.22 (62.03,134.12) | 130.14 (86.14,183.75) | 0.81 (0.61,1.01) | 122.09 (79.38,174.4) | 156.06 (102.81,221.29) | 0.55 (0.36,0.74) | 66.82 (42.72,98.47) | 83.75 (54.19,121.72) | 0.5 (0.31,0.69) |
| Sudan | 45.45 (29.11,66.47) | 48.96 (32.22,69.58) | 0.14 (0.06,0.21) | 57.21 (35.98,85.48) | 64.15 (41.28,92.28) | 0.3 (0.22,0.39) | 33.5 (20.48,50.26) | 36.56 (23.26,53.55) | 0.19 (0.11,0.27) |
| Suriname | 64.44 (41.31,93.39) | 77.3 (50.63,109.1) | 0.49 (0.42,0.56) | 83.78 (52.99,122.95) | 97.32 (62.79,137.87) | 0.36 (0.28,0.43) | 39.55 (24.43,59.16) | 47.12 (30.29,68.08) | 0.43 (0.34,0.51) |
| Sweden | 278.44 (175.32,414.31) | 302.71 (194.83,441.2) | 0.4 (0.25,0.55) | 341.7 (214.7,509.27) | 366.37 (236.76,536.58) | 0.36 (0.23,0.5) | 170.48 (105.91,257.2) | 217.91 (139.08,319.76) | 0.91 (0.73,1.09) |
| Switzerland | 465.86 (307.22,662.75) | 404.2 (269.51,572.47) | -0.29 (-0.45,-0.14) | 584.56 (385.05,833.87) | 514.81 (341.81,729.07) | -0.24 (-0.42,-0.06) | 244.18 (157.89,350.47) | 248.72 (164.4,355.98) | 0.2 (0.11,0.29) |
| Syrian Arab Republic | 46.1 (30.15,66.37) | 42.64 (27.86,61.27) | -0.73 (-0.97,-0.5) | 69.28 (43.93,101.53) | 66.31 (42.97,95.71) | -0.68 (-0.95,-0.41) | 25.92 (15.96,38.85) | 26.39 (16.71,38.77) | -0.46 (-0.73,-0.18) |
| Taiwan (Province of China) | 78.76 (52,111.29) | 43.07 (27.3,63.12) | -2.12 (-2.41,-1.84) | 94.71 (62.19,134.97) | 46.19 (29.07,67.47) | -2.47 (-2.74,-2.19) | 60.84 (39.57,87.58) | 38.68 (24.22,57.59) | -1.7 (-1.97,-1.44) |
| Tajikistan | 44.26 (28.84,63.81) | 34.54 (21.82,50.59) | -1.05 (-1.17,-0.94) | 46 (29.11,67.81) | 37.34 (22.54,56.33) | -0.85 (-0.95,-0.76) | 40.69 (25.43,60.11) | 31.35 (19.43,47.03) | -1.11 (-1.25,-0.98) |
| Thailand | 83.32 (54.84,118.63) | 67.43 (44.36,95.81) | -0.84 (-1.01,-0.66) | 105.22 (68.25,150.51) | 80.44 (52.3,114.65) | -1.06 (-1.28,-0.85) | 49.07 (31.48,72.04) | 48.64 (31.88,70.56) | -0.07 (-0.16,0.03) |
| Timor-Leste | 54.7 (35.23,79.98) | 80.58 (52.28,115.63) | 1.53 (1.44,1.61) | 70.82 (44.79,105.16) | 105.63 (68.12,151.36) | 1.53 (1.42,1.64) | 37 (22.63,56.4) | 54.07 (34.15,80.49) | 1.49 (1.4,1.57) |
| Togo | 80.93 (51.63,118.24) | 92.49 (60.27,133.51) | 0.27 (0.18,0.37) | 104.68 (65.43,154.72) | 110.01 (71.15,158.76) | -0.06 (-0.16,0.05) | 51.84 (31.99,78.25) | 58.46 (36.43,86.95) | 0.27 (0.19,0.35) |
| Tokelau | 58.23 (37.48,83.88) | 73.68 (48.15,104.87) | 0.83 (0.79,0.88) | 79.64 (50.39,116.29) | 104.19 (67.47,148.54) | 1 (0.92,1.09) | 31.94 (19.08,48.58) | 38.29 (23.91,56.64) | 0.63 (0.57,0.69) |
| Tonga | 61.74 (39.69,88.55) | 66 (43.33,92.91) | 0.15 (0.07,0.23) | 94.57 (60.19,136.27) | 98.42 (64.12,139.21) | 0.05 (0,0.1) | 15.95 (9.38,24.57) | 17.78 (10.49,27.27) | 0.19 (0.1,0.28) |
| Trinidad and Tobago | 62.76 (40.63,90.19) | 42.52 (27.91,60.09) | -1.35 (-1.42,-1.28) | 77.29 (49.28,112.54) | 49.53 (32.12,71.38) | -1.57 (-1.64,-1.49) | 38.43 (23.77,57.03) | 31.52 (19.78,46.02) | -0.63 (-0.68,-0.57) |
| Tunisia | 51.74 (33.85,72.92) | 47.15 (30.99,66.54) | -0.3 (-0.4,-0.19) | 64.28 (41.03,92.46) | 57.33 (37.57,81.33) | -0.37 (-0.48,-0.26) | 37.24 (23.6,53.77) | 33.46 (21.33,48.35) | -0.4 (-0.51,-0.29) |
| Turkey | 58.34 (37.44,83.78) | 88.01 (56.91,124.89) | 1.82 (1.52,2.12) | 75.14 (47.54,109.04) | 117.09 (75.46,166.06) | 1.98 (1.72,2.24) | 32.05 (19.73,48.32) | 46.49 (29.71,68.14) | 1.61 (1.39,1.83) |
| Turkmenistan | 32.89 (21.31,48.28) | 24.42 (15.2,35.44) | -0.8 (-0.97,-0.64) | 37.5 (23.39,56.48) | 27.95 (16.8,41.43) | -0.75 (-0.88,-0.61) | 23.88 (14.3,35.69) | 18.89 (11.37,28.62) | -0.59 (-0.8,-0.38) |
| Tuvalu | 61.14 (38.49,89.93) | 80.83 (52.39,114.93) | 0.97 (0.87,1.07) | 73.78 (45.76,109.46) | 103.64 (66.33,148.36) | 1.25 (1.12,1.37) | 34.75 (20.55,54.09) | 46.64 (28.79,69.05) | 1.01 (0.91,1.12) |
| Uganda | 59.35 (37.4,87.35) | 84.62 (54.58,121.46) | 1.35 (1.26,1.45) | 76.97 (47.88,114.22) | 104.08 (66.55,151.03) | 1.19 (1.09,1.3) | 36.93 (22.12,57) | 49.04 (30.38,72.49) | 1.1 (1,1.21) |
| Ukraine | 89.03 (58.67,130.58) | 59.72 (38.78,86.41) | -1.68 (-1.98,-1.38) | 90.84 (59.17,134.18) | 54.55 (34.86,79.85) | -2.08 (-2.36,-1.79) | 79.29 (50.87,116.72) | 65 (41.66,94.92) | -0.96 (-1.31,-0.61) |
| United Arab Emirates | 57.84 (38.04,82.58) | 34.68 (22.82,49.03) | -2.09 (-2.53,-1.64) | 71.1 (46.08,102.33) | 46.03 (29.81,65.14) | -1.7 (-2.23,-1.16) | 43.76 (28.44,64.35) | 33.87 (21.87,48.3) | -1.2 (-1.36,-1.03) |
| United Kingdom | 196.78 (129.34,282.64) | 227.04 (146.61,327.67) | 0.82 (0.69,0.96) | 252.33 (165.88,363.56) | 298.06 (192.5,431.1) | 0.9 (0.77,1.04) | 93.46 (60.63,135.14) | 135.31 (87.22,195.88) | 1.58 (1.42,1.75) |
| United Republic of Tanzania | 68.16 (42.56,99.78) | 85.87 (55.63,123.67) | 0.92 (0.8,1.04) | 91.14 (56.22,134.02) | 113.52 (72.82,164.71) | 0.85 (0.71,0.99) | 40.15 (24,61.75) | 49.96 (31.01,75.07) | 0.91 (0.81,1.02) |
| United States of America | 177.39 (113.79,260.76) | 266.76 (169.58,388.97) | 1.54 (1.38,1.69) | 215.18 (137.92,317.48) | 335.19 (212.72,489.1) | 1.66 (1.51,1.81) | 112.97 (71.86,166.42) | 178.84 (113.8,261.51) | 1.76 (1.6,1.92) |
| United States Virgin Islands | 68.31 (43.9,97.05) | 71.04 (46.21,102.04) | 0.16 (0.12,0.19) | 75.72 (48.26,108.52) | 75.39 (48.49,107.84) | -0.03 (-0.1,0.05) | 53.85 (33.94,78.62) | 60.4 (38.46,88.38) | 0.39 (0.35,0.44) |
| Uruguay | 232.37 (158.21,322.67) | 190.27 (129.74,263.83) | -0.56 (-0.68,-0.43) | 297.95 (201.79,415.68) | 243.26 (165.08,338.25) | -0.56 (-0.69,-0.42) | 123.35 (82.63,175.77) | 96.05 (64.13,134.7) | -0.76 (-0.87,-0.66) |
| Uzbekistan | 36.45 (23.69,52.41) | 29.15 (18.76,42.26) | -0.63 (-0.75,-0.51) | 39.9 (25.19,58.99) | 31.52 (19.48,46.93) | -0.66 (-0.77,-0.55) | 29.8 (18.78,43.91) | 25.12 (15.39,37.42) | -0.44 (-0.59,-0.29) |
| Vanuatu | 44.5 (28.25,65.21) | 60.77 (38.94,87.67) | 1.04 (0.97,1.12) | 63.59 (39.69,93.9) | 80.48 (50.71,116.5) | 0.79 (0.69,0.88) | 27.44 (16.13,42.46) | 37.25 (22.45,57.33) | 1.03 (1.01,1.05) |
| Venezuela (Bolivarian Republic of) | 125.25 (82.37,175.81) | 82.42 (54.1,115.84) | -1.5 (-1.58,-1.42) | 169.98 (110.22,240.41) | 108.68 (71.24,154.14) | -1.6 (-1.7,-1.5) | 68.41 (44.3,98.36) | 43.19 (27.77,62.93) | -1.55 (-1.64,-1.47) |
| Viet Nam | 144.05 (92.55,209.91) | 181.64 (119.31,257.84) | 0.77 (0.6,0.94) | 182.67 (116.27,266.86) | 225.76 (147.94,321.36) | 0.7 (0.51,0.89) | 63.01 (38.62,95.11) | 83.72 (52.93,122.46) | 0.93 (0.84,1.03) |
| Yemen | 46.59 (29.63,68.56) | 49.18 (32.02,71.01) | 0.09 (-0.03,0.2) | 53.45 (33.48,80.42) | 60.6 (38.33,88.54) | 0.33 (0.19,0.46) | 32.34 (19.9,49.13) | 36.41 (23.25,53.18) | 0.37 (0.24,0.49) |
| Zambia | 70.58 (44.63,103.12) | 88.03 (56.84,128.37) | 0.95 (0.79,1.11) | 98.37 (61.49,146.6) | 112.14 (71.19,165.18) | 0.57 (0.43,0.7) | 46.05 (27.28,70.38) | 54.97 (34.09,81.63) | 0.86 (0.71,1.02) |
| Zimbabwe | 70.59 (45.02,103.08) | 63.58 (40.28,92.51) | -0.33 (-0.61,-0.06) | 86.36 (54.15,128.23) | 80.16 (49.64,119.01) | -0.16 (-0.47,0.16) | 41.32 (25.44,61.64) | 37.29 (22.69,56.14) | -0.28 (-0.44,-0.12) |

| **Table S6. Age-Period-Cohort analysis of incidence of hip fractures attributable to falls in older adults.** | | | | | | |
| --- | --- | --- | --- | --- | --- | --- |
|  | **Both** |  | **Female** |  | **Male** |  |
| **Annal change of prevalence** | | | | | | |
|  | **Per year (%)** | **P** | **Per year (%)** | **P** | **Per year (%)** | **P** |
| 55-59 years | 0.325(0.214,0.435) | < 0.001 | 0.392(0.269,0.516) | < 0.001 | 0.194(0.035,0.354) | < 0.001 |
| 60-64 years | 0.431(0.35,0.511) | < 0.001 | 0.453(0.366,0.541) | < 0.001 | 0.381(0.26,0.502) | < 0.001 |
| 65-69 years | 0.381(0.315,0.447) | < 0.001 | 0.358(0.289,0.427) | < 0.001 | 0.479(0.375,0.584) | < 0.001 |
| 70-74 years | 0.21(0.153,0.268) | < 0.001 | 0.16(0.102,0.218) | < 0.001 | 0.515(0.419,0.612) | < 0.001 |
| 75-79 years | 0.16(0.107,0.213) | < 0.001 | 0.085(0.033,0.137) | < 0.001 | 0.666(0.569,0.762) | < 0.001 |
| 80-84 years | 0.061(0.011,0.111) | < 0.001 | -0.033(-0.081,0.015) | < 0.001 | 0.805(0.703,0.906) | < 0.001 |
| 85-89 years | 0.159(0.101,0.216) | < 0.001 | 0.049(-0.004,0.102) | < 0.001 | 1.123(0.992,1.254) | < 0.001 |
| 90-94 years | 0.55(0.462,0.637) | < 0.001 | 0.436(0.357,0.514) | < 0.001 | 1.632(1.408,1.857) | < 0.001 |
| 95+ years | 1.089(0.909,1.27) | < 0.001 | 1.016(0.857,1.176) | < 0.001 | 2.127(1.614,2.641) | < 0.001 |
| **Age** | | | | | | |
|  | **Rate (95% CI)** | **P** | **Rate (95% CI)** | **P** | **Rate (95% CI)** | **P** |
| 55-59 years | 137.171(134.655,139.733) | < 0.001 | 142.786(139.861,145.772) | < 0.001 | 132.408(128.915,135.995) | < 0.001 |
| 60-64 years | 220.865(217.471,224.311) | < 0.001 | 252.567(248.406,256.797) | < 0.001 | 188.602(184.224,193.084) | < 0.001 |
| 65-69 years | 370.01(365.035,375.052) | < 0.001 | 447.528(441.26,453.885) | < 0.001 | 287.052(280.966,293.271) | < 0.001 |
| 70-74 years | 681.342(673.122,689.661) | < 0.001 | 839.921(829.527,850.444) | < 0.001 | 501.966(492.148,511.981) | < 0.001 |
| 75-79 years | 1365.792(1350.516,1381.24) | < 0.001 | 1720.788(1701.277,1740.523) | < 0.001 | 924.107(906.89,941.652) | < 0.001 |
| 80-84 years | 2815.601(2778.958,2852.727) | < 0.001 | 3503.174(3457.175,3549.786) | < 0.001 | 1837.227(1796.726,1878.641) | < 0.001 |
| 85-89 years | 5324.608(5251.714,5398.514) | < 0.001 | 6389.335(6301.913,6477.969) | < 0.001 | 3499.455(3415.595,3585.373) | < 0.001 |
| 90-94 years | 8666.27(8533.242,8801.372) | < 0.001 | 10069.932(9918.544,10223.63) | < 0.001 | 5605.38(5441.73,5773.951) | < 0.001 |
| 95+ years | 12614.641(12369.269,12864.88) | < 0.001 | 14490.297(14223.757,14761.833) | < 0.001 | 7661.276(7332.56,8004.728) | < 0.001 |
| **Period** | | | | | | |
|  | **Rate ratio (95% CI)** | **P** | **Rate ratio (95% CI)** | **P** | **Rate ratio (95% CI)** | **P** |
| 1992-1996 | 1(1,1) | < 0.001 | 1(1,1) | < 0.001 | 1(1,1) | < 0.001 |
| 1997-2001 | 1.032(1.02,1.045) | < 0.001 | 1.024(1.012,1.035) | < 0.001 | 1.063(1.039,1.086) | < 0.001 |
| 2002-2006 | 1.031(1.019,1.044) | < 0.001 | 1.02(1.008,1.031) | < 0.001 | 1.085(1.062,1.109) | < 0.001 |
| 2007-2011 | 1.048(1.036,1.06) | < 0.001 | 1.035(1.024,1.047) | < 0.001 | 1.126(1.102,1.151) | < 0.001 |
| 2012-2016 | 1.068(1.056,1.08) | < 0.001 | 1.051(1.04,1.063) | < 0.001 | 1.183(1.158,1.209) | < 0.001 |
| 2017-2021 | 1.097(1.085,1.109) | < 0.001 | 1.081(1.069,1.092) | < 0.001 | 1.247(1.22,1.275) | < 0.001 |
| **Cohort** | | | | | | |
|  | **Rate ratio (95% CI)** | **P** | **Rate ratio (95% CI)** | **P** | **Rate ratio (95% CI)** | **P** |
| 1893—1901 | 0.736(0.693,0.781) | < 0.001 | 0.754(0.715,0.796) | < 0.001 | 0.534(0.451,0.633) | < 0.001 |
| 1898—1906 | 0.823(0.799,0.848) | < 0.001 | 0.852(0.829,0.875) | < 0.001 | 0.61(0.567,0.657) | < 0.001 |
| 1903—1911 | 0.91(0.892,0.929) | < 0.001 | 0.942(0.925,0.96) | < 0.001 | 0.705(0.675,0.736) | < 0.001 |
| 1908—1916 | 0.957(0.941,0.973) | < 0.001 | 0.981(0.965,0.997) | < 0.001 | 0.793(0.767,0.819) | < 0.001 |
| 1913—1921 | 0.94(0.926,0.955) | < 0.001 | 0.957(0.943,0.972) | < 0.001 | 0.833(0.81,0.856) | < 0.001 |
| 1918—1926 | 0.982(0.969,0.996) | < 0.001 | 0.994(0.98,1.008) | < 0.001 | 0.904(0.882,0.927) | < 0.001 |
| 1923—1931 | 0.956(0.943,0.969) | < 0.001 | 0.965(0.952,0.978) | < 0.001 | 0.917(0.896,0.938) | < 0.001 |
| 1928—1936 | 0.954(0.942,0.967) | < 0.001 | 0.96(0.948,0.973) | < 0.001 | 0.94(0.92,0.96) | < 0.001 |
| 1933—1941 | 0.974(0.962,0.987) | < 0.001 | 0.973(0.961,0.986) | < 0.001 | 0.973(0.953,0.994) | < 0.001 |
| 1938—1946 | 1(1,1) | < 0.001 | 1(1,1) | < 0.001 | 1(1,1) | < 0.001 |
| 1943—1951 | 1.025(1.009,1.041) | < 0.001 | 1.026(1.01,1.043) | < 0.001 | 1.021(0.996,1.046) | < 0.001 |
| 1948—1956 | 1.041(1.022,1.06) | < 0.001 | 1.045(1.025,1.066) | < 0.001 | 1.026(0.998,1.055) | < 0.001 |
| 1953—1961 | 1.061(1.037,1.085) | < 0.001 | 1.072(1.046,1.099) | < 0.001 | 1.036(1.001,1.072) | < 0.001 |
| 1958—1966 | 1.05(1.016,1.085) | < 0.001 | 1.067(1.028,1.107) | < 0.001 | 1.019(0.972,1.068) | < 0.001 |

| **Table S7. Age-Period-Cohort analysis of prevalence of hip fractures attributable to falls in older adults.** | | | | | | |
| --- | --- | --- | --- | --- | --- | --- |
|  | **Both** |  | **Female** |  | **Male** |  |
| **Annal change of prevalence** | | | | | | |
|  | **Per year (%)** |  | **Per year (%)** |  | **Per year (%)** |  |
| 55-59 years | 0.099(-0.077,0.275) | < 0.001 | 0.184(0.049,0.319) | < 0.001 | -0.091(-0.339,0.158) | < 0.001 |
| 60-64 years | 0.361(0.238,0.484) | < 0.001 | 0.401(0.311,0.492) | < 0.001 | 0.271(0.09,0.452) | < 0.001 |
| 65-69 years | 0.47(0.372,0.569) | < 0.001 | 0.462(0.392,0.533) | < 0.001 | 0.568(0.413,0.723) | < 0.001 |
| 70-74 years | 0.42(0.334,0.506) | < 0.001 | 0.383(0.323,0.443) | < 0.001 | 0.783(0.637,0.93) | < 0.001 |
| 75-79 years | 0.38(0.3,0.46) | < 0.001 | 0.312(0.258,0.366) | < 0.001 | 1(0.851,1.15) | < 0.001 |
| 80-84 years | 0.284(0.206,0.362) | < 0.001 | 0.205(0.155,0.256) | < 0.001 | 1.169(1.006,1.333) | < 0.001 |
| 85-89 years | 0.431(0.339,0.524) | < 0.001 | 0.345(0.287,0.403) | < 0.001 | 1.562(1.341,1.784) | < 0.001 |
| 90-94 years | 0.837(0.689,0.986) | < 0.001 | 0.737(0.646,0.829) | < 0.001 | 2.129(1.732,2.528) | < 0.001 |
| 95+ years | 1.347(1.021,1.674) | < 0.001 | 1.274(1.075,1.473) | < 0.001 | 2.598(1.647,3.557) | < 0.001 |
| **Age** | | | | | | |
|  | **Rate (95% CI)** | **P** | **Rate (95% CI)** | **P** | **Rate (95% CI)** | **P** |
| 55-59 years | 254.518(247.271,261.978) | < 0.001 | 272.951(266.973,279.062) | < 0.001 | 238.478(228.927,248.429) | < 0.001 |
| 60-64 years | 472.647(461.862,483.683) | < 0.001 | 552.042(542.754,561.489) | < 0.001 | 392.552(379.246,406.325) | < 0.001 |
| 65-69 years | 867.662(850.769,884.89) | < 0.001 | 1085.504(1070.439,1100.781) | < 0.001 | 634.934(615.561,654.917) | < 0.001 |
| 70-74 years | 1472.402(1446.209,1499.069) | < 0.001 | 1916.383(1892.562,1940.504) | < 0.001 | 968.33(940.229,997.27) | < 0.001 |
| 75-79 years | 2910.018(2861.675,2959.177) | < 0.001 | 3805.865(3762.03,3850.211) | < 0.001 | 1797.468(1747.718,1848.634) | < 0.001 |
| 80-84 years | 5932.702(5817.731,6049.946) | < 0.001 | 7646.201(7543.971,7749.817) | < 0.001 | 3501.283(3385.904,3620.593) | < 0.001 |
| 85-89 years | 10837.07(10614.646,11064.155) | < 0.001 | 13505.392(13316.119,13697.356) | < 0.001 | 6255.374(6027.453,6491.914) | < 0.001 |
| 90-94 years | 16061.251(15681.865,16449.815) | < 0.001 | 19226.301(18922.716,19534.757) | < 0.001 | 9197.111(8771.743,9643.107) | < 0.001 |
| 95+ years | 19878.994(19238.931,20540.352) | < 0.001 | 23218.124(22734.916,23711.601) | < 0.001 | 11344.146(10517.146,12236.175) | < 0.001 |
| **Period** | | | | | | |
|  | **Rate ratio (95% CI)** | **P** | **Rate ratio (95% CI)** | **P** | **Rate ratio (95% CI)** | **P** |
| 1992-1996 | 1(1,1) | < 0.001 | 1(1,1) | < 0.001 | 1(1,1) | < 0.001 |
| 1997-2001 | 1.037(1.018,1.056) | < 0.001 | 1.031(1.018,1.043) | < 0.001 | 1.066(1.03,1.103) | < 0.001 |
| 2002-2006 | 1.05(1.031,1.069) | < 0.001 | 1.041(1.029,1.054) | < 0.001 | 1.106(1.07,1.145) | < 0.001 |
| 2007-2011 | 1.084(1.065,1.104) | < 0.001 | 1.075(1.062,1.088) | < 0.001 | 1.173(1.134,1.213) | < 0.001 |
| 2012-2016 | 1.11(1.091,1.13) | < 0.001 | 1.098(1.085,1.111) | < 0.001 | 1.245(1.203,1.289) | < 0.001 |
| 2017-2021 | 1.129(1.11,1.148) | < 0.001 | 1.116(1.104,1.129) | < 0.001 | 1.313(1.266,1.361) | < 0.001 |
| **Cohort** | | | | | | |
|  | **Rate ratio (95% CI)** | **P** | **Rate ratio (95% CI)** | **P** | **Rate ratio (95% CI)** | **P** |
| 1893—1901 | 0.662(0.594,0.738) | < 0.001 | 0.678(0.635,0.725) | < 0.001 | 0.45(0.329,0.616) | < 0.001 |
| 1898—1906 | 0.746(0.709,0.784) | < 0.001 | 0.77(0.746,0.794) | < 0.001 | 0.518(0.455,0.59) | < 0.001 |
| 1903—1911 | 0.83(0.805,0.857) | < 0.001 | 0.856(0.839,0.874) | < 0.001 | 0.613(0.571,0.659) | < 0.001 |
| 1908—1916 | 0.89(0.868,0.914) | < 0.001 | 0.909(0.894,0.925) | < 0.001 | 0.714(0.678,0.752) | < 0.001 |
| 1913—1921 | 0.885(0.865,0.906) | < 0.001 | 0.9(0.886,0.914) | < 0.001 | 0.764(0.731,0.798) | < 0.001 |
| 1918—1926 | 0.941(0.921,0.961) | < 0.001 | 0.95(0.937,0.964) | < 0.001 | 0.849(0.818,0.882) | < 0.001 |
| 1923—1931 | 0.927(0.909,0.946) | < 0.001 | 0.937(0.924,0.95) | < 0.001 | 0.879(0.85,0.91) | < 0.001 |
| 1928—1936 | 0.931(0.913,0.949) | < 0.001 | 0.938(0.926,0.951) | < 0.001 | 0.912(0.883,0.941) | < 0.001 |
| 1933—1941 | 0.957(0.939,0.975) | < 0.001 | 0.955(0.943,0.968) | < 0.001 | 0.957(0.928,0.988) | < 0.001 |
| 1938—1946 | 1(1,1) | < 0.001 | 1(1,1) | < 0.001 | 1(1,1) | < 0.001 |
| 1943—1951 | 1.035(1.012,1.059) | < 0.001 | 1.041(1.024,1.058) | < 0.001 | 1.023(0.987,1.06) | < 0.001 |
| 1948—1956 | 1.016(0.989,1.044) | < 0.001 | 1.025(1.005,1.045) | < 0.001 | 0.992(0.952,1.033) | < 0.001 |
| 1953—1961 | 1.012(0.976,1.048) | < 0.001 | 1.026(1,1.054) | < 0.001 | 0.977(0.928,1.028) | < 0.001 |
| 1958—1966 | 0.988(0.936,1.042) | < 0.001 | 1.006(0.965,1.048) | < 0.001 | 0.947(0.878,1.02) | < 0.001 |

| **Table S8. Age-Period-Cohort analysis of YLDs of hip fractures attributable to falls in older adults.** | | | | | | |
| --- | --- | --- | --- | --- | --- | --- |
|  | **Both** |  | **Female** |  | **Male** |  |
| **Annal change of prevalence** | | | | | | |
|  | **Per year (%)** | **P** | **Per year (%)** | **P** | **Per year (%)** | **P** |
| 55-59 years | -0.994(-1.148, -0.84) | < 0.001 | -0.926(-1.053, -0.799) | < 0.001 | -1.145(-1.342, -0.947) | < 0.001 |
| 60-64 years | -0.738(-0.845, -0.63) | < 0.001 | -0.697(-0.782, -0.611) | < 0.001 | -0.823(-0.967, -0.678) | < 0.001 |
| 65-69 years | -0.502(-0.588, -0.415) | < 0.001 | -0.472(-0.538, -0.405) | < 0.001 | -0.49(-0.614, -0.366) | < 0.001 |
| 70-74 years | -0.343(-0.42, -0.266) | < 0.001 | -0.309(-0.366, -0.252) | < 0.001 | -0.172(-0.29, -0.053) | < 0.001 |
| 75-79 years | -0.211(-0.284, -0.139) | < 0.001 | -0.192(-0.245, -0.14) | < 0.001 | 0.142(0.019,0.265) | < 0.001 |
| 80-84 years | -0.164(-0.238, -0.091) | < 0.001 | -0.168(-0.219, -0.116) | < 0.001 | 0.424(0.286,0.562) | < 0.001 |
| 85-89 years | 0.021(-0.069,0.111) | < 0.001 | -0.018(-0.079,0.044) | < 0.001 | 0.861(0.673,1.05) | < 0.001 |
| 90-94 years | 0.458(0.312,0.604) | < 0.001 | 0.384(0.286,0.481) | < 0.001 | 1.462(1.123,1.802) | < 0.001 |
| 95+ years | 1.038(0.719,1.359) | < 0.001 | 0.975(0.765,1.186) | < 0.001 | 2.046(1.237,2.862) | < 0.001 |
| **Age** | | | | | | |
|  | **Rate (95% CI)** | **P** | **Rate (95% CI)** | **P** | **Rate (95% CI)** | **P** |
| 55-59 years | 32.417(31.625,33.23) | < 0.001 | 34.676(33.977,35.388) | < 0.001 | 30.381(29.421,31.371) | < 0.001 |
| 60-64 years | 55.422(54.314,56.553) | < 0.001 | 65.411(64.381,66.457) | < 0.001 | 45.232(43.993,46.506) | < 0.001 |
| 65-69 years | 93.734(92.098,95.399) | < 0.001 | 118.838(117.259,120.438) | < 0.001 | 66.82(65.134,68.549) | < 0.001 |
| 70-74 years | 147.799(145.401,150.236) | < 0.001 | 194.172(191.816,196.556) | < 0.001 | 95.18(92.864,97.555) | < 0.001 |
| 75-79 years | 268.493(264.378,272.673) | < 0.001 | 354.585(350.554,358.662) | < 0.001 | 162.061(158.248,165.967) | < 0.001 |
| 80-84 years | 495.109(486.284,504.094) | < 0.001 | 644.078(635.598,652.671) | < 0.001 | 286.27(278.292,294.476) | < 0.001 |
| 85-89 years | 817.794(802.162,833.73) | < 0.001 | 1026.648(1012.311,1041.188) | < 0.001 | 468.637(454.014,483.731) | < 0.001 |
| 90-94 years | 1149.006(1123.141,1175.466) | < 0.001 | 1385.761(1363.478,1408.409) | < 0.001 | 659.928(633.232,687.751) | < 0.001 |
| 95+ years | 1420.624(1375.933,1466.766) | < 0.001 | 1675.43(1638.981,1712.69) | < 0.001 | 810.379(758.003,866.374) | < 0.001 |
| **Period** | | | | | | |
|  | **Rate ratio (95% CI)** | **P** | **Rate ratio (95% CI)** | **P** | **Rate ratio (95% CI)** | **P** |
| 1992-1996 | 1(1,1) |  | 1(1,1) |  | 1(1,1) |  |
| 1997-2001 | 0.995(0.979,1.012) | < 0.001 | 0.992(0.98,1.004) | < 0.001 | 1.016(0.989,1.044) | < 0.001 |
| 2002-2006 | 0.967(0.952,0.983) | < 0.001 | 0.966(0.955,0.978) | < 0.001 | 0.998(0.971,1.026) | < 0.001 |
| 2007-2011 | 0.959(0.943,0.974) | < 0.001 | 0.96(0.949,0.972) | < 0.001 | 1.005(0.977,1.033) | < 0.001 |
| 2012-2016 | 0.959(0.943,0.974) | < 0.001 | 0.958(0.947,0.969) | < 0.001 | 1.034(1.005,1.064) | < 0.001 |
| 2017-2021 | 0.963(0.948,0.978) | < 0.001 | 0.962(0.951,0.973) | < 0.001 | 1.07(1.039,1.103) | < 0.001 |
| **Cohort** | | | | | | |
|  | **Rate ratio (95% CI)** | **P** | **Rate ratio (95% CI)** | **P** | **Rate ratio (95% CI)** | **P** |
| 1893—1901 | 0.812(0.73,0.903) | < 0.001 | 0.815(0.76,0.874) | < 0.001 | 0.61(0.467,0.798) | < 0.001 |
| 1898—1906 | 0.909(0.865,0.954) | < 0.001 | 0.918(0.889,0.949) | < 0.001 | 0.701(0.627,0.783) | < 0.001 |
| 1903—1911 | 1.002(0.972,1.033) | < 0.001 | 1.012(0.991,1.033) | < 0.001 | 0.81(0.762,0.862) | < 0.001 |
| 1908—1916 | 1.049(1.024,1.075) | < 0.001 | 1.05(1.032,1.068) | < 0.001 | 0.905(0.866,0.946) | < 0.001 |
| 1913—1921 | 1.043(1.02,1.065) | < 0.001 | 1.039(1.023,1.056) | < 0.001 | 0.954(0.92,0.989) | < 0.001 |
| 1918—1926 | 1.063(1.043,1.084) | < 0.001 | 1.055(1.041,1.071) | < 0.001 | 1.008(0.977,1.04) | < 0.001 |
| 1923—1931 | 1.03(1.012,1.049) | < 0.001 | 1.026(1.012,1.04) | < 0.001 | 1.011(0.983,1.04) | < 0.001 |
| 1928—1936 | 1.017(0.999,1.035) | < 0.001 | 1.016(1.003,1.03) | < 0.001 | 1.012(0.985,1.04) | < 0.001 |
| 1933—1941 | 1.012(0.994,1.03) | < 0.001 | 1.009(0.996,1.022) | < 0.001 | 1.012(0.986,1.039) | < 0.001 |
| 1938—1946 | 1(1,1) | < 0.001 | 1(1,1) | < 0.001 | 1(1,1) | < 0.001 |
| 1943—1951 | 0.961(0.941,0.981) | < 0.001 | 0.963(0.948,0.978) | < 0.001 | 0.956(0.928,0.984) | < 0.001 |
| 1948—1956 | 0.896(0.875,0.918) | < 0.001 | 0.899(0.883,0.917) | < 0.001 | 0.883(0.854,0.914) | < 0.001 |
| 1953—1961 | 0.851(0.825,0.878) | < 0.001 | 0.859(0.837,0.881) | < 0.001 | 0.83(0.796,0.866) | < 0.001 |
| 1958—1966 | 0.797(0.76,0.836) | < 0.001 | 0.809(0.778,0.842) | < 0.001 | 0.768(0.723,0.817) | < 0.001 |

| **Table S9. Decomposition analysis of hip fractures attributable to falls in older adults from 1990 to 2021.** | | | | | | | | |
| --- | --- | --- | --- | --- | --- | --- | --- | --- |
|  | **Sex name** | **Over all difference** | **Aging effect** | **Population growth effect** | **Epidemiological changes effect** | **Aging percent** | **Population growth percent** | **Epidemiological changes percent** |
| Incidence | Both | 8209555.97 | 1335309.799 | 6159109.565 | 715136.61 | 16.27 | 75.02 | 8.71 |
|  | Male | 2584737.48 | 354624.645 | 1697791.183 | 532321.65 | 13.72 | 65.69 | 20.59 |
|  | Female | 5624818.5 | 861612.992 | 4410151.977 | 353053.527 | 15.32 | 78.41 | 6.28 |
| Prevalence | Both | 16454176.5 | 2346846.369 | 12111087.83 | 1996242.305 | 14.26 | 73.6 | 12.13 |
|  | Male | 4759581.27 | 569136.045 | 3092387.423 | 1098057.798 | 11.96 | 64.97 | 23.07 |
|  | Female | 11694595.24 | 1502706.78 | 8912893.071 | 1278995.387 | 12.85 | 76.21 | 10.94 |
| YLDs | Both | 1280180.85 | 206419.052 | 1219855.269 | -146093.471 | 16.12 | 95.29 | -11.41 |
|  | Male | 371861.09 | 51973.452 | 322702.325 | -2814.685 | 13.98 | 86.78 | -0.76 |
|  | Female | 908319.76 | 128197.378 | 886363.355 | -106240.976 | 14.11 | 97.58 | -11.7 |

| **Table S10. The (p, d, q), AIC, and Ljung-Box (*P*) values of the ARIMA model.** | | | | | |
| --- | --- | --- | --- | --- | --- |
| Incidence | Number | (0,2,0) | 733.15 | 734.55 | 0.9987 |
|  | Rate | (2,1,0) | 166.30 | 170.60 | 0.9972 |
|  | Number | (0,2,0) | 654.52 | 655.92 | 0.9998 |
|  | Rate | (2,1,0) | 135.35 | 141.09 | 0.9999 |
|  | Number | (0,2,0) | 654.52 | 655.92 | 0.9999 |
|  | Rate | (2,1,0) | 135.35 | 141.09 | 0.9998 |
| Prevalence | Number | (0,2,0) | 762.03 | 763.43 | 0.9971 |
|  | Rate | (1,1,0) | 200.63 | 203.49 | 0.6107 |
|  | Number | (0,2,0) | 741.44 | 742.84 | 0.9901 |
|  | Rate | (1,1,0) | 212.62 | 215.49 | 0.3974 |
|  | Number | (0,2,0) | 688.31 | 689.71 | 0.9995 |
|  | Rate | (1,1,0) | 175.20 | 178.07 | 0.8809 |
| YLDs | Number | (1,2,0) | 591.39 | 594.19 | 0.9723 |
|  | Rate | (3,1,0) | 31.95 | 39.12 | 0.8869 |
|  | Number | (1,2,0) | 567.39 | 570.19 | 0.8912 |
|  | Rate | (3,1,0) | 40.48 | 47.65 | 0.9529 |
|  | Number | (0,2,1) | 525.20 | 528.01 | 0.9973 |
|  | Rate | (2,1,0) | 9.88 | 14.19 | 0.9442 |

| **Table S11. The projected of incidence of hip fractures attributable to falls in older adults over the next 15 years** | | | | | |
| --- | --- | --- | --- | --- | --- |
| **Both** | | **Female** | | **Male** | |
| **Number of incidence** | **ASIR per 100,000 population** | **Number of incidence** | **ASIR per 100,000 population** | **Number of incidence** | **ASIR per 100,000 population** |
| 12951569 (12858588,13044550) | 922.339 (915.962,928.715) | 9162106 (9093869,9230344) | 1115.205 (1107.424,1122.986) | 3789463 (3764391,3814534) | 635.79 (631.967,639.612) |
| 13227821 (13019910,13435733) | 914.393 (899.522,929.264) | 9342885 (9190302,9495469) | 1102.587 (1084.198,1120.977) | 3884936 (3828874,3940998) | 636.624 (627.888,645.361) |
| 13504074 (13156171,13851976) | 909.23 (885.505,932.956) | 9523664 (9268343,9778985) | 1095.509 (1066.306,1124.712) | 3980410 (3886600,4074219) | 639.151 (625.478,652.823) |
| 13780326 (13271049,14289603) | 906.954 (875.253,938.656) | 9704443 (9330691,10078195) | 1094.096 (1055.737,1132.454) | 4075883 (3938560,4213206) | 643.128 (625.172,661.085) |
| 14056579 (13367015,14746142) | 906.668 (868.28,945.057) | 9885222 (9379160,10391285) | 1096.222 (1050.877,1141.566) | 4171356 (3985420,4357293) | 647.931 (626.491,669.37) |
| 14332831 (13445851,15219811) | 907.337 (863.463,951.211) | 10066001 (9415057,10716945) | 1099.395 (1048.89,1149.9) | 4266830 (4027661,4505999) | 652.997 (628.751,677.243) |
| 14609083 (13508920,15709247) | 908.2 (859.764,956.636) | 10246780 (9439384,11054177) | 1101.893 (1047.427,1156.359) | 4362303 (4065651,4658956) | 657.998 (631.423,684.573) |
| 14885336 (13557305,16213367) | 908.867 (856.502,961.231) | 10427559 (9452934,11402185) | 1103.068 (1045.283,1160.853) | 4457777 (4099681,4815872) | 662.819 (634.218,691.421) |
| 15161588 (13591892,16731284) | 909.231 (853.345,965.116) | 10608338 (9456358,11760319) | 1103.089 (1042.248,1163.931) | 4553250 (4129991,4976509) | 667.479 (637.032,697.926) |
| 15437841 (13613426,17262255) | 909.345 (850.196,968.494) | 10789117 (9450202,12128033) | 1102.493 (1038.659,1166.326) | 4648724 (4156781,5140666) | 672.042 (639.861,704.223) |
| 15714093 (13622542,17805644) | 909.315 (847.073,971.556) | 10969896 (9434933,12504860) | 1101.801 (1034.977,1168.625) | 4744197 (4180223,5308171) | 676.571 (642.732,710.41) |
| 15990346 (13619792,18360900) | 909.231 (844.025,974.438) | 11150675 (9410955,12890396) | 1101.326 (1031.534,1171.118) | 4839670 (4200465,5478876) | 681.105 (645.672,716.538) |
| 16266598 (13605658,18927538) | 909.152 (841.09,977.214) | 11331454 (9378623,13284285) | 1101.15 (1028.46,1173.841) | 4935144 (4217638,5652650) | 685.658 (648.689,722.627) |
| 16542851 (13580570,19505131) | 909.101 (838.283,979.918) | 11512233 (9338252,13686214) | 1101.2 (1025.719,1176.681) | 5030617 (4231857,5829378) | 690.229 (651.781,728.676) |
| 16819103 (13544912,20093294) | 909.078 (835.603,982.553) | 11693012 (9290124,14095901) | 1101.35 (1023.198,1179.502) | 5126091 (4243225,6008956) | 694.811 (654.939,734.683) |

| **Table S12. The projected of prevalence of hip fractures attributable to falls in older adults over the next 15 years** | | | | | |
| --- | --- | --- | --- | --- | --- |
| **Both** | | **Female** | | **Male** | |
| **Number of prevalence** | **ASPR per 100,000 population** | **Number of prevalence** | **ASPR per 100,000 population** | **Number of prevalence** | **ASPR per 100,000 population** |
| 25691970 (25541499,25842441) | 1828.31 (1817.014,1839.605) | 18764828 (18658052,18871604) | 2304.487 (2290.753,2318.22) | 6927142 (6883105,6971179) | 1141.943 (1134.549,1149.338) |
| 26229442 (25892978,26565905) | 1823.484 (1799.951,1847.017) | 19143516 (18904759,19382274) | 2297.272 (2268.941,2325.603) | 7085925 (6987456,7184395) | 1142.37 (1126.311,1158.428) |
| 26766913 (26203902,27329925) | 1819.49 (1782.62,1856.36) | 19522205 (19122687,19921723) | 2291.469 (2247.47,2335.468) | 7244709 (7079938,7409479) | 1142.765 (1116.631,1168.899) |
| 27304385 (26480221,28128549) | 1816.184 (1765.493,1866.875) | 19900893 (19316059,20485728) | 2286.802 (2226.79,2346.813) | 7403492 (7162293,7644691) | 1143.132 (1105.898,1180.365) |
| 27841857 (26725934,28957780) | 1813.448 (1748.82,1878.075) | 20279582 (19487712,21071451) | 2283.048 (2207.094,2359.001) | 7562275 (7235690,7888860) | 1143.472 (1094.378,1192.566) |
| 28379329 (26943926,29814731) | 1811.183 (1732.73,1889.636) | 20658270 (19639695,21676845) | 2280.028 (2188.441,2371.616) | 7721058 (7300975,8141142) | 1143.787 (1082.265,1205.31) |
| 28916800 (27136403,30697198) | 1809.308 (1717.28,1901.337) | 21036959 (19773572,22300345) | 2277.6 (2170.82,2384.38) | 7879842 (7358792,8400891) | 1144.08 (1069.706,1218.454) |
| 29454272 (27305115,31603429) | 1807.757 (1702.487,1913.027) | 21415647 (19890586,22940709) | 2275.647 (2154.186,2397.108) | 8038625 (7409654,8667595) | 1144.352 (1056.816,1231.887) |
| 29991744 (27451500,32531988) | 1806.473 (1688.343,1924.603) | 21794336 (19991755,23596916) | 2274.076 (2138.474,2409.678) | 8197408 (7453982,8940834) | 1144.603 (1043.686,1245.521) |
| 30529215 (27576760,33481671) | 1805.41 (1674.824,1935.997) | 22173024 (20077934,24268114) | 2272.813 (2123.614,2422.011) | 8356191 (7492128,9220254) | 1144.837 (1030.389,1259.285) |
| 31066687 (27681924,34451450) | 1804.531 (1661.9,1947.161) | 22551713 (20149854,24953572) | 2271.796 (2109.536,2434.057) | 8514974 (7524392,9505556) | 1145.054 (1016.986,1273.122) |
| 31604159 (27767884,35440433) | 1803.802 (1649.537,1958.068) | 22930401 (20208145,25652657) | 2270.979 (2096.171,2445.787) | 8673758 (7551037,9796479) | 1145.255 (1003.527,1286.983) |
| 32141631 (27835423,36447838) | 1803.2 (1637.698,1968.702) | 23309090 (20253365,26364814) | 2270.322 (2083.455,2457.188) | 8832541 (7572290,10092792) | 1145.442 (990.051,1300.832) |
| 32679102 (27885235,37472970) | 1802.701 (1626.347,1979.055) | 23687778 (20286005,27089551) | 2269.793 (2071.329,2468.257) | 8991324 (7588355,10394293) | 1145.615 (976.593,1314.636) |
| 33216574 (27917941,38515207) | 1802.288 (1615.45,1989.127) | 24066467 (20306508,27826426) | 2269.368 (2059.739,2478.997) | 9150107 (7599414,10700801) | 1145.775 (963.18,1328.37) |

| **Table S13. The projected of YLDs of hip fractures attributable to falls in older adults over the next 15 years** | | | | | |
| --- | --- | --- | --- | --- | --- |
| **Both** | | **Female** | | **Male** | |
| **Number of YLDs** | **ASYR per 100,000 population** | **Number of YLDs** | **ASYR per 100,000 population** | **Number of YLDs** | **ASYR per 100,000 population** |
| 2314386 (2305814,2322958) | 164.468 (163.788,165.148) | 1682747 (1677009,1688484) | 207.452 (206.677,208.228) | 630572 (627728,633416) | 102.95 (102.447,103.452) |
| 2355178 (2331649,2378708) | 163.849 (162.308,165.389) | 1710743 (1694652,1726833) | 206.403 (204.555,208.251) | 643136 (635388,650884) | 102.607 (101.29,103.924) |
| 2393150 (2348535,2437764) | 163.128 (160.428,165.828) | 1736115 (1705084,1767146) | 205.143 (201.858,208.428) | 655700 (641775,669625) | 102.206 (99.901,104.511) |
| 2429552 (2358441,2500663) | 162.131 (158.255,166.006) | 1759861 (1709708,1810013) | 203.701 (198.89,208.512) | 668264 (647104,689425) | 101.868 (98.564,105.173) |
| 2465082 (2362695,2567469) | 161.189 (156.245,166.133) | 1782598 (1709547,1855648) | 202.359 (196.123,208.594) | 680829 (651508,710150) | 101.659 (97.46,105.858) |
| 2500127 (2362204,2638049) | 160.322 (154.562,166.081) | 1804710 (1705347,1904072) | 201.242 (193.862,208.622) | 693393 (655081,731705) | 101.59 (96.66,106.519) |
| 2534901 (2357602,2712200) | 159.708 (153.413,166.002) | 1826434 (1697656,1955212) | 200.481 (192.31,208.653) | 705957 (657894,754021) | 101.633 (96.14,107.125) |
| 2569525 (2349347,2789703) | 159.334 (152.755,165.913) | 1847918 (1686884,2008953) | 200.08 (191.452,208.707) | 718521 (660000,777042) | 101.74 (95.823,107.657) |
| 2604066 (2337777,2870355) | 159.204 (152.502,165.906) | 1869254 (1673341,2065167) | 199.975 (191.136,208.814) | 731085 (661446,800725) | 101.864 (95.62,108.108) |
| 2638560 (2323151,2953969) | 159.211 (152.465,165.957) | 1890497 (1657266,2123727) | 200.029 (191.113,208.945) | 743650 (662268,825032) | 101.968 (95.456,108.479) |
| 2673028 (2305675,3040381) | 159.252 (152.486,166.018) | 1911682 (1638849,2184515) | 200.079 (191.136,209.022) | 756214 (662497,849931) | 102.031 (95.28,108.782) |
| 2707482 (2285515,3129449) | 159.203 (152.414,165.992) | 1932833 (1618244,2247421) | 199.969 (191.007,208.93) | 768778 (662160,875396) | 102.051 (95.067,109.035) |
| 2741928 (2262808,3221047) | 158.984 (152.139,165.83) | 1953961 (1595575,2312347) | 199.591 (190.591,208.591) | 781342 (661282,901403) | 102.037 (94.815,109.258) |
| 2776369 (2237673,3315065) | 158.554 (151.573,165.535) | 1975075 (1570947,2379204) | 198.907 (189.802,208.013) | 793906 (659883,927930) | 102.003 (94.534,109.472) |
| 2810808 (2210211,3411405) | 157.933 (150.701,165.165) | 1996182 (1544451,2447912) | 197.953 (188.613,207.293) | 806471 (657982,954959) | 101.964 (94.24,109.689) |
